# Sarcopenia and cachexia: molecular mechanisms and therapeutic interventions

**DOI:** 10.1002/mco2.70030

**Published:** 2025-01-05

**Authors:** Tiantian Wang, Dong Zhou, Zhen Hong

**Affiliations:** ^1^ Department of Neurology West China Hospital of Sichuan University Chengdu Sichuan China; ^2^ Institute of Brain Science and Brain‐Inspired Technology of West China Hospital Sichuan University Chengdu Sichuan China; ^3^ Department of Neurology Chengdu Shangjin Nanfu Hospital Chengdu Sichuan China

**Keywords:** cachexia, molecules, pathogenesis, sarcopenia, targeted therapy

## Abstract

Sarcopenia is defined as a muscle‐wasting syndrome that occurs with accelerated aging, while cachexia is a severe wasting syndrome associated with conditions such as cancer and immunodeficiency disorders, which cannot be fully addressed through conventional nutritional supplementation. Sarcopenia can be considered a component of cachexia, with the bidirectional interplay between adipose tissue and skeletal muscle potentially serving as a molecular mechanism for both conditions. However, the underlying mechanisms differ. Recognizing the interplay and distinctions between these disorders is essential for advancing both basic and translational research in this area, enhancing diagnostic accuracy and ultimately achieving effective therapeutic solutions for affected patients. This review discusses the muscle microenvironment's changes contributing to these conditions, recent therapeutic approaches like lifestyle modifications, small molecules, and nutritional interventions, and emerging strategies such as gene editing, stem cell therapy, and gut microbiome modulation. We also address the challenges and opportunities of multimodal interventions, aiming to provide insights into the pathogenesis and molecular mechanisms of sarcopenia and cachexia, ultimately aiding in innovative strategy development and improved treatments.

## Graphical abstract

Sarcopenia is defined as a muscle‐wasting syndrome that occurs with accelerated aging, while cachexia is a severe wasting syndrome associated with conditions such as cancer and immunodeficiency disorders, which cannot be fully addressed through conventional nutritional supplementation. Sarcopenia can be viewed as a component of cachexia, with bidirectional regulation between adipose tissue and skeletal muscle serving as a potential molecular mechanism for both conditions. However, the underlying mechanisms differ. Despite the development of various therapeutic strategies aimed at reversing or preventing sarcopenia and cachexia, their effectiveness has often been limited. In this review, we discuss the abnormal changes in the muscle microenvironment that contribute to the progression of these conditions. We also highlight recent therapeutic approaches, including lifestyle modifications, small molecular agents, and nutritional interventions targeting sarcopenia and cachexia. Additionally, we explore emerging strategies for muscle wasting, such as gene editing, stem cell therapy, and gut microbiome modulation, along with their potential applications and prospects for improving muscle function. Finally, we address the major challenges and opportunities associated with multimodal interventions. We hope this review provides insights into the pathogenesis and molecular mechanisms of sarcopenia and cachexia, ultimately facilitating new methods, drug discovery, and improved treatment options for these conditions.

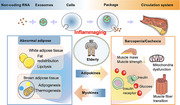

## 1 INTRODUCTION

Rosenberg first defined sarcopenia in 1989 as a “muscle‐wasting geriatric syndrome” characterized by the progressive loss of skeletal muscle mass and strength.^1^, ^2^ Studies indicate that sarcopenia affects 5–13% of adults aged 60–70 years, with prevalence rising to between 11 and 50% in individuals over 80 years old. Sarcopenia is associated with a range of conditions, including cardiac disease, respiratory disease, and cognitive impairment, leading to mobility challenges and a diminished overall quality of life (QOL). It also contributes to a decline in independence, often necessitating long‐term care placement.^3^ Patients face various physical challenges, such as mobility disorders, an increased risk of falls and fractures, reduced capacity to perform daily tasks, disabilities, diminished QOL, loss of independence, and a higher risk of mortality.^4^ Sarcopenia imposes significant social, health, and economic burdens, especially as the elderly population continues to grow. The rising incidence of sarcopenia presents a pressing challenge for the healthcare system. Research has extensively examined hormones, including insulin signaling, inflammatory pathways, and impaired proteostasis, as potential risk factors for sarcopenia.^5^ However, the molecular mechanisms and triggers of sarcopenia remain incompletely understood, which hampers the development of effective therapies. Therefore, investigating the underlying molecular mechanisms and identifying potential therapeutic targets are essential for advancing the clinical management of sarcopenia.

Cachexia is widely recognized as a severe wasting syndrome associated with diseases such as cancer and immunodeficiency disorders, which cannot be fully alleviated through conventional nutritional supplementation.^6^, ^7^ With an estimated annual death rate of 2 million people worldwide, cachexia is a significant contributor to human morbidity and mortality.^8^ Patients with cachexia share a common factor: an unresolved underlying disease process—a wound that does not heal. They experience involuntary weight loss, specifically muscle and fat loss, which may extend to other tissues (e.g., heart and bone), often accompanied by anorexia, fatigue, and sickness behavior such as anhedonia. The diversity of diseases leading to cachexia suggests that either multiple processes converge into an advanced state or various early diseases follow a common pathway of progression.^9^ The uncertainty surrounding the mechanisms driving cachexia necessitates further research to identify the initiators and to understand how they disrupt communication and function across multiple organ systems. Cancer cachexia, commonly observed in older patients, frequently coexists with sarcopenia. It affects approximately 70% of cancer patients and is responsible for up to 22% of cancer‐related deaths.^10^, ^11^, ^12^ In fact, these two syndromes overlap significantly, particularly among the elderly.^10^, ^13^ Most individuals with cachexia are also sarcopenic; however, most sarcopenic individuals are not classified as cachectic, suggesting that sarcopenia can be considered a component of cachexia.^10^, ^13^, ^14^ The mechanisms underlying muscle loss in cachexia and sarcopenia differ.^15^ Sarcopenia is often linked to metabolic disorders, while cachexia is characterized by increased total energy expenditure and basal metabolic rates. Sarcopenia does not typically involve weight loss, in contrast to cachexia, which is associated with significant weight reduction and a decrease in both fat and fat‐free mass.^13^, ^16^, ^17^ Moreover, although both conditions are related to systemic inflammation, cachexia is marked by more intense inflammatory responses, while sarcopenic patients often exhibit mild or undetectable systemic inflammation.^17^, ^18^ Sarcopenia is influenced by various factors, whereas in cachexia, the activation of proinflammatory cytokines plays a major and direct role in disrupting muscle metabolism.^13^ Several questions emerge: should we differentiate our approach to age‐related sarcopenia from that of sarcopenia associated with diseases in older cancer patients? Should we advocate for physical activity and pharmacotherapy for all cancer patients, even those experiencing a negative energy balance? A thorough understanding of these pathological conditions—including their definitions, screening tools, and diagnostic criteria—is crucial for tackling these questions effectively.

Currently, several emerging treatment strategies for sarcopenia and cachexia are being developed. For instance, anti‐inflammatory cytokines have shown promise in alleviating both conditions in rodent models. Additionally, physical activity is recognized as a crucial component in the management of sarcopenia and cancer cachexia.^19^, ^20^ Pharmacotherapy options effective for these conditions include β‐blockers, which can reduce body energy expenditure and enhance substrate utilization efficiency, with some also exhibiting beneficial effects for cachexia.^21^ Notably, GLP‐1 receptor (GLP‐1R) agonists and β3‐adrenergic receptors (β3‐ARs) are particularly promising, as their stimulation has been associated with improvements in muscle wasting, reduction of aging‐related inflammation, enhanced insulin sensitivity in adipocytes.^22^ Given the current limitations of therapies aimed at reversing sarcopenia and cachexia, these strategies may provide potential benefits. Due to the multifaceted nature of both conditions, multimodal therapeutic approaches are preferred, as a single intervention is unlikely to be effective. These approaches typically combine pharmacological treatments, nutritional supplementation, and structured moderate physical exercise programs.

In this review, we first summarize the pathogenesis of sarcopenia and cachexia, followed by an exploration of the common molecular mechanisms underlying both conditions. We emphasize key signaling pathways shared by the two diseases, including myostatin and activin signaling, NF‐κB and signal transducer and activator of transcription 3 (STAT3) pathways, the insulin‐like growth factor 1(IGF‐1)/protein kinase B (Akt)/the mammalian target of rapamycin (mTOR) pathway, mitochondrial dysfunction, and the roles of cytokines (myokines and adipokines). Additionally, we examine the involvement of microRNAs (miRNAs) and long noncoding RNAs (lncRNAs) in the interaction between muscle and adipose tissue, which contributes to the development of sarcopenia and cachexia. The review also evaluates current diagnostic tools, biomarkers, and therapeutic strategies, including emerging treatments. Finally, we discuss future research directions, highlighting the need for innovative, multitargeted approaches to effectively manage these conditions. By integrating the latest research findings and identifying critical gaps, this review aims to bridge academic research with clinical practice, advancing the understanding and treatment of sarcopenia and cachexia.

## 2 PATHOGENESIS OF SARCOPENIA

Sarcopenia arises from a combination of factors, including muscle atrophy, imbalance in muscle regeneration, neuromuscular junction (NMJ) impairments, cellular senescence, low‐grade inflammation, insulin resistance (IR), aged adipose tissue and hormonal changes. Together, these factors lead to significant alterations in body composition, notably a reduction in muscle mass and strength alongside an increase in fat mass. While each factor individually influences muscle and fat quality and quantity, their complex interactions further intensify these changes, compounding their impact on the body.

### 2.1 Muscle atrophy and regeneration imbalance

#### 2.1.1 Satellite cell dysfunction

Sarcopenia is a multifactorial condition, that results in a progressive age‐related loss of muscular size and strength. Decline of muscle regenerative potential in sarcopenia is attribute to decrease in satellite cell number, also known as muscle stem cells (MuSCs).^23^, ^24^ It is conceivable that if satellite cell proliferative capacity could be maintained during aging, sarcopenia would possibly be delayed.^25^, ^26^ However, contrasting findings have also been reported. Victor et al.^27^ suggested that while older mice (20–24 months of age) exhibited a significant decrease in MuSCs and accompanying fiber atrophy, geriatric mice (28–32 months of age) did not show any further loss of MuSCs despite the presence of multiple sarcopenic symptoms. Given the conflicting evidence, the direct causative relationship between the loss of MuSCs and sarcopenia remains controversial. There is a suggestion that sarcopenia and MuSC dysfunction may be separate entities, as sarcopenia is observed to occur prior to any significant impairment in MuSC function.^28^

During the aging process, various internal changes significantly affect the function of MuSCs, including the accumulation of reactive oxygen species (ROS), DNA damage, oxidative stress, as well as altered expressions of signaling molecules such as transforming growth factor‐β (TGF‐β), FGF2, p38, p16Ink4, and the Wnt signaling pathway.^29^, ^30^, ^31^ In addition, reduced autophagy, disruptions in protein homeostasis, altered expressions of molecules like Sprouty1, p27^Kip1^, p53, and the notch signaling pathway could also be observed in MuSCs as they age.^30^ These aging‐related changes collectively lead to a decline in the function of MuSCs, which in turn affects skeletal muscle health and its ability to regenerate. Normally, in healthy muscles, the generation or differentiation of satellite cells into adipocytes is minimal. However, the number of satellite cells per single myofiber may vary,^32^, ^33^ their proliferative capacity,^33^, ^34^ and the adipogenic potential^35^ might change. Taylor‐Jones et al.^35^ found that myoblasts from 23‐month‐old adult mice had an increased adipogenic potential than those from 8‐month‐old adult mice. When proliferating, satellite cells from aged mice are capable of transitioning from a myogenic to a fibrogenic phenotype.^36^ Human skeletal muscle satellite cells obtained from older individuals secreted elevated amount of myokines, including IL1a, IL‐6, IL‐8, and TNF‐α compared with the younger group.^37^ Alteration of these secreted factors can potentially affect multiple tissues and influence metabolic homeostasis. Apart from internal changes, MuSCs could also be influenced by environmental signals, resulting in a reduction in their population and a decline in their functionality. This negatively impacts muscle regeneration and exacerbates sarcopenia (described below). During aging, the environment in which stem cells reside is known to be more proinflammatory, a condition referred to as “inflammaging,” and aged MuSCs undergo cellular senescence, which can lead to the release of senescence‐associated secretory phenotype (SASP) factors that contribute to inflammation, creating vicious cycle.^27^, ^38^ Overall, the MuSCs and muscle microenvironment is believed to play a crucial role in the process of muscle wasting, suggesting its prominent influence.

#### 2.1.2 Impaired muscle protein synthesis and increased degradation

The main factor in maintaining the quality of skeletal muscle is the balance between muscle protein synthesis and degradation. With advanced age, the balance will be broken. When the decomposition rate exceeds the synthesis rate, muscle protein will be lost, causing impaired muscle mass or activity.^39^

Protein synthesis in skeletal muscle is primarily induced during normal growth, such as through growth factors and growth hormone, or by conditions like resistance exercise. The main anabolic signal in skeletal muscle is IGF‐1.^40^ When IGF‐1 binds to its transmembrane tyrosine kinase receptor on myocyte membranes, it triggers intracellular trans‐phosphorylation and creates a docking site for insulin receptor substrate 1 (IRS‐1). Activation of this receptor phosphorylates IRS‐1, initiating the phosphatidylinositide 3‐kinases (PI3K)/Akt pathway. This pathway activation leads to increased protein synthesis and muscle hypertrophy by inhibiting glycogen synthase kinase‐3 (GSK3), activating mTOR complex‐1 (mTORC‐1), promoting mTORC‐1‐mediated phosphorylation of p70S6 kinase, and inhibiting IF‐4E‐binding protein.^41^ Furthermore, PI3K/Akt activation prevents protein degradation in skeletal muscle by phosphorylating and inactivating O‐type forkhead box (FOXO) transcription factors. FOXO1, FOXO3a, and FOXO4 are expressed in skeletal muscle and, when phosphorylated, are unable to translocate into the nucleus, thereby preventing the upregulation of proteolysis‐related genes like muscle RING‐finger protein‐1 (MuRF‐1) and muscle atrophy F‐Box (MAFbx, Atrogin‐1).^42^, ^43^ In summary, IGF‐1 stimulates protein synthesis and muscle hypertrophy while also suppressing protein degradation in skeletal muscle, thereby promoting muscle growth and maintenance in response to growth stimuli and exercise.^5^

Proteolysis in skeletal muscle is primarily activated by two signaling pathways: the p38 mitogen‐activated protein kinase (MAPK) pathway and the NF‐κB pathway. Additionally, skeletal muscle mass loss can be induced by the myokine myostatin, which binds to the activin receptor on myocytes. This binding leads to the phosphorylation and activation of SMAD2 and SMAD3 transcription factors, resulting in the upregulation of Atrogin‐1 and MuRF‐1, and the suppression of PI3K/AKT activity. This activity allows FOXO transcription factors to stimulate autophagy, promote the expression of Atrogin‐1 and MuRF‐1, and upregulate other mediators of muscle catabolism.^5^, ^44^ Atrogin‐1 and MuRF‐1 function as E3 ubiquitin ligases and play pivotal roles in the progression of muscle atrophy. Knockout mouse models for each gene have demonstrated resistance to muscle atrophy induced by denervation.^45^ The equilibrium between protein synthesis and degradation in skeletal muscles is crucial for regulating muscle mass and strength.

### 2.2 Role of inflammation and oxidative stress

#### 2.2.1 Chronic low‐grade inflammation (inflammaging)

As the above studies have summarized, chronic inflammation along with aging, termed “inflammaging,” is a common ground for various aging‐related diseases, including osteoporosis, osteoarthritis (OA), and sarcopenia.^46^, ^47^, ^48^, ^49^, ^50^, ^51^, ^52^ When kept under a certain threshold, chronic inflammatory stimulation should not always be harmful due to its secondary adaptive activation of anti‐inflammatory networks.^53^, ^54^, ^55^, ^56^ Adaptive responses influence aging trajectories and outcomes, resulting in unsuccessful aging and age‐associated diseases rather than normal aging and longevity. Active and dynamic mechanisms are responsible for activating anti‐inflammatory responses, especially for preventing and treating harmful inflammation.^57^

However, during muscle atrophy, both proinflammatory and anti‐inflammatory cytokine expression can be altered, leading to increased catabolism and suppression of protein synthesis in skeletal muscle cells, resulting in sarcopenia.^49^, ^58^ Thus, inflammatory cytokines play an important role in sarcopenia.

#### 2.2.2 Oxidative damage to muscle cells

ROS are continuously produced in cells under normal conditions by various enzymes, such as xanthine oxidase and NAD(P)H oxidase, but primarily as by‐products of mitochondrial oxidative phosphorylation. Cells employ several detoxifying mechanisms to maintain redox balance, including the antioxidant enzyme network comprising superoxide dismutase, catalase, and glutathione peroxidase. During aging, this carefully regulated balance between prooxidants and antioxidants is disrupted due to a decline in antioxidant enzymes, leading to increased ROS levels and oxidative damage to mitochondrial DNA (mtDNA).^59^ The heightened production of ROS is believed to contribute to sarcopenia by destabilizing mitochondria in skeletal muscle fibers, thereby making them more susceptible to apoptotic stimuli and downregulating pathways related to mitochondrial biogenesis.^60^, ^61^ Additionally, oxidative stress may drive other processes associated with sarcopenia, including proteolysis, upregulation of TNF‐α levels, and inhibition of muscle cell differentiation.^62^, ^63^ Overall, these findings suggest that redox homeostasis—both in skeletal muscle and motor neurons—may play a critical role in the development of sarcopenia.

### 2.3 Hormonal regulation and metabolic factors

#### 2.3.1 Decline in anabolic hormones

The progressive decline in testosterone levels, averaging 1–2% per year after the age of 30 years,^64^ may be linked to clinical symptoms of hypogonadism, including reductions in muscle mass, strength, and physical performance.^65^ Testosterone is regarded as a key factor in maintaining muscle mass and function during aging.^66^ However, no studies have specifically investigated testosterone supplementation in the context of sarcopenia or among sarcopenic patients. Thus, the efficacy and safety of testosterone supplementation for sarcopenia remain undetermined. Similarly, there have been no randomized controlled trials (RCTs) examining the effects of estrogens in sarcopenic patients. While several studies have evaluated the impact of estrogens on muscle mass, strength, and physical performance in the context of osteoporosis in postmenopausal women—who are typically younger than the usual sarcopenic population—the benefits of estrogens in preventing muscle loss and strength decline are not consistently confirmed in most RCTs, as highlighted by various reviews and meta‐analyses.^67^, ^68^, ^69^

#### 2.3.2 IR and its impact on muscle metabolism

Skeletal muscle plays a crucial role in postprandial glucose uptake, accounting for 60–80% of glucose removal from the bloodstream in response to insulin.^70^ Consequently, skeletal muscle is vital for regulating systemic glucose homeostasis. Impairments in skeletal muscle function, are characterized by reduced glucose uptake and disrupted insulin signaling pathways. These dysfunctions can result in impaired mitochondrial biogenesis and further dysfunction within skeletal muscle.^71^ Moreover, disruption of skeletal muscle has been linked to obesity and IR in mouse models.^72^, ^73^, ^74^

### 2.4 Neuromuscular junction

Sarcopenia is characterized by various changes in the NMJ, including motor endplate dispersion, a decrease in the number of presynaptic vesicles and acetylcholine receptors (AChRs), as well as reduced binding affinity to AChRs. Severe motor neuron diseases associated with muscle, such as amyotrophic lateral sclerosis,^75^ multiple sclerosis,^76^ and duchenne muscular dystrophy^77^ display NMJ defects. This suggests that preserving the integrity of the NMJ may play a crucial role in the treatment of sarcopenia and various neuronal disorders.

The relationship between aging and NMJ dysfunction in muscle remains unclear, but factors such as inflammation‐aging, oxidative stress, and mitochondrial dysfunction are believed to be associated with NMJ functional impairment.^78^ Although alterations in the NMJ are recognized as significant features of sarcopenia,^79^, ^80^ there remains debate regarding whether these pathological events originate within sarcopenia itself or contribute to its development.

### 2.5 Cellular senescence

The accumulation of senescent cells in skeletal muscle is recognized as a significant contributor to the development of sarcopenia.^81^ Cellular senescence represents a state of irreversible cell cycle arrest induced by various stresses and pathophysiological processes such as DNA damage, oxidative stress, proteome instability, telomere shortening, and impaired autophagy.^50^, ^82^, ^83^, ^84^ Senescent cells can be identified by specific markers including senescence‐associated beta‐galactosidase (SA‐β‐gal), and cell cycle inhibitory proteins like p16INK4a and p21Cip1.^84^ These cells are characterized by significant chromatin alterations and the development of SASPs, which includes cytokines, chemokines, matrix‐remodeling proteins, and growth factors that can negatively impact tissue function.^82^, ^83^, ^84^ Initially, senescent cells can trigger cellular senescence in nearby healthy cells through the secretion of SASP factors and via gap junction‐mediated cell–cell interactions.^85^, ^86^ Moreover, SASP can enter the bloodstream, worsening inflammaging.

Recent research indicates varying susceptibility to senescence among different cell populations within skeletal muscle with age. Research has demonstrated that aged MuSCs are more prone to undergoing senescence or apoptosis compared with their younger counterparts.^87^ Besides, fibro/adipogenic progenitors (FAPs) have been found to exhibit high expression levels of p16INK4a, suggesting these cells are prone to entering a senescent state. On the other hand, myofibers in the skeletal muscle of aged mice show elevated expression of p21.^88^ Further, in this study, the analysis of human skeletal muscle has revealed significant inverse associations between the enrichment of SASP‐related pathways and leg strength in older compared with younger skeletal muscle. SIRT1 is a key antisenescence gene, as well as other genes involved in aging and the MAPK signaling pathway. Rathbone et al.^89^ reported that overexpression of SIRT1 increases satellite cell proliferation, indicating that elevated SIRT1 expression might rescue an aging‐induced reduction in satellite, cell numbers and regenerative capacity in older muscle models. In support of this, combined resistance exercise with an activator of SIRT1 (resveratrol) showed elevated satellite cell proliferation and increased muscle fiber size and function of older humans to a greater extent than resistance exercise alone.^90^ Moreover, resveratrol can prevent cell death and stimulate the differentiation of myotubes, while silencing SIRT1 evokes greater cell death and decreased differentiation.^91^

### 2.6 Aged adipose tissue

**FIGURE 1:**
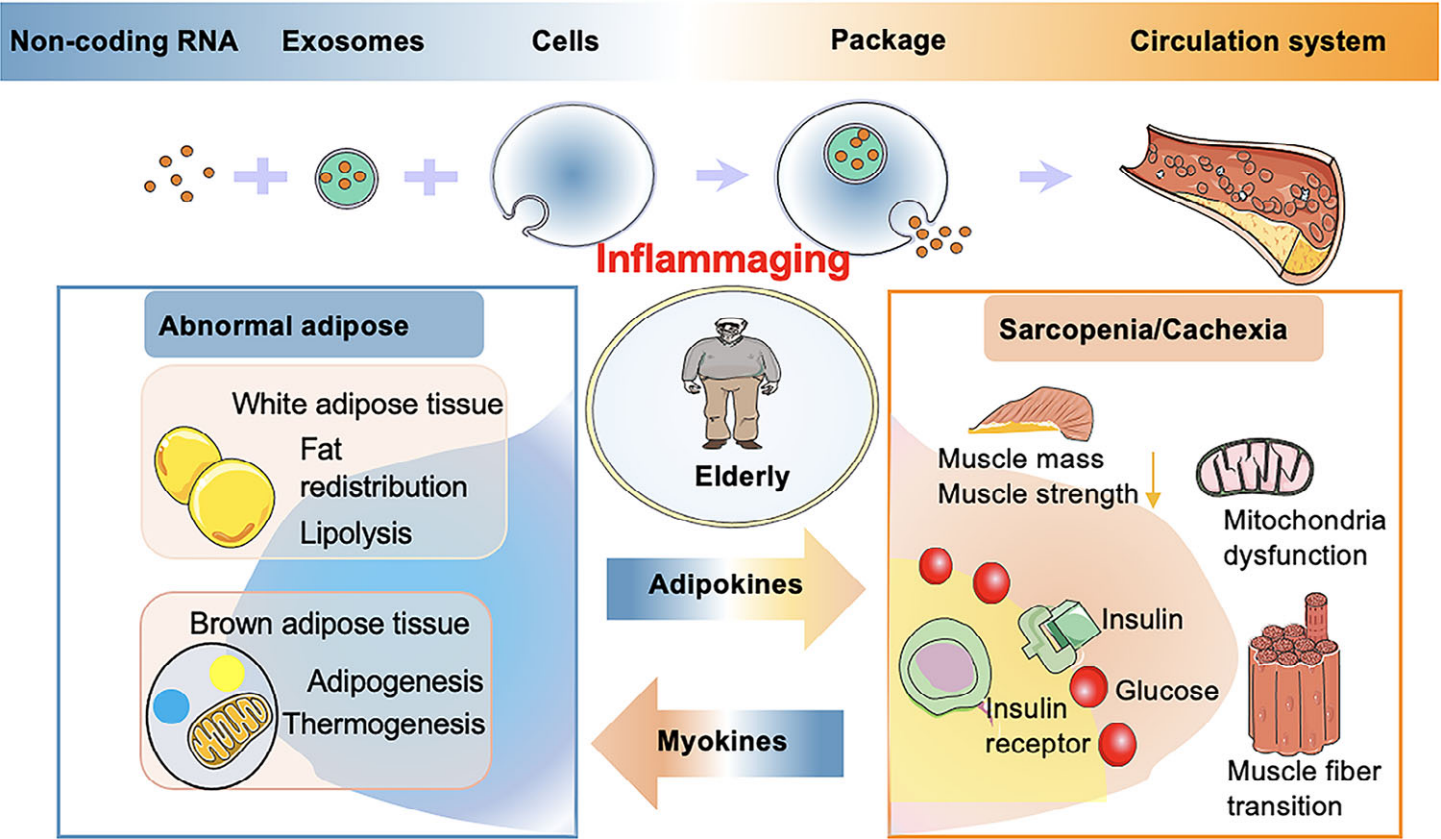
The bidirectional regulation between adipose tissue and skeletal muscle occurs through the endocrine pathway. The changes in cytokines and noncoding RNAs induced by aged adipose/muscle lead to unfavorable fat deposition and accumulation, persistent low‐grade inflammation, and impairments of lipolysis, glucose uptake, and insulin sensitivity, contributing to the pathophysiology of sarcopenia and cachexia.

As previously summarized, aged adipose tissue may be a risk factor for sarcopenia^22^, ^49^, ^50^ (Figure 1). Fat tissue serves as a significant endocrine organ and could potentially be the largest organ in aging or obese individuals in certain instances.^92^ The primary components of adipose tissue include white adipose tissue (WAT), along with brown adipose tissue (BAT) and the intermediate beige fat.^93^, ^94^ WAT is also present within muscles and impacts muscle functionality.^95^, ^96^, ^97^, ^98^, ^99^, ^100^ BAT and WAT have contrasting roles in energy metabolism.^101^

#### 2.6.1 BAT

BAT generates small, numerous fat droplets dispersed in the cytoplasm. Abundant mitochondria within BAT facilitate the burning of fat to generate heat. BAT expresses high levels of uncoupling protein‐1 (UCP‐1), crucial for heat production. Importantly, BAT can metabolize WAT, aiding in obesity alleviation by producing heat energy.^102^, ^103^, ^104^ Thus, BAT holds considerable importance in promoting human well‐being and combating conditions like diabetes, obesity, and other metabolic ailments.^105^, ^106^, ^107^, ^108^ Indeed, brown fat adipocytes originate from skeletal muscle precursor cells, specifically Myf5‐positive cells, whereas beige adipocytes differentiate from Myf5‐negative progenitor cells, despite exhibiting similar morphology and function to brown adipocytes.^109^, ^110^

As individuals age, there is a decline in BAT mass and adipogenic activity, coupled with reduced BAT thermogenesis. This decline may exhibit sex‐based differences.^111^ For example, Ouellet's study^112^ demonstrated a significant decline in BAT mass among the elderly compared with the young cohort. Additionally, this decline was more pronounced in males across all age groups than in females.^112^ Valle's research^113^ indicated that the capacity of BAT to sustain reduced heat production is poorer in elderly male rats compared with females. These findings may help explain the increase in visceral fat accumulation with age and why women tend to be more resilient to cold conditions compared with men.^114^ Indeed, the underlying cause of decreased BAT activity during aging remains elusive. Such changes could potentially exacerbate the development of obesity and IR, both hallmarks of sarcopenia. However, the interplay between BAT and sarcopenia warrants further investigation for clarity. Furthermore, BAT possesses endocrine capabilities that influence metabolism in distant organs, releasing factors that function in an autocrine manner. For example, BAT can secrete not only traditional adipokines such as leptin and adiponectin, but also FGF21 and 12,13‐HOME. Aging may affect the signaling of these molecules, which in turn could influence skeletal muscle physiology.^101^, ^115^, ^116^ We will describe this insight in the following section.

#### 2.6.2 WAT

WAT is associated with insulation and cushioning, lipid storage and release, and endocrine signaling. It is extensively present in subcutaneous tissue (sWAT) and surrounding viscera (vWAT). The varying morphological and molecular mechanisms are fundamental to these two depots, directly impacting distinct metabolic health and diseases.^117^, ^118^ vWAT is characterized by numerous large adipocytes, whereas sWAT contains a higher proportion of small adipocytes.^119^ vWAT contains a large number of blood vessels, and nerves are more susceptible to lipolysis in response to signaling from the nervous system, with a rich blood supply, while sWAT is preferentially for long‐term lipid storage.^120^ As individuals age, there is a tendency for the ratio of vWAT to sWAT to rise, reflecting the accumulation of vWAT.^121^ The substantial release of free fatty acids (FFAs) directly into the portal vein circulation results in their dissemination to the liver and peripheral tissues, contributing to triglyceride (TG) accumulation in these nonadipose tissues and reducing insulin sensitivity. This process might explain why increased vWAT is related to metabolic diseases, including diabetes and obesity.^122^, ^123^

Furthermore, the maintenance of WAT functionality relies on precise regulation to produce lipid‐storing adipocytes. Adipose progenitor and stem cells (APSCs) play a pivotal role in metabolic regulation by generating mature adipocytes. These APSCs are proposed to be central to systemic metabolism disruption. Specifically, their capacity for proliferation and differentiation sustains the renewal, enlargement, and adaptive functionality of adipose tissue.^124^ However, the decreased proliferation and differentiation capacity of APSCs mainly occur due to aging.^92^, ^125^ The dysfunction of APSCs compromises the plasticity of adipose tissue and is correlated with an increased susceptibility to IR.^126^ Additionally, preadipocytes' ability to manage fatty acids diminishes with age, as they are less capable of differentiating and effectively storing lipids. This impairment can result in a higher vulnerability to lipotoxicity and reduced efficiency in fatty acid‐induced adipogenesis.^127^ Certainly, these factors could contribute to age‐related metabolic alterations in older individuals. The mechanism underlying impaired preadipocyte differentiation involves the downregulation of C/EBPα and peroxisome proliferator‐activated receptor (PPAR)γ expression in adipose tissue.^128^, ^129^ Indeed, beyond differentiation, the self‐renewal capacity of preadipocytes is also compromised during aging or degenerative diseases.^126^

Moreover, aging induces senescence in both rodent and human WAT, leading to a reduction in adipogenic potential.^130^ For instance, markers like p16INK4a and SA‐β‐gal are heightened throughout WAT depots, and the ability of preadipocytes to differentiate is diminished in older mice.^131^ Clearance of senescent cells from 18‐month‐old mice by Xu alleviated the senescent cell‐mediated inhibition of adipogenesis and mitigated age‐related fat loss.^132^ This suggests that senolytic drugs could serve as a promising treatment to diminish the burden of senescent cells and enhance therapeutic potential in adipose tissue.^133^ Senescent cells, including adipocytes and preadipocytes, can secrete SASP factors such as proinflammatory cytokines, which can induce inflammation in tissues and impact other organs. When adipose progenitor cells are cocultured with aging cells, only around 20% of these progenitor cells accumulate fat. In contrast, when cocultured with nonaging cells, the proportion of progenitor cells with fat accumulation increases to over 50%. This suggests that the presence of senescent cells can impair the adipogenic potential of progenitor cells.^132^ Indeed, immune cells within adipose tissue can release proinflammatory cytokines under the influence of aging or obesity. The aging process is characterized by an increase in the number of M1 phenotype macrophages and a decrease in M2 phenotype macrophages in WAT. For example, animal studies have demonstrated that as animals age, the ratio of M1 to M2 macrophages rises, signifying a decrease in M2 alternatively activated macrophages and fostering a proinflammatory environment within the tissue.^134^, ^135^

Overall, age‐related factors, such as the redistribution of WAT, senescence, and inflammation, can compromise the normal functions of adipose tissue. These disruptions can lead to abnormal lipid accumulation, metabolic dysfunction, and IR in WAT. This review categorizes the process of fat infiltration and delineates the events occurring at each stage, in conditions during aging. However, it is noteworthy that while certain cells or molecules play a pivotal role in a particular phase, they may also contribute to the overall process of muscle homeostasis. In addition, lean and youthful WAT produces various molecules like leptin and adiponectin that can affect other organs or the adipose tissue itself. As inflammation and reduced adipogenesis become more common with aging, they might also impact the endocrine role of adipose tissue. The production and secretion of these adipokines are influenced by aging, which has been explored in depth in the literature.^118^, ^136^ We will discuss the roles of adipokines in sarcopenia with aging in the following section.

##### Accumulation of lipid derivatives in muscle cells leads to the condition known as myosteatosis

At first, it is hypothesized that an excess of lipids can “spill over” into various tissues, leading to ectopic fat infiltration, notably in skeletal muscles, a phenomenon termed myosteatosis. These lipids accumulate in the form of intermuscular adipose tissue (IMAT), intramuscular adipose tissue, and intramyocellular lipid droplets (IMCLs).^49^, ^137^ Evidence indicates that myosteatosis is linked to preceding muscle atrophy, contributing to the development of sarcopenia.^138^ Although the precise biological mechanisms driving the escalation of myosteatosis or the infiltration of fat into skeletal muscles remain uncertain, recent findings propose a significant involvement of FAPs. FAPs, constitute a subset within the muscle with the capacity for differentiation into adipocytes and fibrocytes.^137^ The process of differentiation hinges on various stimuli, potentially leading to either excessive fat infiltration or fibrosis, both commonly observed in various pathological conditions.^137^ FAPs have a direct role in regulating muscle regeneration. For example, depletion of FAPs in muscles has been demonstrated to cause muscle weakness and atrophy, alterations in fiber type composition, and denervation at NMJ, ultimately leading to a failure to maintain muscle mass.^139^ With aging, aberrant signaling in FAPs can trigger their profibrotic behavior, leading to skeletal muscle fibrosis. Additionally, this process can compromise satellite cell function.^140^ Transplantation of young FAPs into aged mice has shown the ability to restore the myogenic commitment of MuSCs.^140^, ^141^, ^142^ Interestingly, transplanting senescent FAPs intraperitoneally into mice is sufficient to induce a sarcopenia‐like functional phenotype, as evidenced by decreased grip strength and walking speed.^139^, ^143^

FAPs exert many of their functional effects on muscle regeneration and repair by secreting paracrine factors within their local microenvironment.^144^, ^145^ Aging could potentially impair the secretion of a soluble myogenic support signal from FAPs. For instance, FAPs may lose the ability to stimulate the production of the matricellular protein Wnt1‐inducible signaling pathway protein 1 (WISP1), crucial for satellite cell expansion and commitment. This impairment could further contribute to the decline in muscle function with age.^140^ In addition, FAPs serve as the main mononuclear cell source of IL‐33, a cytokine linked to type 2 immunity. Nonetheless, with aging, there is a decline in IL‐33 production by FAPs, resulting in reduced accumulation of regulatory T cells (Tregs) and impaired muscle repair.^146^ Studying the mechanisms that lead to these alterations in IL‐33 production by FAPs is crucial for comprehending the broader effects of FAPs on muscle regeneration and repair as one ages.^140^, ^146^ Modulating the cytokines released by FAPs could serve as a potential therapeutic approach to alleviate age‐related muscle decline and promote muscle regeneration. Additionally, changes in the microenvironment or niche where FAPs reside during aging contribute to the dysfunctional state of FAPs, disrupting the cellular niche necessary for proper skeletal muscle regeneration.^147^, ^148^ The differential effects of conditioned medium from myogenic cells isolated from aged versus young donors on FAPs proliferation and adipogenic differentiation highlight the impact of age‐related changes in the muscle microenvironment.^147^ The disruption described can indeed lead to the development of muscle fibrosis. Furthermore, in the presence of chronic inflammatory conditions associated with various metabolic disorders, the signals that typically regulate FAP apoptosis and inhibit proliferation are disturbed. This disturbance results in a chronic state of tissue remodeling, ultimately leading to the accumulation of fibrotic and fatty tissues. This aberrant tissue accumulation further impairs skeletal muscle function and regeneration, exacerbating the effects of aging and metabolic disorders on muscle health. Understanding and addressing these mechanisms are crucial for developing effective therapeutic interventions.^145^

Aged FAPs not only contribute to the decline in muscle repair and regeneration but are also the main stem cell group involved in the formation of IMAT in sarcopenia.^137^, ^139^, ^149^ Numerous signaling pathways are implicated in this process. With aging, there is a diminished response in the Notch signaling pathway, resulting in compromised satellite cell proliferation and hindrance in muscle regeneration.^150^ The reduced activity of the Notch signaling pathway may be implicated in the development of IMAT driven by FAPs during aging.^150^ In specific, Notch signaling has been observed to inhibit the differentiation of FAPs into adipocytes both in laboratory settings and in living organisms. Treatment with 5 µM DAPT, an activator of the Notch signaling pathway, has been shown to reduce the accumulation of adipose tissue.^150^ Moreover, during aging, changes in Wnt10b signaling lead to the increased expression of critical adipogenic genes, thereby contributing to the accumulation of IMAT.^151^ Aging is also linked to a decline in the synthesis of nitric oxide (NO) in skeletal muscle. NO plays a role in regulating the fate of FAPs by inhibiting their differentiation into adipocytes.^152^, ^153^ Moreover, aging is associated with comorbidities that can influence the behavior of FAPs. Buras et al.^154^ found that prolonged consumption of a high‐fat diet (HFD) leading to obesity can promote the proliferation of FAPs, as well as their differentiation into adipocytes and collagen‐depositing fibroblasts. This observation is reinforced by the discovery that adipogenic progenitors can be isolated from obese human skeletal muscle using the CD56^neg^CD15^pos^ cellular fraction, which corresponds to PDGFRα^pos^ FAPs that give rise to intramuscular adipose tissue.^155^, ^156^ In obese mice, the proliferation of FAPs is facilitated by elevated levels of circulating adipokines such as Thrombospondin 1 (THBS1) and TGF‐β1, which are secreted by the hypertrophic adipose tissue.^154^, ^157^ The presence of various factors derived from adipocytes enhances the adipogenic differentiation of FAPs, suggesting a direct mechanism by which adipose tissue expansion in obesity contributes to the accumulation of IMAT. Moreover, diabetes could also influence the behavior of FAPs. A study utilizing various genetic and diet‐induced mouse models of diabetes demonstrated that the ectopic deposition of adipocytes in skeletal muscle originated from FAPs.^158^

The adipocytes derived from FAPs may display reduced insulin sensitivity compared with typical adipocytes, as indicated by decreased insulin receptor phosphorylation. This suggests that the accumulation of adipocytes originating from FAPs could contribute to compromised peripheral insulin sensitivity.^156^ Indeed, these findings indicate that FAPs directly contribute to the buildup of IMAT in skeletal muscle during aging and metabolic disorders, which are pivotal to the adverse alterations observed in skeletal muscle under metabolic stress. However, since IMAT represents only a small portion of the overall adipose depots in the body, its adverse effects on glucose disposal are likely mediated through secondary mechanisms that hinder myofiber's capacity to uptake glucose (as described below).

As previously mentioned, FAPs can develop into IMAT during aging, which refers to adipose tissue interspersed between and around skeletal muscle groups. Several studies highlight a relationship between IMAT levels and muscle function. Indeed, increased accumulation of IMAT can lead to a decrease in muscle mass, muscle strength, and insulin sensitivity.^100^, ^159^, ^160^ These research findings indicate that individuals aged 70–79 years with elevated baseline levels of IMAT are more prone to experiencing mobility limitations compared with those with lower baseline levels of IMAT.^160^ Studies indicate that higher levels of IMAT in the quadriceps upon admission are more strongly correlated with poorer recovery of activities of daily living (ADL) than lower muscle mass in older hospitalized patients.^161^ These studies illustrate that IMAT serves as a potential predictor of sarcopenia, a condition observed in diverse populations, including obese, diabetic, dystrophic, sarcopenic, and aging animals and patients.^158^, ^162^, ^163^ IMAT is linked to metabolic health and the onset of IR and other metabolic disorders.^164^, ^165^ The proximity of IMAT to muscle fibers enables it to release various proinflammatory cytokines such as IL‐6 directly onto the muscle fibers, triggering local inflammation. This inflammatory reaction can contribute to the onset of IR and other metabolic disturbances.^99^, ^100^, ^166^ Moreover, muscle dysfunction can contribute to a detrimental cycle where reduced physical activity leads to increased levels of IMAT, which in turn exacerbates muscle dysfunction.^167^, ^168^, ^169^ Further exploration is necessary to comprehensively grasp the precise mechanisms by which IMAT impacts skeletal muscle.

Given the possible mechanistic connection between the adverse effects of IMAT accumulation on muscle and mobility function, addressing IMAT and its related inflammation could prove pivotal in rehabilitation settings addressing age‐related diseases and disabilities.^170^, ^171^, ^172^ Promising evidence indicates that physical therapy may have beneficial effects on levels of IMAT and intramuscular inflammation.^173^, ^174^ Other researchers examined the impact of 12 weeks of eccentric exercise training on older individuals and observed an 11% reduction in the area of IMAT in the thigh.^175^ A study has shown that a 14‐week periodized conventional strength training protocol can have positive effects on intermuscular fat and muscle quality in patients with knee OA.^176^ In a captivating study conducted by Wroblewski et al.,^177^ attention was directed toward elderly elite athletes who participated in fitness and sports competitions at least four or five times per week. The results unveiled that these individuals did not undergo any decline in lean muscle mass or a rise in IMAT accumulation with age.^177^

Aside from IMAT, fat infiltration into muscle can manifest as IMCLs. IMCLs, a smaller subset of lipids, are stored as lipid droplets within muscle cells.^98^ Predominantly containing TGs, diacylglycerols (DAGs), and ceramides, these lipids serve as an energy source during exercise. However, their excessive accumulation can induce lipotoxic and inflammatory effects, heightening the risk of certain pathological conditions, particularly metabolic diseases.^178^, ^179^ The primary consequence of muscle‐associated LDs is the degradation of mitochondrial function in muscle, leading to reduced lipid β‐oxidation and lipolysis, along with increased ROS production. These changes foster IR and lipotoxicity, as well as activate inflammatory signaling pathways, ultimately resulting in skeletal muscle inflammation, metabolic abnormalities, and contributing to the onset of sarcopenia.^49^, ^98^, ^180^, ^181^, ^182^, ^183^, ^184^ Over time, these alterations negatively impact the contractile function and metabolic characteristics of skeletal muscle, significantly affecting human health and exacerbating IR.^81^

Myosteatosis induced by IMAT and IMCL can result in local muscle inflammation and alters myocyte insulin sensitivity through paracrine or autocrine effects. Inflammatory signals can activate pattern recognition receptors in myocytes, such as Toll‐like receptor 4 (TLR4), leading to direct metabolic effects.^119^ Besides, MuSCs gradually undergo senescent, releasing SASP factors.^27^, ^38^ Therefore, muscle tissues can trigger metabolic dysfunction and inflammaging by transplanting proinflammatory cytokines into blood‐storm, contributing to IR and inflammation.^49^ Collectively, this evidence contributes to the recognition of myosteatosis as a potential significant predictor in sarcopenia.

##### Aging‐induced myosteatosis can exacerbate systemic inflammaging and IR by recruiting immune cells

As described earlier, age‐induced myosteatosis plays a significant role in contributing to IR and inflammation.^185^ The elevated levels of proinflammatory cytokines are recognized for inducing the infiltration of immune cells, such as T cells, B cells, and macrophages, into adipose tissue, muscle, and other tissues.^51^, ^52^, ^186^ Furthermore, age‐related immune system dysregulation, known as immune aging or immunosenescence, can disturb the immune environment. This disruption alters interactions between immune cells and signaling pathways crucial for skeletal muscle function.^187^, ^188^ For instance, Wang et al.^189^ conducted bone marrow transplantations in mice with disparate ages and observed that immune system aging led to a reduction in MuSC numbers. This process promoted their transformation into a fibrogenic phenotype, ultimately impacting the onset of sarcopenia.^189^

The lymphocyte compartment encompasses the primary circulating subpopulations of immune cells.^190^ Within lymphocytes, T cells are pivotal in the processes of skeletal muscle repair, regeneration, and differentiation. Specifically, CD8+ T cells contribute to skeletal muscle regeneration through the secretion of MCP‐1, which recruits Gr1(high) macrophages. These macrophages, in turn, support myoblast proliferation, aiding in the regeneration process.^191^ During immune aging, the decline and alteration in the phenotype of T lymphocytes, transitioning from CD8+ to CD4+, could be associated with a decrease in muscle mass.^192^ Research has indicated that a specific subset of CD4+ T helper 1 cells, characterized by the absence of CD28 (CD4+CD28null T cells), showed a negative correlation with muscle mass index in patients with sarcopenia.^192^, ^193^ Furthermore, CD28null T cells have been found to release cytotoxic particles containing perforin and granzyme B. Additionally, they can produce cytokines like TNF‐α and interferon gamma (IFN‐γ), thereby activating inflammatory pathways.^194^ The cytokines produced by CD28null T cells, are believed to contribute to the onset of sarcopenia. Additionally, studies have indicated that the secretome of activated T cells from younger individuals can stimulate the proliferation and movement of immortalized murine satellite cells. Conversely, the secretome from activated T cells of older individuals has been observed to induce premature differentiation, while leaving satellite cell proliferation and migration unaffected. This suggests a modified interaction between immune cells and satellite cells during aging, potentially impacting skeletal muscle regeneration and repair processes in sarcopenia.^195^ These observations imply that proteins released by adaptive immune cells in younger individuals promote the proliferation and movement of satellite cells, whereas proteins secreted by adaptive immune cells in older individuals hinder satellite cell proliferation and migration by triggering premature differentiation. Consequently, the diminished proliferation and migration of satellite cells in older individuals are associated with age‐related deficiencies in T cells. In vitro investigations have additionally revealed that aged muscle cells exhibit reduced responsiveness to immune secretions.^196^ Dumke's study^196^ discovered that a medium conditioned by T cells stimulated the proliferation and migration of satellite cells taken from the muscles of young rats but did not yield the same outcomes with satellite cells obtained from the muscles of older rats. This suggests that both the reaction of satellite cells to immune cells and the immune response to muscle cells alter with age, fostering a vicious cycle that disrupts muscle regeneration as individuals age.^196^

Additionally, another significant subset of T cells that infiltrate skeletal muscle are known as immune response Tregs, denoted as the CD4 + Foxp3 + sub‐phenotype.^197^ Age‐related impairments to Treg signaling are also believed to limit muscle regenerative capacity. During muscle regeneration, as described above, the recruitment of Tregs via IL‐33 signaling is decreased in old mice due to ineffective production of it by FAP‐like cells, and these defects compromise muscle regenerative capacity.^146^ However, the regenerative capacity of aged skeletal muscle can be improved through intramuscular or systemic supplementation with IL‐33, which re‐establishes the recruitment of Tregs into injured muscles.^146^ The depletion of Tregs, which is characterized by an increased IFN‐γ response and activation of M1 macrophages, leads to an increase in muscle inflammation. It also involves abnormal inflammatory morphology and fibrosis of regenerated muscle fibers.^198^ Studies have found that accumulation of Treg cells in injured skeletal muscle profoundly declines with age, paralleling a degradation of repair and regeneration processes.^146^

Indeed, macrophages play a crucial role in tissue repair following damage. They swiftly infiltrate damaged tissue, aiding in debris removal and promoting satellite cell myogenesis, which contributes to tissue regeneration.^199^, ^200^ Proinflammatory macrophages indeed play a pivotal role in promoting the proliferation and migration potential of satellite cells. The signaling molecules released by these macrophages enhance the regenerative capacity of satellite cells, crucial for muscle tissue repair. However, it is vital to acknowledge that prolonged presence or inhibition of proinflammatory macrophage activity can have adverse effects, potentially exacerbating tissue damage and causing delays in the muscle repair process.^201^ Macrophages possessing anti‐inflammatory properties release substances that guide the differentiation of satellite cells and aid in the reconstruction, remodeling, and maturation of the extracellular matrix (ECM). However, akin to the negative impacts of prolonged proinflammatory activity on muscle health, an excessive or premature shift toward an anti‐inflammatory phenotype can also have detrimental effects. Balancing the inflammatory response is critical for optimal muscle repair and regeneration.^202^ Therefore, achieving a timely and accurate shift to anti‐inflammatory macrophage phenotype replacement is essential for promoting tissue growth and restoring homeostasis. With aging, changes in monocyte/macrophage phagocytosis and cytokine secretion have been noted,^203^ although there are still inconsistencies among research findings. Research on aging has shown mixed results regarding monocyte behavior: some studies indicate an increase in the production of proinflammatory cytokines by human monocytes as people age, while other studies have observed a decrease in this production.^204^, ^205^, ^206^ Given the vital role of macrophages in governing the proliferation and differentiation of satellite cells, it is reasonable to suggest that any changes in macrophage functions occurring during aging could impair the skeletal muscle's capacity for regeneration.

In summary, excess lipids can overflow and redistribute to various tissues, including skeletal muscles (IMAT and IMCLs). A specific population of skeletal muscle mesenchymal stem cells called FAPs is responsible for the development of IMAT with aging, which may serve as a potential predictor of sarcopenia. IMCLs can potentially lead to mitochondrial dysfunction, impair the β‐oxidation process of fatty acids, and increase ROS production, contributing to lipotoxicity, IR, and inflammation. Proinflammatory factors attract T cells, macrophages, and other immune cells into adipose and muscle tissues. Immunosenescent cells not only directly impede muscle regeneration but also release numerous proinflammatory cytokines and chemokines, inducing local chronic inflammation that extends into a systemic inflammaging state and promotes IR in adipose and muscle tissues. Thus, local inflammation, lipotoxicity, and IR initiate systemic inflammaging, worsening lipid metabolism dysfunction in a perpetuating cycle.

#### 2.6.3 The interaction between adipose tissue and skeletal muscle plays a significant role in the development of sarcopenic obesity

Given the close relationship and interaction between muscle and adipose tissue, it is reasonable to hypothesize that inflamed adipose tissue and inflamed skeletal muscle create a harmful cycle, contributing to age‐related sarcopenia and sarcopenic obesity. Sarcopenic obesity represents a clinical and functional state marked by the simultaneous presence of obesity and sarcopenia.^207^ Indeed, obese adipocytes have the potential to trigger inflammation and muscle cell atrophy, thereby contributing to muscle wasting commonly associated with metabolic disorders.^208^ The consensus is that obesity is typified by a state of low‐level chronic inflammation.^209^ Several immune cell types, such as macrophages, T cells, B cells, and neutrophils, associated with adipose tissue.^210^, ^211^ Growing adipose tissue alters the composition and abundance of both local and systemic immune cells, promoting a transition toward a more proinflammatory profile.^211^ In adipose tissue, this transition involves a shift from anti‐inflammatory M2 macrophages in lean individuals to proinflammatory M1 macrophages in obese individuals.^212^ In obese tissue, a notable feature called “crown‐like” structures can be observed, wherein macrophages surround deceased or dying fat cells.^213^, ^214^ Obese individuals who lack crown‐like structures exhibit improved metabolic control and reduced expression of inflammatory genes.^215^ In obese mice, there is a heightened presence of MCP‐1 in both WAT and plasma.^216^ Indeed, MCP‐1 is recognized as a potent chemotactic factor for monocytes.^217^ Obese mice lacking MCP‐1 exhibited decreased macrophage presence and a reduction in the inflammatory state of adipose tissue. There is an interplay between macrophages and adipocytes, where in obesity, adipocytes release surplus saturated FFA, activating macrophages via the TLR4 signaling pathway. Consequently, macrophages produce TNF‐α, which interacts with tumor necrosis factor receptor 1 on adipocytes, activating the NF‐κB pathway and instigating an inflammatory cascade, resulting in further FFA release.^218^ There are reports proposing that FFA could act on fat cells in an autocrine manner, triggering an inflammatory reaction and elevating adipokine production. This mechanism is thought to occur, in part, via the TLR4 pathway.^219^ Obesity can influence the composition of T‐cell subsets within adipose tissue, where they are thought to play a role in modulating macrophage phenotypes. Thin mice were observed to have elevated levels of CD4+ Tregs and Th2 polarized cells in adipose tissue. These cells contribute to preserving adipose tissue function and insulin sensitivity by promoting an anti‐inflammatory shift in macrophage activation.^220^, ^221^ In obesity, the accumulation of CD8+ effector T cells and CD4+ Th1 cells in adipose tissue can result in the generation of Th1 signals. These signals can initiate the recruitment and activation of macrophages, thus perpetuating the proinflammatory cascade linked to IR.^222^ Thus, the alterations in the signaling equilibrium of Th1‐ and Th2‐types induced by obesity can impact macrophage recruitment and phenotype in adipose tissue, leading to either pathogenesis or environmental protection. Furthermore, B cells have been implicated in obesity‐induced adipose tissue inflammation by promoting T cell and macrophage activation.^223^ Indeed, the secretion of soluble factors by proinflammatory macrophages can reshape tissue composition, fostering a microenvironment conducive to tissue remodeling and reconstruction.

There is evidence suggesting an upregulation of inflammatory cytokine production and heightened inflammation in skeletal muscle among individuals with obesity.^224^ In sarcopenic obesity, adipose tissue‐resident macrophages can foster a sterile inflammatory milieu, potentially dampening insulin signaling. This reinforces the concept of “immune metabolism,” acknowledging macrophages' dual roles—both detrimental and beneficial—in sarcopenic obesity.^98^, ^225^ In obese individuals, muscles exhibit an accumulation of M1 macrophages compared with lean subjects, and this accumulation has been found to correlate with body mass index (BMI). Similarly, studies with mice fed a HFD have reported comparable findings. Furthermore, research indicates that a short‐term high‐fat, high‐calorie diet, or overfeeding, which can induce IR, leads to an increase in macrophage markers in the skeletal muscle of healthy individuals.^226^, ^227^ Histologically, macrophages and T lymphocytes are primarily located in the adipose tissue surrounding skeletal muscle, known as intermyocellular/IMAT. These immune cells are situated between muscle fibers or in close proximity to the muscle.^228^ In obesity, there is a significant increase in the number of macrophages and T cells within these adipose depots (IMAT).^228^ Immune cells in skeletal muscle, akin to those in visceral adipose tissue, also tend to undergo polarization into proinflammatory phenotypes during obesity.^228^, ^229^ Research indicates that in obese mice, there is an increase in both CD4+ and CD8+ T cells within skeletal muscle tissue. Moreover, the proportion of Th1 cells expressing IFN‐γ is elevated, whereas the proportion of Tregs is reduced in the skeletal muscle tissue of mice with obesity.^228^ Macrophages and T lymphocytes are present in skeletal muscle between myofibers, although they occur at a lower frequency compared with the adipose tissue surrounding the muscle.^228^ Indeed, while proinflammatory markers may be more abundant in adipose tissue depots surrounding skeletal muscle (IMAT), infiltrating immune cells can still contribute to the release of proinflammatory molecules and metabolic dysfunction in skeletal muscle. Additionally, under conditions of inflammation or in the presence of inflammatory molecules like FFAs, both adipocytes and myocytes have been demonstrated to secrete increased levels of chemokines,^230^ which induce immune cell migration,^231^, ^232^ including MCP‐1, to recruited infiltration of leukocytes from the circulation into tissues requires. Absolutely, as obesity progresses, recruited immune cells like macrophages and T lymphocytes can indeed secrete chemokines, exacerbating inflammation in both skeletal muscle and adipose tissue, as described. This sets up a feedback loop where the initial inflammatory response triggers the recruitment of more immune cells, which then secrete additional chemokines, amplifying the inflammatory response further. This cycle contributes significantly to the chronic low‐grade inflammation observed in obesity.

Studying changes in fat deposition and distribution during advanced aging is essential, as dysregulated lipid metabolism in adipose tissue can lead to local inflammation and exacerbate the process of inflammaging. These factors are likely contributors to metabolic‐related diseases. The metabolic effects of ectopic fat accumulation present potential therapeutic targets for age‐related conditions, such as sarcopenia. There is evidence supporting the hypothesis that aged adipose tissue serves as a risk factor for sarcopenia, with the bidirectional interaction between adipose tissue and muscle potentially worsening this cycle. However, in older adults, sarcopenic obesity has been associated with better survival outcomes compared with sarcopenia alone.^22^ As the prevalence of sarcopenic obesity increases globally, it is crucial to investigate the underlying mechanisms of this condition.

## 3 PATHOPHYSIOLOGT OF CACHEXIA

The etiology of cachexia involves a complex interplay of mechanisms. A thorough understanding of its pathogenesis is essential for developing targeted prevention and management strategies for this debilitating condition. This section explores the multifaceted etiological factors, cellular and molecular mechanisms, inflammatory processes, and the role of adipose tissue in cachexia.

### 3.1 Systemic inflammation and cytokine dysregulation

Systemic inflammation is a well‐documented feature of cancer cachexia, with increased circulating levels of C‐reactive protein (CRP) associated with weight loss in cancer patients.^233^, ^234^, ^235^ In 1985, Cerami's group^236^, ^237^ demonstrated that circulating mediators could induce cachexia, identifying TNF‐α, initially termed “cachectin.” In cancer cachexia, proinflammatory cytokines produced by immune and tumor cells—particularly TNF‐α, interleukin‐1, ‐6, and ‐8 (IL‐1, IL‐6, IL‐8), and IFNγ—play significant roles in driving the wasting phenotype associated with this syndrome and are classified as procachetic factors.^238^ For instance, serum concentrations of IL‐1 increase in cachectic patients, although its role in tissue wasting remains debated.^239^, ^240^ On one hand, IL‐1 is thought to induce anorexia by increasing tryptophan plasma concentrations, leading to elevated serotonin levels that cause early satiety and suppress appetite.^241^ Inversely, other studies have shown that high circulating IL‐1 does not significantly impact food intake or weight loss, suggesting it may exert local tissue‐specific effects or require high pharmacologic doses to elicit a cachectic response.^242^, ^243^ The role of IFNγ in cachexia is also not fully understood; however, it has been shown to synergize with TNF‐α to promote muscle wasting.^244^, ^245^ IFNγ inhibits myosin mRNA in skeletal muscle cells and activates ubiquitin gene expression.^241^, ^244^ The subsequent sections will delve into the roles of TNF‐α, IL‐6, and IL‐8 in cachexia. Additionally, the decreased expression of anti‐inflammatory cytokines such as IL‐4, IL‐10, and IL‐12 accompanies the upregulation of proinflammatory cytokines, further disrupting the balance between pro‐ and anti‐inflammatory stimuli.^239^ This systemic inflammatory response primarily affects skeletal muscle, indicating that while nonmuscle tissues (such as the host immune system and tumor cells) mainly contribute to skeletal muscle inflammation, increased cytokine production by skeletal muscle itself may also play a role. Therefore, the contribution of skeletal muscle fibers and resident or recruited mononucleated cells in cytokine production warrants further evaluation.

### 3.2 Protein degradation pathways

#### 3.2.1 Activation of the ubiquitin–proteasome system

The ubiquitin–proteasome system (UPS) is a crucial pathway responsible for the degradation of ubiquitinated proteins within cells, playing a significant role in skeletal muscle protein turnover.^246^ Increased UPS activation is particularly important in driving muscle wasting associated with cachexia, as evidenced by numerous studies utilizing animal models of cancer, heart failure, and sepsis to explore the underlying molecular mechanisms.^247^, ^248^, ^249^, ^250^, ^251^

Skeletal muscle is highly susceptible to cachectic factors, such as proinflammatory cytokines, leading to selective degradation of specific muscle proteins rather than a generalized loss. For instance, research involving muscle biopsies from cancer cachexia patients has demonstrated upregulation of ubiquitin mRNA and 20S proteasome subunits, alongside increased proteasome activity compared with healthy controls.^252^, ^253^ Furthermore, in mouse models with colon‐26 tumors, a marked reduction in myosin heavy chain was observed, correlating with muscle wasting.^244^ The enhanced activity of the proteasome pathway in cachexia appears to be mediated by the activation of key transcription factors, including FOXO and NF‐κB, which promote the expression of atrogenes such as MuRF‐1 and MAFbx. These atrogenes contribute to elevated proteasome activity and increased catabolism. Additionally, FOXO transcription factors further exacerbate this catabolic signaling by inhibiting the PI3K/Akt pathway, which is critical for protein synthesis. Importantly, prior to the UPS degrading monomeric actin and myosin, the activation of caspase‐3 through the PI3K pathway is necessary for dissociating actomyosin complexes, thereby facilitating the degradation process. This intricate interplay underscores the complexity of muscle wasting in cachectic conditions.^254^

#### 3.2.2 Involvement of autophagy–lysosomal pathways

The autophagy–lysosome system plays a crucial role in eliminating long‐lived proteins and large supramolecular structures, including dysfunctional mitochondria. During the formation of a double‐membrane structure known as the autophagosome, proteins and organelles destined for degradation are engulfed. The autophagosome then fuses with lysosomes, enabling acidic proteolytic degradation of its contents by cathepsins. There is increasing interest in the role of autophagy in skeletal muscle wasting and the progression of cachexia.^255^, ^256^ Evidence suggests that autophagy is significantly upregulated during cancer cachexia, with elevated levels of mediators such as BNIP3A mRNA and LC3B protein observed in a small cohort of lung cancer patients.^257^, ^258^ In the skeletal muscle of cachectic cancer patients, the protein levels of autophagy‐related genes—such as ATG5, ATG7, Beclin1, and LC3B—are also increased, along with a rise in the number of autophagosomes.^259^, ^260^, ^261^ Additionally, transcription factors like NF‐κB, STAT3, and CCAAT/enhancer‐binding protein‐β (C/EBPβ) contribute to the regulation of E3 ubiquitin ligases and autophagy genes.^262^, ^263^ Since animal models may not fully replicate the complex events of cancer cachexia in humans, it is essential to validate the significance of these transcription factors by assessing their activity in the skeletal muscle of patients with cancer‐associated cachexia.^264^ While autophagy is constantly active in removing damaged proteins and organelles, defects in this process can lead to muscle functional impairment, and excessive autophagy can contribute to muscle mass loss.^265^ Therefore, tight regulation of the autophagy–lysosome system is vital for maintaining skeletal muscle homeostasis. However, in cachexia, the upregulation of autophagy genes results in excessive activation of autophagy pathways, leading to increased breakdown of skeletal muscle.

### 3.3 Metabolic abnormalities and hypercatabolism

One consequence of the systemic metabolic alterations in cancer‐associated cachexia is decreased energy efficiency, primarily due to energy‐wasting mechanisms such as futile metabolic cycles, as demonstrated in preclinical studies.^266^ These changes result in energetic inefficiency, shifting the energy balance toward weight loss through increased resting energy expenditure (REE).^267^ Clinical studies indicate that not all cancer patients exhibit hypermetabolism; however, those with elevated REE experience a higher incidence of treatment‐related toxicities. Furthermore, advanced‐stage cancer patients with high REE have shorter median overall survival durations.^267^, ^268^

Cancer cachexia is often characterized by hypermetabolism, which is frequently accompanied by mitochondrial dysfunction in skeletal muscle, leading to muscle wasting.^269^ For instance, breast cancer patients show dysregulation of pathways governing oxidative phosphorylation, resulting in mitochondrial dysfunction. Additionally, reduced PPAR signaling, which regulates energy metabolism, contributes to this dysfunction by decreasing β‐oxidation. In Lewis lung carcinoma (LLC) mice, tumor progression correlates negatively with mitochondrial ATP synthesis while promoting mitochondrial ROS production.^270^ In patients with gastrointestinal cancer‐associated cachexia, studies have identified disrupted mitochondrial morphology.^259^ Evidence suggests that dysregulated mitochondrial metabolism is critical for muscle wasting in cancer cachexia.^271^ For example, in older gastric cancer patients, muscle loss is associated with diminished mitochondrial protein content and increased mitophagy.^272^

### 3.4 Adipose and muscle wasting

#### 3.4.1 WAT: dysfunctional lipid storage and remodeling

Fat is lost more rapidly than lean tissue in cancer cachexia, highlighting the growing recognition that adipose tissue wasting is a significant component of cancer‐associated weight loss and that fat mass can serve as a predictor of survival in these patients^273^ (Figure 1). Adipose tissue undergoes extensive remodeling during cachexia. In addition to lipid depletion, ECM remodeling occurs, as indicated by transcriptomic profiling of adipose tissue from patients with gastrointestinal cancer.^274^ Enhanced collagen accumulation and fibrosis have also been observed in adipose tissue specimens from patients experiencing cancer cachexia.^275^, ^276^ This may contribute to increased recruitment of inflammatory cells into adipose tissue, causing local inflammation and potentially exacerbating the systemic inflammation commonly seen in cachexia.^277^

Even more strikingly, transcriptomic profiling of adipose tissue from gastrointestinal cancer patients suggests that both energy turnover and fatty acid degradation are significantly elevated in individuals with cachexia.^274^ Specifically, lipolysis— the hydrolysis of TGs into FFAs and glycerol— is linked to this wasting syndrome, with increased plasma levels of FFAs and glycerol often noted in cachectic patients.^278^ Lipolysis and palmitate oxidation are heightened in weight‐losing cancer patients,^279^ and elevated lipolysis in those with gastrointestinal adenocarcinoma correlates with increased expression of hormone‐sensitive lipase, a key lipolytic enzyme, in adipose tissue.^280^ Indeed, the activities of both hormone‐sensitive lipase and adipocyte TG lipase are elevated in the adipose tissue of cachectic patients, and knockout studies in mice have confirmed the central role of these lipases in the progression of cachexia.^281^ A futile substrate cycle between lipolysis and lipogenesis further contributes to the negative energy balance observed in murine models of cachexia.^16^, ^282^

Dysfunction of adipose tissue in cachexia can have additional systemic effects. The infiltration of adipose tissue cells into skeletal muscle appears to contribute to muscle wasting. One study reported a correlation between the increased presence of IMCLs—indicative of adipose tissue cell infiltration in muscle—and body weight loss in patients with cancer.^283^ Insufficient lipid storage can lead to lipotoxicity, particularly detrimental effects arising from lipid accumulation in muscle, heart, and liver. Altered adipose tissue function may also result in changes to the secretion of lipokines and reactive lipid metabolites (such as lysophosphatidic acid, ceramide, and DAG), as well as adipokines (such as adiponectin and leptin). These alterations can contribute to local or systemic inflammation, ultimately leading to metabolic dysfunction.^284^, ^285^ Although research in these areas is still in its early stages, the potential for developing multitargeting therapies centered on adipose tissue in future cachexia treatment remains promising.

#### 3.4.2 BAT: brown and cachexia

Recent studies have highlighted a different aspect of adipose tissue biology in the progression of cachexia. Evidence shows enhanced BAT activity in cachectic mice, indicating a shift from energy storage to energy expenditure.^286^ Research led by Petruzzelli et al. demonstrated that the browning of sWAT occurs in the early stages of cachexia, prior to muscle wasting.^245^ This phenotypic transformation involves the conversion of white adipocytes to beige fat, characterized by increased UCP1 content and elevated expression of browning markers such as PGC‐1α, Cidea, and Prdm16 across multiple mouse models of cachexia (including syngeneic, genetically engineered, and xenograft human tumors). Increased energy expenditure was observed in cachectic mice, along with higher mitochondrial content and oxygen consumption in sWAT.^245^ Notably, inflammatory signaling was present in sWAT from these mice. Treatments using neutralizing antibodies targeting IL‐6, IL‐6 receptor‐deficient mice, and the anti‐inflammatory drug sulindac were effective in ameliorating fat depletion and reducing UCP1 expression. Additionally, increased expression of thermogenic markers UCP1 and PGC‐1α was noted in BAT in response to central delivery of TNF‐α.^287^, ^288^ The study also identified that β‐adrenergic signaling, typically known for inducing beige fat in cold conditions, plays a role in cancer cachexia development. While treatment with a β3‐AR antagonist improved cachexia and reduced UCP1 levels, nonsteroidal anti‐inflammatory treatments were generally more effective.^245^ Beyond proinflammatory cytokines, other molecules, such as parathyroid hormone‐related peptide (PTHrP), have been implicated in cancer cachexia progression. PTHrP, secreted by LLC, can induce markers of WAT browning, thermogenesis, and energy metabolism in primary adipocytes and in vivo.^289^ Treatment of cachectic LLC mice with a PTHrP neutralizing antibody preserved body weight and adipose tissue, partially protecting muscle from wasting. Inhibition of PTHrP blocked increases in UCP1, PGC‐1α, Dio2, and Atgl expression in sWAT, normalizing energy expenditure in LLC tumor‐bearing mice. Although the metabolic function of PTHrP is not fully understood, it is expressed in human WAT and may be associated with IR.^289^, ^290^ In vitro studies indicate that PTHrP stimulates cellular respiration through PKA signaling, suggesting potential interactions with the β‐adrenergic pathway.^291^, ^292^

Overall, these findings reveal the detrimental metabolic effects of various cachectic mediators on adipose tissue function. Targeting the activation of BAT and the browning of WAT presents promising pharmacological strategies to maintain energy balance and alleviate cachexia in cancer patients.

### 3.5 Tumor and disease‐specific factors (in cancer cachexia)

Tumors can produce factors that induce anorexia, exacerbating the impact of direct muscle‐acting factors. Borner and colleagues^293^ described a rat hepatoma model where increasing tumor burden led to progressive anorexia, resulting in significant weight loss and muscle wasting. This process is associated with elevated levels of macrophage inhibitory cytokine‐1, also known as growth differentiation factor 15.^293^

Hogan et al.^294^ reported that lung cancer cell lines secrete various factors that inhibit myoblast differentiation, including IGFBP‐3, C‐X‐C motif chemokine ligand 1 (CXCL1), and C‐C motif chemokine ligand 2 (CCL2). Other studies have confirmed the tumor‐derived secretion of CXCL1, which has multiple effects within the tumor microenvironment.^295^, ^296^ Additionally, leukemia inhibitory factor (LIF) has been identified as another significant tumor‐derived cytokine.^297^ In a murine model of C26 colon carcinoma, LIF was shown to induce atrophy in myotubules, leading to reductions in muscle mass and myofiber size, while fat mass remained unaffected.^298^

Much research has focused on the tumor's ability to promote inflammation, a primary contributor to cancer‐induced muscle wasting.^299^, ^300^ The question arises as to whether specific tumor characteristics trigger the release of proinflammatory mediators. Scientists demonstrated that in various cancers—including breast, head and neck, lung, colorectal, and stomach—there is a positive correlation between the expression of proinflammatory cytokines (such as IL‐1α, IL‐1β, IL‐6, IL‐8, and TNF‐α) and Fn14, the receptor for TWEAK.^2^, ^301^, ^302^ In mice with Fn14‐expressing tumors, targeting Fn14 with antibodies significantly mitigated tumor‐induced weight loss and extended lifespan. Several human tumor cell lines also secrete activin A and myostatin.^303^, ^304^ Activin A plays a crucial role in stimulating an inflammatory response at the tumor level. In patients with lung and colorectal cancers, activin A levels correlate with inflammatory markers like CRP,^305^ which is known to associate with IL‐6—a procachectic cytokine produced by some tumor cells.^306^, ^307^, ^308^

Last, as previously summarized, in a LLC model of cancer cachexia, tumor‐derived PTHrP has been shown to drive thermogenesis (“browning”) in adipose tissue.^289^, ^302^ While PTHrP does not directly affect muscle mass, it significantly exacerbates skeletal muscle wasting and dysfunction in the presence of tumors.

## 4 MOLECULAR MECHANISMS

Sarcopenia and cachexia are recognized as multifactorial syndromes that share overlapping mechanisms. While most individuals experiencing cachexia are also sarcopenic, the majority of those with sarcopenia do not meet the criteria for cachexia, indicating that sarcopenia may be viewed as a component of cachexia.^10^, ^13^, ^14^ In this discussion, we will explore the common pathways that link sarcopenia and cachexia, as well as the bidirectional relationship between adipose tissue and muscle, which serves as a molecular mechanism for both conditions.

### 4.1 Common pathways in sarcopenia and cachexia

#### 4.1.1 Myostatin and activin signaling

Myostatin is a key pathway involved in muscle atrophy, particularly in certain cachexia models and sarcopenia. As a member of the TGF‐β family, myostatin is secreted by muscle cells and circulates in the bloodstream.^309^, ^310^ It negatively regulates skeletal muscle growth, as evidenced by the significantly larger muscle size observed in myostatin knockout mice compared with wild‐type animals, which exhibit hyperplastic and hypertrophic muscle cell activation.^311^

Myostatin and activin A share the same receptor, activin type 2 receptor B (ActR2B).^312^ Research has shown that the expression of a dominant‐negative ActR2B in transgenic mice results in skeletal muscle hypertrophy.^313^ Both myostatin and activin A upregulate FOXO expression, leading to protein breakdown through MuRF1 and MAFbx/Atrogin1, while inhibiting protein synthesis by repressing the Akt/mTOR signaling pathway via SMAD3 activation.^309^, ^313^, ^314^, ^315^ In skeletal muscle, the binding of myostatin and activin A to ActR2B activates the transcription factors SMAD2 and SMAD3, resulting in the expression of atrogin‐1.^12^, ^251^

Circulating levels of activin A are consistently elevated in cachectic cancer patients and in cachectic cancer mice.^305^, ^316^, ^317^, ^318^, ^319^ However, studies indicate that the mRNA levels of activin A in skeletal muscle are either decreased or unchanged, while protein levels are increased.^314^, ^316^, ^320^, ^321^ This suggests that circulating activin A may originate from sources other than skeletal muscle, particularly tumor cells.^303^, ^319^ Conversely, while myostatin signaling is commonly activated during cancer cachexia in rodents, it does not appear to be activated in the skeletal muscle of cachectic cancer patients. However, this conclusion must be nuanced, as investigations have shown that genes associated with TGF‐β signaling are upregulated in the skeletal muscle of cachectic cancer patients, and the transcript levels of ActRIIB negatively correlate with muscle mass in these individuals.^322^, ^323^ One possible explanation for these findings is that myostatin, produced by skeletal muscle, may have decreased circulating levels due to reductions in muscle mass during cancer cachexia. Additionally, strict temporal regulation of myostatin expression has been demonstrated in mouse models of atrophy, indicating that myostatin levels may have increased earlier in the disease process, before significant muscle loss occurred.

In conclusion, targeting myostatin and activin A presents a promising avenue for developing therapies to prevent muscle wasting associated with cachexia and sarcopenia. Nonetheless, further investigation into the roles of myostatin and activin A in muscle physiology is essential for understanding their full implications.

#### 4.1.2 NF‐κB and STAT3 pathways in inflammation

Both the proinflammatory pathways of NF‐κB and STAT3 are implicated in sarcopenia and cachexia. The NF‐κB transcription factor transmits signals from various cytokines, primarily IL‐1β and TNF‐α. For instance, in aging mouse models, the activation of NF‐κB signaling reduces the number of MuSCs, hindering muscle repair and increasing inflammatory gene expression.^324^ Inhibiting NF‐κB can enhance MyoD expression, a key regulator of myogenesis, while also suppressing proinflammatory mediators and facilitating muscle cell differentiation.^325^, ^326^ In the context of cancer cachexia, NF‐κB inhibits MyoD expression at the transcriptional level following TNF‐α activation.^262^, ^327^ Notably, clinical studies have revealed elevated NF‐κB levels in patients with cancer cachexia, particularly those with advanced non‐small‐cell lung cancer, compared with healthy individuals.^328^

STAT3 plays an essential role in sarcopenia driven by various factors, including aging, physical inactivity, poor nutrition, chronic diseases, hormonal changes, inflammation, oxidative stress, neurodegenerative processes, genetics, and medications. However, opinions on STAT3's role in muscle biology are conflicting. Some studies suggest that STAT3 promotes muscle regeneration by activating satellite cells and enhancing mitochondrial function in skeletal MuSCs.^329^ Conversely, other research indicates that activation of the JAK2/STAT3 pathway, mediated by IL‐6, can lead to muscle atrophy, with STAT3 inhibition mitigating muscle wasting caused by cancer cachexia.^263^ In cachectic cancer mice, both STAT3 activation and the expression of its target genes are elevated alongside increased IL‐6 levels.^330^, ^331^, ^332^ Inhibiting STAT3 using a mutated construct to induce dominant negative activity can partially reverse skeletal muscle wasting downstream of IL‐6 by inhibiting the UPS, both in vitro and in vivo.^333^ Transcriptomic analyses also highlight STAT3's involvement in muscle atrophy during cancer cachexia. Furthermore, excessive STAT3 activation in cancer models exacerbates weight and muscle mass loss compared with controls.^265^

These findings suggest that there is an optimal range of STAT3 expression; both excessively high and low levels can disrupt muscle function. This duality implies that STAT3 may exert different effects depending on the muscle's condition. Under stable conditions, STAT3 serves as a vital signaling molecule involved in muscle synthesis and repair. However, during unstable states—such as chronic inflammation or IR—excessive STAT3 activation can contribute to muscle atrophy.^334^

#### 4.1.3 IGF‐1/AKT/mTOR pathway and its role in muscle maintenance

As we have summarized above, IGF1/AKT/mTOR pathway is positively regulate muscle synesis. IGF‐1 participates in a variety of cellular functional activities. IGF‐1 and its related receptors and pathways have also become therapeutic targets for aging and metabolic diseases.^335^ For example, IGF‐1 has been shown to play a physiological role in muscle by regulating skeletal muscle proteostasis and hypertrophy as well as protecting against age‐related muscle mass loss.^336^ Low IGF‐1 secretion is related to poor muscle mass, muscle strength and slow walking speed.^337^ It has been reported that the age‐induced decrease in IGF‐1 secretion is associated with sarcopenia, muscle frailty, and upper extremity obesity.^338^, ^339^, ^340^ Furthermore, PI3K/Akt activation prevents protein degradation in skeletal muscle by phosphorylating and inactivating FOXO transcription factors. mTOR is also a cellular energy sensing molecule that can sense the energy state and regulate its downstream signaling pathway, thereby regulating cell metabolism, cell cycle processes and cell growth.^341^ mTOR signaling plays a fundamental role in adipogenesis, interacting with several proteins to form two distinct complexes named mTORC‐1 and mTORC‐2 reviewed previously,^342^ which has become a new target for the treatment of aging diseases.^343^ Furthermore, mTORC1 activation leads to severe muscle atrophy and low body mass,^344^, ^345^ blocking mTORC1 signaling, inhibiting adipogenesis and impairing the maintenance of fat cells.^346^, ^347^ Blocking mTOR signaling might extend life span and prevent the onset of age‐related diseases in mammals (e.g., sarcopenia), suggesting that mTOR inhibitors exert beneficial effects on human longevity.^345^ Agarwal reported that inhibition of the mTOR pathway could alleviate muscle fibrosis and improve myo‐fiber regeneration.^348^ Specifically, the mTOR pathway participates in skeletal muscle generation and muscle protein synthesis by regulating 4E‐BP1 and S6K1.^349^ Overall, impairment in anabolic signaling in sarcopenia, as evidenced by suppressed IGF‐1/AKT/mTOR pathway.

Similarly, global transcriptomic and biochemical analyses indicate that the activity of the insulin/IGF‐1–AKT–mTOR pathway is diminished in the cachectic muscle of cancer patients and animal models. For instance, AKT protein levels, along with the phosphorylation of GSK3, mTOR, and S6K, are decreased in the skeletal muscle of cachectic cancer patients compared with their noncachectic counterparts. This is also reflected in the phosphorylated inactive form of the translational repressor 4EBP1.^328^, ^350^ These changes are associated with weight loss,^351^ suggesting that greater inhibition of the pathway correlates with more severe cachexia. While some discrepancies have been noted between studies in humans and animals,^260^, ^352^ impairment of the IGF‐1/AKT/mTOR pathway may be a risk factor for muscle dysfunction in cachexia.

### 4.2 Role of mitochondrial dysfunction in muscle wasting

Mitochondria within skeletal muscle are incredibly dynamic organelles that demonstrate substantial adaptability in their composition, structure, metabolism, and role, responding to diverse stresses such as exercise, muscle disuse, aging, cachexia, and sarcopenia.^353^, ^354^, ^355^ Mitochondrial decay is one of the main factors that promotes muscle aging.^356^ Research has shown that the mitochondria of both mice and humans during aging may undergo various changes, such as reduced mitochondrial protein synthesis, mitochondrial enlargement, impairment of mitochondrial permeability transition pore function, alterations in mitochondrial enzyme concentrations, greater generation of ROS, and increased mtDNA mutations.^5^ PGC‐1α is known to be involved in physiological processes, including mitochondrial biogenesis, thermogenesis, glucose homeostasis, and oxidative stress responses.^357^, ^358^, ^359^ During aging, mitochondrial biogenesis is decreased in muscle cells, driven by a reduction in PGC‐1α expression and acetylation.^360^, ^361^ Apoptosis mediated by mitochondria has been shown to contribute to the pathogenesis of sarcopenia due to the vital role of mitochondria in apoptotic signaling integration and the decline in mitochondrial function in skeletal muscle with age.^356^

Mitochondrial abnormalities resulting from changes in biogenesis and dynamics are significant in cachexia models.^362^ For instance, FIS1, a protein that regulates mitochondrial morphology, is elevated in the skeletal muscle of patients with cancer‐associated cachexia.^259^ In mouse models of this condition, key indicators of mitochondrial dynamics—such as mitofusin 2, optic atrophy 1 protein, FIS1, and density‐regulated protein—are downregulated in skeletal muscle, indicating compromised mitochondrial function.^363^, ^364^ Additionally, mitophagy, the process of removing dysfunctional mitochondria, appears to be heightened in skeletal muscle during cancer‐associated cachexia. This is evidenced by increased levels of mitophagy markers like BNIP3 and microtubule‐associated protein 1B, which are linked to mitochondrial dysfunction and apoptosis.^365^ These mitochondrial alterations contribute to impaired respiration in skeletal muscle,^365^, ^366^, ^367^ leading to enhanced production of ROS and oxidative stress, which further exacerbate mitochondrial dysfunction.

### 4.3 Molecules play a role in sarcopenia and cachexia by mediating the crosstalk between adipose and skeletal muscle

**FIGURE 2:**
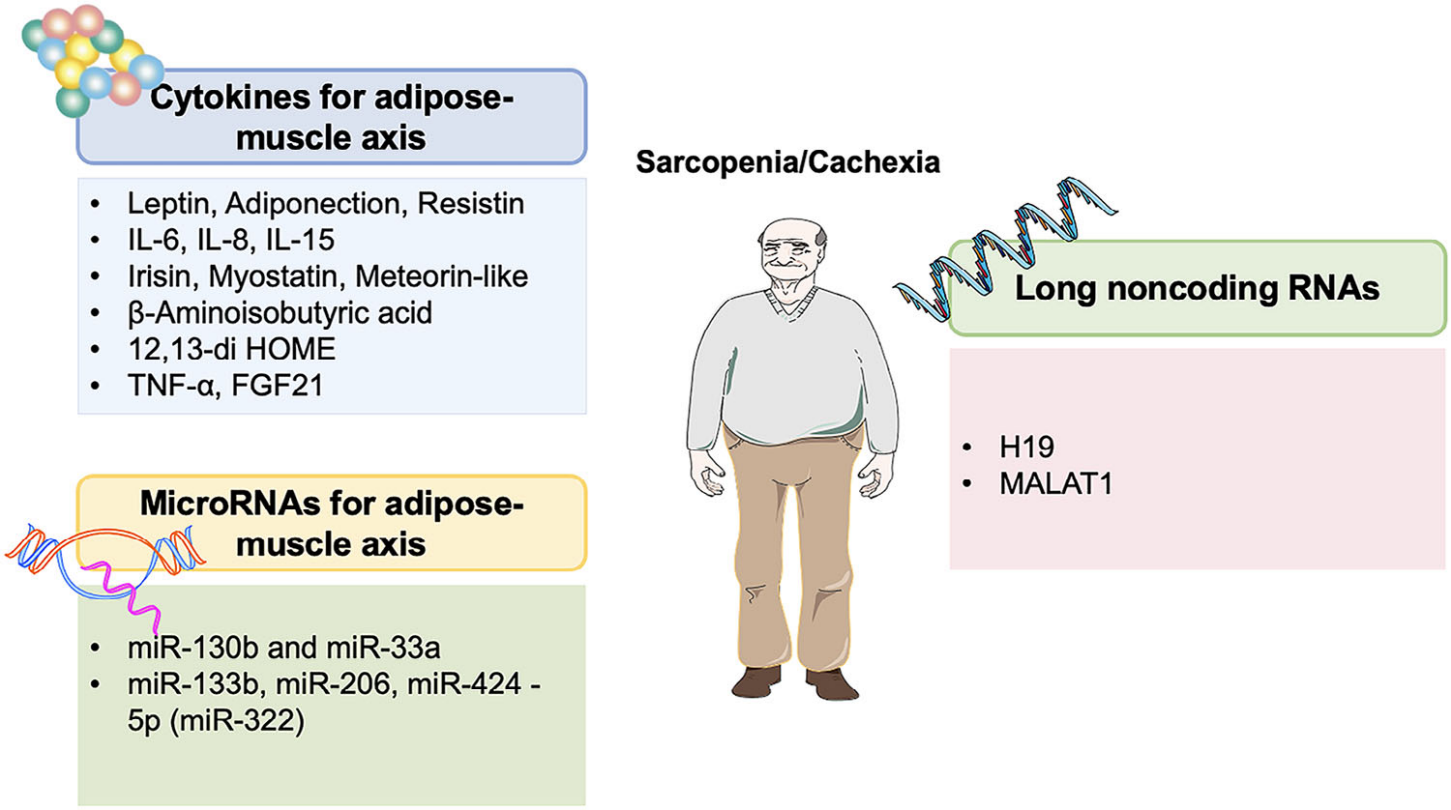
Molecules influence sarcopenia and cachexia by mediating the interaction between adipose tissue and skeletal muscle. The figure highlights the cytokines, miRNAs, and lncRNAs potentially involved in sarcopenia and cachexia by regulating the adipose–muscle axis.

As summarized, there is significant crosstalk between adipose tissue and skeletal muscle in the pathogenesis of both sarcopenia and cachexia. Both tissues function as endocrine organs, releasing adipokines and myokines that facilitate a wide range of interorgan communication.^368^, ^369^, ^370^ This communication can occur through direct cell–cell contact via gap junctions or through the release of extracellular vesicles (EVs) into neighboring tissues or the circulatory system. EVs, including exosomes and microvesicles, are cell‐derived particles that play a crucial role in regulating both local and distant target cells. They carry a diverse array of cargo, such as cytokines, proteins, lipids, organelle components, and noncoding RNAs, including miRNAs and other small regulatory RNAs.^371^, ^372^ Exosomes derived from adipose and skeletal muscle tissues can exert reciprocal regulatory functions.^373^, ^374^ Aging and cancer induce changes in cytokines and noncoding RNAs, leading to unfavorable fat deposition and accumulation, persistent low‐grade inflammation, and impairments in lipolysis, glucose uptake, and insulin sensitivity. These alterations contribute to the pathophysiology of sarcopenia and cachexia (see Tables 1 and 2). Below, we will provide a conceptual framework to explain how adipose tissue communicates with skeletal muscle to influence the development of sarcopenia and cachexia (Figure 2).

**TABLE 1:** Cytokines bridge the crosstalk between muscle and adipose.

Potential molecules	Suggested role in muscle	Suggested role in adipose	References
Leptin	Leptin stimulates myocyte proliferation, increases myogenin and myonectin transcript levels, increases basal glucose uptake in isolated soleus muscles, and reduces mRNA expression of muscle atrophy‐related factors.	Leptin blunts insulin response, decreases insulin‐induced glucose uptake and lipogenesis in white adipocytes. Leptin stimulates glucose uptake by brown fat tissue, raises brown fat tissue temperature.	^375^, ^376^, ^377^, ^378^, ^379^, ^380^, ^381^, ^382^, ^383^, ^384^, ^385^, ^386^, ^387^, ^388^, ^389^, ^390^, ^391^, ^392^, ^393^, ^394^, ^395^, ^396^, ^397^, ^398^, ^399^, ^400^, ^401^, ^402^, ^403^, ^404^, ^405^, ^406^, ^407^, ^408^, ^409^
Adiponectin	Adiponectin promotes the oxidation of fatty acids and glucose uptake, participates in the regulation of glucose and lipid metabolism, and regulates the energy homeostasis of organisms.	Adiponectin decreases muscular lipid content, reduces IR, has profound effects on converting white fat tissue to brown fat tissue, improves metabolism.	^410^, ^411^, ^412^, ^413^, ^414^, ^415^, ^416^, ^417^, ^418^, ^419^, ^420^, ^421^, ^422^, ^423^, ^424^, ^425^, ^426^, ^427^, ^428^, ^429^, ^430^, ^431^, ^432^, ^433^, ^434^
Resistin	Resistin decreases fatty acid uptake and metabolism, lowers glucose uptake, inhibits myogenic differentiation.	Unknown	^435^, ^436^, ^437^, ^438^, ^439^, ^440^, ^441^, ^442^, ^443^, ^444^, ^445^, ^446^, ^447^, ^448^, ^449^
IL‐6	IL‐6 has dual roles in regulating the homeostasis of muscle. Muscle derived IL‐6 enhances glucose uptake and fatty acid oxidation in myotubes. Adipose tissue‐released IL‐6 might contribute to some metabolic diseases, along with chronic inflammation, inducing muscle dysfunction.	Whit fat tissue conversion into beige adipose tissue was indirectly affected by muscle‐secreted IL‐6. Visceral fat can release large amounts of IL‐6 and is more correlated with diabetes than total body fat.	^450^, ^451^, ^452^, ^453^, ^454^, ^455^, ^456^, ^457^, ^458^, ^459^, ^460^, ^461^, ^462^, ^463^, ^464^, ^465^, ^466^, ^467^, ^468^, ^469^, ^470^, ^471^, ^472^, ^473^, ^474^, ^475^, ^476^, ^477^, ^478^, ^479^, ^480^, ^481^, ^482^, ^483^, ^484^, ^485^, ^486^, ^487^
IL‐8	IL‐8 creates a microenvironment that limits glucose disposal, which might be negatively correlated with muscle mass.	Both higher levels of IL‐8 in plasma and VAT are associated with IR, contributing to T2DM and obesity.	^488^, ^489^, ^490^, ^491^, ^492^, ^493^, ^494^, ^495^, ^496^, ^497^, ^498^, ^499^, ^500^, ^501^, ^502^, ^503^, ^504^, ^505^, ^506^, ^507^, ^508^
IL‐15	IL‐15 increases myotube development and induces myoblast differentiation (hypertrophic effects). IL‐15 might improve oxidative characteristics and glucose homeostasis.	IL‐15 induces adipose tissue catabolism and decrease adiposity.	^509^, ^510^, ^511^, ^512^, ^513^, ^514^, ^515^, ^516^, ^517^, ^518^, ^519^, ^520^, ^521^, ^522^, ^523^, ^524^, ^525^
Irisin	Irisin increases glucose utilization and reduces epididymal fat mass in skeletal muscle through increasing mitochondrial content and oxygen consumption.	Irisin promotes UCP1 expression in adipocytes, causing white fat tissue to adopt a brown fat tissue‐like phenotype by increasing energy expenditure.	^526^, ^527^, ^528^, ^529^, ^530^, ^531^, ^532^, ^533^, ^534^, ^535^, ^536^, ^537^, ^538^, ^539^, ^540^, ^541^, ^542^, ^543^, ^544^, ^545^, ^546^
Myostatin	Myostatin stimulates protein degradation, inhibits protein synthesis, resulting in skeletal muscle wasting.	Myostatin is associated with the thermogenic capacity of BAT and the browning of WAT. A negative feedback loop of BAT activity has been reported.	^547^, ^548^, ^549^, ^550^, ^551^, ^552^, ^553^, ^554^, ^555^, ^556^, ^557^, ^558^, ^559^, ^560^, ^561^, ^562^, ^563^, ^564^, ^565^, ^566^, ^567^, ^568^, ^569^, ^570^, ^571^, ^572^, ^573^
Meteorin‐like	Metrnl improves insulin sensitivity in differentiated C2C12 cells and soleus skeletal muscle.	Metrnl upregulated the expression of genes associated with the browning of white fat tissue. Adipocyte Metrnl antagonizes IR, improves adipose function, including adipocyte differentiation, metabolism activation, and inflammation inhibition.	^574^, ^575^, ^576^, ^577^, ^578^, ^579^, ^580^, ^581^, ^582^, ^583^, ^584^
β‐Aminoisobutyric acid	BAIBA upregulates sensitivity and fatty acid oxidation in muscle.	BAIBA upregulates brown adipocyte‐like gene expression signature as well as elevated mitochondrial activity.	^585^, ^586^, ^587^, ^588^
12,13‐di HOME	12,13‐diHOME can increase skeletal muscle fatty acid uptake and oxidation. Stimulating fatty acids into the working skeletal muscle.	Injection of 12,13‐diHOME facilitates BAT thermogenesis by selectively promoting fatty acid uptake, providing fuel for BAT. Improving metabolic changes in response to exercise.	^589^, ^590^, ^591^, ^592^, ^593^
TNF‐α	TNF‐α has dual roles in regulating the homeostasis of muscle. Long‐term increase in TNF‐α secreted from immune cells damages muscle. TNF‐α from myofibrils in the early stage of muscle injury improve the repair of muscle.	TNF‐α promotes adipocytes apoptosis, inhibits the differentiation of precursor adipocytes, and weakens insulin signal.	^594^, ^595^, ^596^, ^597^, ^598^, ^599^, ^600^, ^601^, ^602^, ^603^, ^604^, ^605^, ^606^, ^607^, ^608^, ^609^, ^610^, ^611^, ^612^, ^613^, ^614^, ^615^
FGF21	FGF21 counteracts muscle stress, involves in protecting against diet‐induced obesity and IR.	FGF21 has endocrine effects leading to increased browning of WAT, with upregulation of the UCP1 gene and PGC‐1α protein.	^616^, ^617^, ^618^, ^619^, ^620^, ^621^, ^622^, ^623^, ^624^, ^625^, ^626^, ^627^, ^628^, ^629^, ^630^, ^631^, ^632^, ^633^

**TABLE 2:** Noncoding RNAs bridge the crosstalk fat mass and muscle.

Potential molecules	Suggested role in muscle	Suggested role in adipose	References
miR130b	miR130b decreases the expression of PGC‐1α	The expression of miR‐130b in WAT increases with obesity. MiR130b level is positively correlated with BMI and predictive of the metabolic syndrome	^634^
miR‐33a	miR‐33a impairs myoblasts proliferation	Mice deficient in miR‐33a became obese with increased adipose tissue expansion, increased lipid uptake, and impaired lipolysis and IR.	^635^, ^636^, ^637^
miR‐133	Controversial. miR‐133 stimulates myoblast proliferation while hindering myoblast differentiation; miR‐133 promotes myogenic differentiation.	Unknown	^638^, ^639^, ^640^, ^641^, ^642^, ^643^, ^644^, ^645^, ^646^
miR‐206	miR‐206 downregulates Notch3 and allow myoblast differentiation, regulates myogenesis by inhibiting connexin 43 expression, participates in skeletal muscle growth and regeneration by repressing the expression of Pax7.	miR‐206 decreases adipocytes proliferation and triglyceride accumulation.	^647^, ^648^, ^649^, ^650^, ^651^, ^652^, ^653^, ^654^, ^655^, ^656^, ^657^, ^658^
miR‐424 ‐5p (miR‐322)	miR‐424 ‐5p inhibits protein synthesis in muscle cells, causes fiber atrophy, plays an important role in muscle wasting.	Unknown	^659^, ^660^, ^661^, ^662^, ^663^
H19	H19 facilitates muscle regeneration, improves glucose metabolism. Deficiency of *H19* impairs muscle differentiation.	Unknown	^664^, ^665^, ^666^, ^667^, ^668^, ^669^, ^670^, ^671^
MALAT1	MALAT1 is involved in the proliferation and differentiation of skeletal cells	Knocking down MALAT1 significantly inhibited the process of adipogenesis	^672^, ^673^, ^674^, ^675^, ^676^, ^677^

#### 4.3.1 Dysregulation of cytokines

##### Leptin

Leptin is an adipokine produced and secreted by WAT and BAT, the periosteum, the placenta, and other tissues, and regulates both muscle and adipose tissue. Leptin has a significant role in maintaining energy balance and metabolism. Leptin can also regulate most peripheral organs, such as adipose tissue and muscle.^375^

Although adipocytes are a main source of leptin, they also express leptin receptor (LepR),^376^, ^377^ which is an important target of leptin. Leptin decreases insulin‐induced glucose uptake and lipogenesis in white adipocytes by blunting the insulin response.^378^, ^379^ Moreover, leptin also has a thermogenic effect, promoting energy expenditure in rodents.^380^, ^381^ Whether leptin can also affect BAT development in humans remains to be determined.

The relationship between skeletal muscle and leptin is complex. On the one hand, leptin exerts beneficial effects on muscle, as reduced skeletal muscle atrophy and mass in leptin‐deficient mice (ob/ob) mice can be corrected by intraperitoneal administration of leptin.^382^ Leptin administration stimulates the proliferation of myocytes, increases myonectin and myogenin transcript levels, and reduces the mRNA levels of cytokines related to muscle atrophy in ob/ob mice, such as MAFbx and MuRF1.^383^ Leptin promotes C2C12 myoblast proliferation but impairs myogenesis in C2C12 cells through JAK/STAT and MEK signaling pathways.^384^ A minimal increase in the number of self‐renewed MuSCs was observed in LepR‐deficient (db/db) mice.^385^ Moreover, leptin facilitates metabolism in skeletal muscle. Skeletal muscle cells continuously treated with leptin showed enhanced fatty acid uptake due to activation of AMPK in the short term and the STAT3 pathway in the long term.^386^, ^387^ Dulloo et al.^388^ found that leptin stimulated skeletal muscle thermogenesis by regulating PI3K activity. Moreover, leptin treatment increased basal glucose uptake in isolated soleus muscles from rats and mice, as well as in skeletal muscle cells.^389^, ^390^, ^391^ Collectively, these data provide important evidence that leptin treatment antagonizes lipogenic effects on skeletal muscle and promotes glucose disposal. However, some studies showed that plasma leptin levels are higher in older sarcopenia subjects and obese individuals^392^, ^393^, ^394^ and that circulating leptin is elevated during aging even in the absence of obesity.^395^ A prospective cross‐sectional cohort study showed that the circulating level of leptin was significantly elevated in the sarcopenia group and correlated with sarcopenia incidence, sarcopenia severity, and sarcopenia risk.^393^ A longitudinal study of a cohort of community‐dwelling older adults showed that serum leptin concentrations were positively associated with incident frailty.^394^ Kohara et al.^392^ found that plasma leptin levels are highest in males and females who are both sarcopenic and overweight compared with subjects with only one abnormality. Research has shown that patients with sarcopenic obesity have higher serum leptin levels than those with nonsarcopenic obesity. In addition, they show poor grip strength and physical function. OA patients with sarcopenic obesity had significantly higher serum leptin levels than those with nonsarcopenic obesity. In addition, knee OA patients with sarcopenic obesity displayed low grip strength and poor physical performance.^396^

According to this discrepancy, we believe that there is an optimal concentration of leptin for muscle homeostasis. Both aging and obesity increase serum leptin levels, and beyond this optimal concentration, leptin exerts a destructive effect, while obesity may amplify these detrimental effects. This is supported by the finding that a high level of leptin contributes to central leptin resistance and IR and impairs leptin signal transduction in muscle cells, including lipid and carbohydrate metabolism and fatty acid oxidation.^397^, ^398^ High levels of leptin were proven to impede intramuscular fatty acid oxidation and lipid metabolism, thereby leading to skeletal muscle dysfunction.^98^, ^399^ Impaired fatty acid oxidation and intramyocellular lipid accumulation may promote lipotoxicity.^400^, ^401^ In light of these studies, it appears that leptin may be involved in the pathogenesis of sarcopenic obesity. Thus, it may result in a decline in muscle quality and increased intramuscular fat infiltration.^402^, ^403^ Moreover, both leptin and IR of myocytes are associated with metabolic disorders, including obesity and the type 2 diabetes mellitus (T2DM), creating a vicious cycle augmenting muscle dysfunction.^404^

Although the role of leptin in cancer cachexia is controversial, it is believed to participate in its pathogenesis. In cancer cachexia, leptin is produced by adipose tissue and acts on the hypothalamus to regulate energy storage by influencing appetite.^405^ Additionally, leptin has been shown to upregulate the production of inflammatory cytokines.^406^ Interestingly, leptin‐binding proteins, such as CRP, can bind to leptin in the bloodstream, limiting its binding to the LepR and affecting its localization across the blood‐brain barrier.^407^ Studies involving ob/ob have demonstrated that this adipokine is crucial for controlling body weight and lipid metabolism.^408^, ^409^ Furthermore, ob/ob mice develop obesity due to a lack of appetite suppression.^408^, ^409^ Physiologically, leptin is thought to protect adipocytes from lipotoxicity by inhibiting lipogenesis and increasing fatty acid oxidation. Overall, these studies underscore the role of leptin in body weight regulation.

In summary, leptin is involved in the adipose tissue–muscle axis, implicated in the pathogenesis of sarcopenia and cachexia. Further studies are needed to identify the optimal concentrations of leptin to guarantee muscle and whole‐body homeostasis.

##### Adiponectin

Adiponectin, an adipokine secreted by WAT and BAT,^410^, ^411^ plays a critical role in multiple highly‐specific biological functions and the insulin signaling pathway. It produces a variety of physiological effects by binding to adiponectin receptors (AdipoRs) on the target cell membrane.

Adiponectin has recently received a great deal of attention for its beneficial effects on muscle. Knockout of adiponectin abrogates the exercise‐induced increase in muscle mass and strength in mice, indicating it plays a role in hypertrophy.^412^ C2C12 myoblast cells treated with either globular adiponectin or a mimetic of globular adiponectin (GTDF) displayed a significant increase in both differentiation and fusion indices with respect to vehicle‐treated cells through downregulating key genes associated with muscle atrophy (MuRF1 and atrogin‐1).^413^ Adiponectin also regulates energy homeostasis.^414^, ^415^, ^416^, ^417^ Yoon et al.^415^ treated differentiated C2C12 cells with purified adiponectin and found increased fatty acid β‐oxidation in muscle by sequential activation of AMPK, via the p38 MAPK pathway and PPARα. Ceddia et al.^418^ treated L6 rat skeletal muscle cells with GTDF and showed increased glucose uptake by inducing the translocation of GLUT4 to the plasma membrane. In addition, it was demonstrated that muscle differentiation requires autophagy driven by adiponectin, promoting myoblast survival while inhibiting apoptosis.^419^ Likewise, adiponectin knockout mice showed decreases in autophagy‐related genes (e.g., LC3 and beclin‐I) in skeletal muscle, accompanied by a myopathic phenotype.^419^

In fact, the activity of adiponectin is modified by the expression of specific skeletal muscle AdipoRs, the predominant isoform expressed in skeletal muscle.^420^ Muscle‐specific AdipoR1‐knockout mice exhibited decreased expression of molecules involved in mitochondrial biogenesis, such as PGC‐1α, as well as IR and decreased muscle endurance.^421^ Aging‐related decreased levels of AdipoR1 in skeletal muscle might contribute to sarcopenia with impaired muscle function and metabolic defects.^412^ However, until now, the evidence of a causal relationship between age‐associated sarcopenia and adiponectin remains controversial. On the one hand, some results have shown that the level of serum adiponectin is lower in sarcopenic older adults than in normal adults.^393^, ^422^ On the other hand, other studies showed a negative association between serum adiponectin levels and muscle strength, muscle density, and function among older adults.^423^, ^424^, ^425^, ^426^, ^427^

Adiponectin also regulates WAT, as well as exerting profound effects on converting WAT to BAT.^428^ Obese mice exhibited decreased muscular lipid content and reduced IR after adiponectin treatment.^429^ Knockout of adiponectin in sWAT of mice severely impaired beige adipocyte activation and thermogenic programs induced by chronic cold stimulation, while resupplementation with adiponectin reversed these changes.^428^ These results suggest that adiponectin might act as a compensator for metabolic diseases. However, AdipoR1 declines in aging, obesity, and T2DM,^430^ weakening beneficial adiponectin signaling to alleviate the disruptive effects of metabolic diseases.

Overall, adiponectin, an adipokine, regulates both muscle and adipose tissue to increase energy expenditure, which could be a bridge in maintaining homeostasis of both adipose tissue and skeletal muscle. Interestingly, adiponectin and AdipoR1 levels decrease in individuals suffering from both aging and obesity,^412^, ^430^, ^431^, ^432^ indicating that downregulation of adiponectin signaling could result in sarcopenia and sarcopenic obesity and should be considered a therapeutic target for muscle dysfunction and metabolic defects. Moreover, adiponectin can regulate various adipose tissue depots and is released from different adipose tissue sources, suggesting a complex and unclear role of this adipokine in cancer cachexia. Notably, previous studies have demonstrated that different adipose depots undergo remodeling during cachexia in a heterogeneous and time‐dependent manner.^433^, ^434^ This indicates that adiponectin may play a role in the pathogenesis of cachexia. Future research should aim to verify this hypothesis.

##### Resistin

Resistin, a hormone secreted by adipose tissue, appears to be a potential connecting link between adipose tissue and muscle. Resistin can disturb glucose homeostasis in rodents.^435^, ^436^ It acts as a potential direct regulator of glucose homeostasis by regulating muscle. Palanivel et al.^437^, ^438^ treated L6 rat skeletal muscle cells with recombinant resistin and found decreased fatty acid uptake and insulin‐stimulated GLUT4 levels, indicating that resistin might participate in communication between fat and muscle. This hypothesis was confirmed by an experiment performed by O'Leary, who found that the obese sWAT secretome derived from old but not young individuals impaired human myotubes, because the concentration of resistin was significantly greater from obese adipose‐conditioned medium than from normal weight older individuals, which exerted detrimental effects on myoblasts by activating the classical NF‐κB pathway. In turn, depletion of resistin from the obese sWAT secretome prevents its detrimental effects on muscle.^439^ Continuous expression of resistin in C2C12 cells by stable transfection inhibited myogenic differentiation, and reduced glucose uptake.^440^

In addition, human plasma resistin levels are higher in elderly subjects than in young individuals and are inversely correlated with muscle strength.^439^, ^441^ Bucci reported that plasma resistin concentrations are inversely associated with quadriceps muscle torque in people aged 69–81 years.^442^ Besides, levels of resistin are higher in obese elderly people than in normal‐weight elderly people, further suggesting that visceral adipose tissue‐derived resistin is involved in muscle weakness in individuals with sarcopenic obesity.^439^, ^443^

Resistin has been shown to stimulate human blood leukocytes to secrete TNF‐α and IL‐6. Interestingly, its expression is also stimulated by proinflammatory cytokines such as IL‐1, TNF‐α, and IL‐6, indicating a proinflammatory loop where resistin both contributes to and is induced by inflammation.^444^, ^445^ A recent meta‐analysis linked increased plasma resistin levels to a higher incidence of obesity‐related cancers, including breast, endometrial, and colorectal cancer; however, resistin levels should not be considered predictive factors.^446^ Additionally, high serum resistin levels in breast cancer patients have been positively correlated with tumor stage, size, and metastasis. Furthermore, elevated resistin levels have also been associated with an increased risk of nonobesity‐related malignancies, such as gastric, colon, and lung cancers.^447^, ^448^, ^449^ However, studies exploring the relationship between resistin and cancer cachexia remain limited at this time.

Collectively, the evidence suggests that resistin is associated with inflammation and serves as a link between adipose tissue and muscle, highlighting potential therapeutic targets for preventing sarcopenia, sarcopenic obesity, or cachexia. However, further studies are needed to confirm this hypothesis.

##### IL‐6

IL‐6 is a cytokine produced by multiple tissues in the body, including skeletal muscle, adipose tissue, and immune cells. IL‐6 binds to its receptor and activates the classical or trans‐signaling pathway, exerting its biological effect.^450^ IL‐6 is one of the most‐investigated myokines related to communication between skeletal muscle and WAT and exerts both pro‐ and anti‐inflammatory effects, either promoting muscle anabolism or catabolism depending on its source (secreted by muscle cells vs. adipocytes).^49^

IL‐6 could regulate the homeostasis of muscle. In brief, activated FAPs exhibit high levels of cytokine expression, including IL‐6. While IL‐6 is commonly known as a myokine secreted by myofibers in response to exercise, its expression in regenerating muscle is approximately 10 times higher in FAPs compared with myogenic progenitor cells.^144^, ^451^ IL‐6 plays a role in regulating both muscle hypertrophy and regeneration.^452^ IL‐6 knockout mice established a decline hypertrophic response to overloading, attributed to the impaired incorporation of myonuclei, which is a result of defective proliferation and migration of satellite cells. Treating murine satellite cells with IL‐6 has been shown to promote their proliferation. This effect is achieved through the regulation of cell‐cycle associated genes Cyclin D1 and c‐Myc,^453^ whereas IL‐6/STAT3 axis plays a crucial role in controlling the fate of satellite cells.^454^ Both intramuscular IL‐6 mRNA expression and protein release are enhanced during exercise when intramuscular glycogen is reduced,^455^, ^456^ while glucose ingestion during exercise has been shown to attenuate leg IL‐6 production,^457^ indicating IL‐6 is an energy sensor that regulates muscle glycogen content. Besides, IL‐6 increases glucose transport in L6 myotubes and skeletal muscle strips from vastus lateralis biopsies.^458^, ^459^ Human data also show that IL‐6 released during exercise leads to an increase in insulin‐stimulated whole‐body glucose disposal through an increase in translocation of GLUT4 from intracellular pools to the plasma membrane.^458^ Additionally, incubation with IL‐6 increases fatty acid oxidation in isolated rat muscle and L6 myotubes.^458^, ^460^ Because IL‐6 is crucial to maintaining proper metabolism,^461^ this might explain why regular physical exercise ameliorates metabolic diseases by regulating IL‐6. More specifically, the effects of IL‐6 on enhancing glucose uptake and fatty acid oxidation in skeletal myotubes are exerted by increasing AMPK.^458^, ^462^ Activation of AMPK in mice is accompanied by the browning of white fat by upregulating the downstream PGC‐1α,^463^ indicating that IL‐6 also participates in fat browning.^464^ This is demonstrated by the following results. WAT conversion into beige adipose tissue is indirectly affected by muscle‐secreted IL‐6.^465^ Whole‐body deletion of IL‐6 completely suppresses the elevation in UCP1 mRNA and protein stimulated by both cold exposure and exercise,^464^ while expression of UCP1 in mouse inguinal WAT increases upon injection of recombinant IL‐6.^464^ However, whether classical or trans‐signaling is involved in IL‐6‐induced adipocyte browning is still incompletely understood. This evidence suggests that IL‐6 secreted by muscle is involved in adipose tissue regulation.

In turn, numerous inflammatory cytokines and adipokines released by adipose tissue can enter the systemic circulation and act as paracrine hormones on skeletal muscle, including IL‐6. IL‐6 secreted by adipose tissue is the main source of circulating IL‐6, as Whitham et al.^466^ found that circulating IL‐6 was approximately threefold higher in control mice than that in adipocyte‐specific IL‐6‐deficient mice. In addition, obese individuals expressed higher visceral levels of IL‐6 protein compared with normal‐weight controls,^467^ which has a more significant correlation with diabetes than total body fat.^468^, ^469^ The proinflammatory effects of IL‐6 signaling contribute to age‐induced chronic low‐grade inflammation and may participate in the pathogenesis of sarcopenia.^470^ This indicates that IL‐6 secreted by adipose tissue may cause sarcopenia. Consistent with this speculation, clinical studies showed that levels of IL‐6 are dramatically upregulated in patients with sarcopenia after adjustment for potential confounders such as BMI and visceral fat tissue.^471^, ^472^, ^473^ IL‐6 stimulates muscle atrophy by blunting muscle anabolism and energy homeostasis and also mediates muscle catabolism directly through the following mechanisms. First, IL‐6 increases ubiquitin, E3 ligase, and proteasome activity levels by activating the NF‐κB pathway.^233^, ^474^, ^475^ Second, lasting IL‐6 secreted by FAPs in persistent inflammatory conditions including both aged and dystrophic muscle during regeneration stimulates muscle atrophy via activation of aberrant STAT3–IL‐6 signaling.^476^, ^477^ Third, continuously elevated IL‐6 directly induces dynamin‐related protein‐1 and fission protein 1 expression in myotubes, leading to dysfunction of mitochondrial function in muscle.^478^, ^479^, ^480^, ^481^, ^482^, ^483^, ^484^, ^485^ However, another study using an experimentally‐induced sepsis model found that knockout of IL‐6 exerts no significant influence on muscle metabolism compared with wild‐type mice,^486^ indicating that IL‐6 might not be the sole trigger for sarcopenia.

It can be challenging to determine the precise impact of IL‐6 on muscle regeneration because multiple cell types within the regenerative environment are known to secrete IL‐6. This makes it difficult to isolate and assess the specific effects of IL‐6 secretion from individual cell types in the process of muscle regeneration.^452^ To assess the direct influence of IL‐6 derived from FAPs on myogenesis, future studies will require loss‐of‐function experiments involving cocultures of FAPs and MuSCs or the targeted deletion of IL‐6 specifically in FAPs during muscle regeneration. These experiments will provide valuable insights into the specific role of FAP‐derived IL‐6 in the process of myogenesis. The potential role of IL‐6 in sarcopenia is complex, as it has dual roles in muscle physiology and pathology. We propose that IL‐6 signaling may shift toward the proinflammatory profile and participate in sarcopenia progression when skeletal muscle function gradually decreases during aging. Increased IL‐6 might thus reflect sarcopenia risk. In addition, IL‐6 secreted by adipose tissue might be involved in the pathogenesis of sarcopenia. This assumption needs further confirmation to identify other molecules that interact with IL‐6 to cause muscle dysfunction. Furthermore, a broader role for IL‐6 in promoting cachexia in cancer is suggested by a recent systematic review of gene polymorphisms associated with cancer cachexia. This review identified IL6 as one of four genes linked to key pathways, such as inflammation and metabolism, involved in the development of cachexia.^487^

##### IL‐8

IL‐8 belongs to the cysteine‐X‐cysteine family of chemokines and can be secreted by muscle fibers.^488^ Elevated levels of IL‐8 have been found in muscle fibers and plasma after exercise, which regulates muscle itself.^489^, ^490^, ^491^ A recent study reported that increased levels of plasma IL‐8 during the training period were negatively correlated with strength gains for the leg press.^492^ Elevated levels of IL‐8 have been related to lower extremity lean body mass (LBM) and a greater risk of sarcopenia in the UK elderly community.^493^ Another study showed that higher levels of IL‐6 and IL‐8 are a prominent pathological feature of sarcopenia in elderly people.^494^ Increased levels of IL‐8 secreted from T2DM myotubes create a muscle microenvironment that supports reduced capillarization in T2DM.^495^, ^496^ However, whether the causal relationship between IL‐8 and sarcopenia exists needs further investigation.

Adipose tissue is another organ that generates IL‐8, and vWAT releases higher amounts of IL‐8 than sWAT.^497^, ^498^ Different obesity models are associated with increased level of IL‐8 in adipose tissues. For example, estrogen‐deficient rats have increased fat mass, which is accompanied by increased production of IL‐8 in adipose tissue, and these results were reversed by estrogen replacement.^499^ Alvehus et al.^500^ found that the menopausal transition is characterized by increased body fat accumulation, and expression of IL‐8 is elevated (64%) in the sWAT of postmenopausal women compared with premenopausal women. IL‐8 expression in adipose tissue is profoundly decreased (90%) 2 years after gastric bypass surgery in obese women. Significant weight loss and reduced macrophage infiltration in sWAT as well as decreased IL‐8 mRNA levels were observed after short‐term lifestyle intervention.^500^ These results reveal that high levels of IL‐8 are released from adipose tissue and that the accumulation of this tissue partly leads to increased circulating IL‐8 in obese subjects.^497^ Lihn et al.^501^ reported that the secretion and gene expression of IL‐6 and IL‐8 from human adipose tissue fragments as well as from cultured human skeletal muscle cells were inhibited by treatment with AICAR, an AMPK activator known to increase insulin sensitivity. This demonstrated that the AMPK signaling pathway is involved in myokine and adipokine IL‐8 production. It has been found that IL‐8 inhibits insulin‐induced AKT phosphorylation in human adipocytes, resulting in IR.^502^ Thus, higher levels of IL‐8 in both plasma and vWAT are associated with IR, contributing to T2DM and obesity.^503^, ^504^ In addition, elevated IL‐8 levels in human adipocytes could be stimulated by TNF‐α treatment, indicating that higher IL‐8 might not be the primary trigger for IR and may exert this effect by stimulating TNF‐α.^505^

In conclusion, muscle release of IL‐8 might be negatively associated with insulin sensitivity and muscle function, while IR caused by adipose tissue secreted IL‐8 was mainly mediated through TNF‐α. This evidence suggested that IL‐8 might be involved in the muscle‐fat crosstalk in sarcopenia. Despite extensive attempts to decipher the relationship between IL‐8 and skeletal muscle, this issue remains unclear. Further studies are needed to evaluate the relevance of IL‐8 in the development of sarcopenia and to clarify whether it can be offered as a potential therapeutic target for this disease. Elevated serum IL‐8 levels have been associated with weight loss and significantly correlated with cachexia in pancreatic cancer patients.^506^, ^507^, ^508^ In gastric cancer patients, an IL‐8 genetic polymorphism has been linked to the onset and development of cachexia.^507^, ^508^ It is also important to note that IL‐8 is produced by both muscle and adipose tissue, particularly visceral fat, suggesting that the modulation of this cytokine can occur from various tissue.

##### IL‐15

IL‐15 is considered a myokine since it is released by muscle. Its serum levels are markedly elevated in response to contraction and do not last much longer.^509^ Tamura et al.^509^ found that the serum IL‐15 concentration was elevated 10 min after 30 min of exhaustive exercise. At 3 h after treadmill exercise, serum IL‐15 levels had fully returned to near baseline.^509^

IL‐15 treatment increases myotube development and induces myoblast differentiation, exerting hypertrophic effects.^510^, ^511^ Quinn et al.^511^ found that retroviral overexpression of IL‐15 induced a marked increase in myofibrillar protein accumulation in skeletal myogenic cell cultures, leading to a hypertrophic myotube morphology. Besides, IL‐15 may exert an antiatrophic effect, maintaining muscle mass in the presence of atrophic stimuli such as cancer cachexia, decelerating protein degradation and suppressing the ubiquitin proteolysis pathway in rats.^512^ IL‐15 decreases the skeletal muscle proteolytic rate in rat extensor digitorum longus muscle.^513^ Furthermore, IL‐15 might exert beneficial effects on muscle metabolism by improving oxidative characteristics and glucose homeostasis. In vitro, C2C12 myotubes treated with recombinant IL‐15 exhibited elevated basal and insulin‐stimulated glucose uptake,^514^ GLUT4 translocation,^515^ and GLUT4 mRNA levels.^514^ Incubation of C2C12 cells with recombinant IL‐15 (100 ng/mL) for 24 h showed increased GLUT4 translocation and glucose uptake, and this effect was abolished by STAT3 inhibition in combination with IL‐15.^515^ Additionally, increased circulation levels of IL‐15 may be beneficial for muscle by stimulating the expression of mitochondrial‐associated factors, such as PPARs and SIRT1, in mouse skeletal muscle.^516^

In addition to its effects on muscle, IL‐15 also reduces adipocyte lipid content, which is involved in muscle–fat interactions. For example, transgenic mice with skeletal muscle‐specific oversecretion of IL‐15 show lower total body fat when fed an HFD, along with lower intra‐abdominal fat and increased insulin sensitivity.^511^, ^517^ Barra et al.^518^ reported that a muscle‐specific IL‐15‐overexpressing mouse model displayed increased mitochondrial activity and mass in adipose tissue compared with IL‐15 knockout mice. In the same experiment, they also treated 3T3‐L1 adipocytes with IL‐15 (250 ng/mL) for 24 h and showed increased mitochondrial membrane potential and decreased lipid deposition.^518^ Thus, IL‐15 induces adipose tissue catabolism and decreases adiposity, regulating body composition.^98^ In addition, Quinn et al.^519^ found that IL‐15 decreased adiposity and lipid incorporation into adipose tissue, partly by elevating adiponectin secretion. Recent research has identified IL‐15 as a myokine that can prevent intramuscular fatty infiltration by potentially impacting the differentiation capacities of FAPs. The study demonstrated that IL‐15 promotes proliferation of FAPs and directly inhibits their adipogenic differentiation both in vitro and in vivo, ultimately facilitating regeneration of myofibers.^141^ Furthermore, the administration of recombinant IL‐15 directly into the muscle prevented fat accumulation in a murine model of glycerol‐induced fatty degeneration.^141^ The decrease in adipogenesis mediated by IL‐15 is associated with an upregulation of the desert hedgehog signaling pathway and its downstream effector, tissue inhibitor of metalloproteinase 3. This pathway is regulated by primary cilia present in FAPs, which have been shown to suppress the adipogenic differentiation of FAPs.^520^ The above studies suggested that IL‐15 could reduce the amount of adipose tissue through the muscle‐fat interaction. While the results suggest a positive role for IL‐15 in muscle regeneration, it is important to note that there is evidence correlating IL‐15 administration and expression with increased collagen deposition in vivo following muscle damage. This raises several unresolved issues that require further investigation to fully understand the complex effects of IL‐15 in muscle regeneration and fibrosis.^141^

The obvious involvement of IL‐15 in the axis between muscle and adipose tissue plays a key role in improving metabolism by inhibiting obesity and promoting insulin sensitivity. A cross‐sectional study showed that there was a negative relationship between IL‐15 levels and sarcopenia risk in older people.^521^ Significant reductions in circulating IL‐15 and soluble IL‐15 receptor alpha (IL‐15Rα) levels and the expression of intramuscular IL‐15 and IL‐15Rα were observed in advanced age.^522^ These alterations may weaken the beneficial effects of IL‐15 signaling and decrease the ratio of muscle to fat content,^523^, ^524^ resulting in sarcopenia.

The findings presented here provide a technical and experimental basis for the role of IL‐15 in muscle and fat physiology, suggesting that IL‐15 may act as a beneficial mediator of muscle regeneration and adipose tissue catabolism. Enhancing its signaling could be a promising therapeutic target for sarcopenia. However, the role of IL‐15 in cachexia remains unclear. An earlier study using a rat model of cancer cachexia indicated that IL‐15 reduces the rate of protein degradation without impacting protein synthesis.^512^ Additionally, research involving adult patients with recent cancer diagnoses and weight loss found no significant difference in serum IL‐15 levels compared with healthy subjects.^525^ Despite these conflicting results, the potential of this IL should not be dismissed, and further studies are necessary to clarify its role.

##### Irisin

Irisin is a myokine released into the bloodstream by cleavage of fibronectin type III domain‐containing protein 5 (FNDC5) triggered by muscle contraction.^526^, ^527^ However, adipose tissue, chiefly from WAT, is also another important source of irisin, while BAT expresses almost no FNDC5 or irisin.^528^ The expression of FNDC5 in adipose tissue is 100 times lower than that in muscle,^529^ indicating that muscle tissue is the primary source of irisin.

Interestingly, muscle itself is an important target of irisin. Mice serum and soleus muscle irisin level is increased following eight weeks of resistance training exercise and is negatively associated with age‐related decline in muscle function.^530^ Chang et al.^531^ found that a low level of circulating irisin is a sensitive marker of muscle dysfunction. In addition, in postmenopausal women, a reduced serum irisin level predicts the development of sarcopenia.^532^ Based on these clinical results, we speculate that decreased level of irisin might be a marker of sarcopenia.

Irisin can potentially reduce the risks of metabolic defects by improving skeletal muscle development and metabolism. During myogenic differentiation, human myocytes show elevated irisin synthesis, indicating the myogenic potential of irisin.^533^ Irisin treatment increases human primary skeletal muscle cell growth and hypertrophy by increasing IGF‐1/PGC‐1α4 and decreasing myostatin through activating the ERK1/2 pathway.^533^ Irisin has also been suggested to prevent glucocorticoid‐induced muscle atrophy by inhibiting FOXO‐mediated ubiquitin–proteasome overactivity.^534^ Treating human skeletal muscle cells with recombinant irisin (50 nM) for 1 h resulted in increased uptake of glucose and fatty acid in cells, similar to the effects of insulin.^535^ Knocking down the AMPK pathway attenuated the effects of irisin (0.3–1.0 µg/mL) on high glucose uptake and increased fatty acid β‐oxidation in the C2C12 myoblast cell line.^536^ Incubating myotubes with irisin increased the expression of genes related to mitochondrial biogenesis, such as PGC‐1α and Nrf1.^537^, ^538^ Immunohistochemical analysis on muscle sections with MyoD antibodies (a marker of activated satellite cells) revealed increased percentage of MyoD‐positive cells on day 2 and day 3 postnotexin‐induced injury in mice injected with irisin, when compared with DB‐injected controls. Treatment with irisin in vitro, resulted in a marked reduction in the percentage of quiescent satellite cells, concomitant with an increase in the percentages of proliferating (Pax7^+^/MyoD^+^) and committed myoblasts (Pax7^−^/MyoD^+^). These data suggest that irisin treatment leads to increased satellite cell activation and proliferation.^539^

Adipose tissues could be another target of irisin. Injection of irisin increased glucose utilization and reduced epididymal fat mass in diabetic mice by stimulating GLUT4 translocation in skeletal muscle.^536^ In obese mice, lentivirus‐mediated FNDC5 overexpression reduced hyperglycemia, hyperinsulinemia, hyperlipidemia, and adipocyte diameter in adipose tissues.^540^ Metformin acts as an antidiabetic drug, elevating intracellular FDNC5 mRNA/protein expression, stimulating irisin release in murine C2C12 myotubes,^78^, ^541^ and alleviating diabetes in mice.^541^ Furthermore, irisin has been shown to promote UCP1 expression in adipocytes, causing WAT to adopt a BAT‐like phenotype by stimulating energy expenditure, leading to lower body weight and promoting glucose homeostasis and IR.^527^ Interestingly, studies of circulating irisin in obese humans have produced inconsistent results. Some studies reported upregulation of serum irisin levels in overweight and obese people compared with normal controls,^542^, ^543^ whereas others found lower circulating irisin levels in obese or overweight people, compared with normal‐weight individuals.^544^ It is also possible that increased irisin levels could be a compensatory mechanism for the abnormal metabolism and insulin sensitivity characteristic of obese individuals, or who have developed a resistance to irisin suffered from obesity.^527^

A recent study found that intraperitoneal injection of recombinant irisin protein into ageing or aged mice could improve sarcopenia with grip strength, muscle weights, fiber size and molecular phenotypes and alleviated age‐associated fat tissues expansion, IR.^545^ This encouraging finding indicates that physical exercise in patients with sarcopenia may improve the function of aged muscle as well as energy metabolism through enhancing irisin expression, which may lead to the development of innovative approaches for the management of sarcopenia. It has been studied especially in relation to obesity but also with myopathies such as muscular dystrophy. Injection of irisin has been shown to induce muscle hypertrophy, enhance muscle strength, and reduce necrosis and the development of connective tissue in a murine model.^546^ This study may serve as a starting point for exploring therapeutic strategies involving irisin in the context of cancer cachexia. It is presumed that cell surface receptors can mediate FNDC5 activity and/or expression, but the features of this receptor are still unknown. Further studies provided other mechanistic insights into the irisin receptor. Clarification of the link among physical exercise, irisin and sarcopenia/sarcopenic obesity is needed, especially in humans, and evidence of a direct causal relationship is thus also missing.

##### Myostatin

Proliferation assays clearly demonstrate that adult myoblasts that lack myostatin proliferate much faster than the wild‐type myoblasts. In contrast, treated myostatin to myofiber explant cultures suppressed satellite cell activation by affecting the entry of satellite cells into S phase.^547^ Two RCTs tested the benefits of myostatin inhibitor, bimagrumab, in patients with sarcopenia. In a phase 2 proof‐of‐concept clinical study, Rooks et al.^548^ reported that treatment with bimagrumab over 16 weeks increased muscle mass and strength in older adults with sarcopenia and improved mobility in those with slow walking speed. However, these results were not confirmed in a recent phase 3 RCT,^549^ with no significant difference were found between participants treated with bimagrumab versus placebo among older adults with sarcopenia who had 6 months of adequate nutrition and light exercise, but both groups had physical function improvement. Bimagrumab treatment was safe, well tolerated, increased LBM, and decreased fat body mass.

Furthermore, myostatin might be involved in muscle‐to‐fat crosstalk. Research has shown that in obese patients, the mRNA levels of myostatin in muscle biopsies were significantly decreased after weight loss caused by biliary pancreatic diversion.^550^ Deletion of myostatin in mice also resulted in decreased fat mass, increased muscle mass, improved insulin sensitivity and resistance to diet‐induced obesity.^551^, ^552^, ^553^ Depending on the specific cell types and culture conditions, researchers have alternatively described the stimulation or inhibition of adipogenesis by myostatin. Its expression in mesenchymal cells promotes adipogenic differentiation through SMAD3, while exhibiting negative cross talk with β‐catenin.^552^, ^554^

Myostatin has been observed to inhibit lipid accumulation in cell lines of preadipocytes and fibroblasts.^555^, ^556^ Aged FAPs not only experience changes in the microenvironment that result in altered differentiation but also exhibit reduced responsiveness to muscle injury.^140^ The regulation of FAPs is attributed to myostatin, which plays a role in increased fibrosis as well.^557^, ^558^ In an in vitro study, it was demonstrated that myostatin induces enhanced proliferation and fibrotic differentiation of FAPs through upregulation of P‐SMAD2/SMAD3 signaling.^559^

According to previous studies, myostatin is a factor associated with the thermogenic capacity of BAT and the browning of WAT,^560^, ^561^ and a negative feedback loop of BAT activity has been reported. For example, Kim et al.^562^ reported that the differentiation of brown adipocytes and the expression of key thermogenic factors were dramatically inhibited by treatment with myostatin. Pharmacological inhibition of ActRIIB in mice blocks myostatin signaling, increases BAT activity and increases mitochondrial function, energy expenditure and cold tolerance.^563^ Braga et al.^564^ reported that treatment of myostatin null embryonic fibroblasts with exogenous myostatin significantly repressed thermogenic gene expression. Moreover, UCP1 and PRDM16 are significantly upregulated not only in epididymal and sWAT but also in gastrocnemius muscle in myostatin‐deficient mice compared with wild‐type mice.^564^ Kong et al.^565^ reported that loss of BAT, with thermoneutral temperature, increased myostatin levels, and reduced the exercise capacity of skeletal muscle. In contrast, activation of BAT decreased myostatin levels and improved exercise performance. Overall, inhibition of myostatin could be beneficial for obesity and associated metabolic disorders through browning of WAT.^463^

Interestingly, various lines of evidence suggest that myostatin could also be secreted by adipose tissue. For example, Hittel et al.^566^ reported that increased myostatin expression in myotubes and skeletal muscle are observed in obese women compared with lean women. However, inhibition of myostatin in adipose tissue has no effect on muscle mass and body composition in mice fed a HFD.^567^ In contrast, inhibition of myostatin signaling in skeletal muscle leads to increased lean mass, decreased fat mass in mice fed a HFD and resistance to diet‐induced obesity.^567^ McPherron and Lee^552^ reported that mice lacking the entire myostatin gene have a significant reduction in fat accumulation and skeletal muscle mass. These results suggest that myostatin expression in muscle but not adipose tissue could be the major target for obesity or diabetes.

Accumulating data show that myostatin increases with age, indicating that upregulation of myostatin protein secretion could result in sarcopenia by causing muscle wasting, along with increased fat mass.^568^ Myostatin mRNA and protein levels in muscle are significantly elevated by 2‐ and 1.4‐fold in old males compared with younger subjects, respectively.^569^ Similarly, skeletal muscle of elderly women expresses higher levels of myostatin mRNA (56%) than that of young women.^570^ However, other studies reported that concentrations of myostatin in either serum or muscle do not differ between young and sarcopenic elderly men.^571^, ^572^ These discrepancies indicate that myostatin may not be the primary trigger of sarcopenia. Interestingly, in the context of cachexia, circulating leptin levels—the “satiety hormone” secreted by adipocytes—are reduced in mice with myostatin deficiency, despite no significant difference in food intake compared with wild‐type mice.^551^, ^552^ Although there are relatively few studies examining myostatin expression in muscle cachexia, particularly as a biomarker and therapeutic target, we believe it presents a promising research avenue for cachexia treatment, especially when considered alongside decorin and leptin. Specifically, factors that change with aging, including exercise capacity, adiposity, and inflammation, can influence myostatin mRNA.^573^ Further studies are needed to explore the relationship between myostatin, cachexia, sarcopenia and aging, excluding other factors influenced by aging.

##### Meteorin‐like

There is evidence that meteorin‐β protein (Metrnβ), also known as meteorin‐like (Metrnl) or IL‐41, could be produced by both skeletal muscle and adipose tissue upon stimulation by exercise or cold exposure. In turn, this adipomyokine regulates both muscle and adipose tissue.^574^, ^575^, ^576^

In response to exercise, Metrnl released from muscle is dependent on the PGC‐1α signaling pathway. In contrast to PGC‐1α‐dependent FNDC5, Metrnl is upregulated after resistance exercise and is primarily dependent on PGC‐1α isoform 4 (PGC‐1α4).^577^ PGC‐1α4 does not regulate the targets of PGC‐1α1, such as mitochondrial and oxidative metabolism programs, but induces muscle hypertrophy and increased basal energy expenditure.^577^ The immune–muscle interaction during muscle repair has emerged as a critical process for successful regeneration. Both in vivo and in vitro experiment showed that Metrnl can suppress several proinflammatory genes, which, again were prevented by the Stat3 inhibitor, which serves to activate satellite cells through macrophage‐mediated IGF‐1.^578^ Aged mice (24–27 months old) had a profound decline in Metrnl expression when compared with the young controls.^579^ Administration of recombinant rMetrnl via in aged mice showed enhanced muscle regeneration. In vitro, they found that Metrnl treatment restores aged macrophage TNF‐α secretion, contributing to FAP apoptosis and suppression of profibrotic differentiation and fibrosis.^579^ To achieve this, Metrnl facilitates skeletal muscle repair through a Stat3/IGF‐1 mechanism.^578^, ^580^ Jung et al.^581^ found that Metrnl treatment markedly improved IR caused by palmitate in differentiated C2C12 cells and soleus skeletal muscle of HFD‐fed mice. Metrnl ameliorated lipid‐induced IR and inflammation via AMPK‐ and PPARδ‐dependent pathways in mouse skeletal muscle. In the same study, an HFD led to impaired glucose tolerance and decreased insulin sensitivity compared with control‐fed mice, and these changes were ameliorated by Metrnl administration.^581^ Lee et al.^582^ reported that Metrnl increased glucose uptake at doses ranging from 30 to 300 ng mL^−1^ and time points between 30 and 180 min in differentiated C2C12 myotubes. Metrnl exerts these beneficial effects via the calcium‐dependent AMPKα2 pathway in skeletal muscle cells. They also administered Metrnl to type 2 diabetic (db/db) mice and found that it improved glucose tolerance and reduced body weight.^582^ Overall, Metrnl secreted by muscle is involved in antagonizing IR, shedding light on the novel mechanisms by which physical exercise is beneficial for treating metabolic syndrome.

Metrnl in muscle could play a role in mediating muscle–fat crosstalk through upregulating the expression of genes associated with browning of WAT and stimulating energy expenditure.^574^ Interestingly, Metrnl induces brown adipocytes indirectly by regulating immune cells. Rao et al.^574^ reported that Metrnl stimulated an eosinophil‐dependent increase in IL‐4 and IL‐13 production and promoted alternative activation of adipose tissue macrophages and expression of thermogenic gene expression as well as anti‐inflammatory gene expression in WAT.

Moreover, it has been found that Metrnl is not only a myokine but also an adipokine. Li et al.^583^ used adipocyte‐specific Metrnβ transgenic and adipocyte‐specific Metrnβ null mice and revealed that adipocyte Metrnl antagonizes obesity‐induced IR by improving adipose function, including adipocyte differentiation, metabolic activation, and inflammation inhibition. The insulin sensitization of adipocyte Metrnl occurs through the PPARγ pathway.^583^

Considering that Metrnl is an adipomyokine that can regulate both muscle and adipose tissue and improve IR, it may be a promising new therapeutic target for the treatment of sarcopenia. Recently, research revealed a reduction of Metrnl expression with aging following injury.^580^ However, the alterations of Metrnl in sarcopenia patients remain unknown. Similarly, limited study explored the relationship between Metrnl and cachexia. One study indicates that lower serum levels of Metrnl are correlated with weight loss and the severity of cardiac dysfunction in elderly patients with chronic heart failure.^584^ Further research is warranted to clarify this hypothesis.

##### β‐Aminoisobutyric acid

β‐Aminoisobutyric acid (BAIBA) is released during muscle contraction in physical activity, regulating energy and acting as a new target for metabolic diseases. Roberts et al.^585^ analyzed metabolites secreted from myocytes (overexpressing PGC‐1α) and identified BAIBA, a metabolite derived from valine and thymine catabolism. They treated human‐induced pluripotent stem cells with BAIBA and discovered a brown adipocyte–like gene expression signature as well as elevated mitochondrial activity.^585^ BAIBA supplementation (100 mg/kg/day) for 4 months increased PGC‐1α and UCP1 expression together with glucose tolerance improvement and decreased weight loss in diet‐induced obese mice.^586^ BAIBA also reversed the suppressed insulin signaling, glucose uptake and fatty acid oxidation caused by lipopolysaccharide (LPS) treatment in differentiated 3T3‐L1 cells.^587^ This myokine stimulates energy expenditure by activating the β‐oxidation pathway of hepatic fatty acids, triggering the browning of WAT.^585^

In addition to its effects in adipose tissue, BAIBA also regulates muscle. Jung et al.^588^ found that BAIBA treatment significantly reversed impaired insulin sensitivity and increased fatty acid oxidation induced by obesity in isolated soleus skeletal muscle and C2C12 mouse skeletal muscle cells. Furthermore, BAIBA improved glucose tolerance and insulin tolerance in diet‐induced obese mice.^588^

Overall, BAIBA exerts beneficial effects on both muscle and adipose tissue through mechanisms such as regulating insulin receptor activity, indicating its potential as a novel agent for disorders associated with metabolic defects. However, there is still insufficient evidence regarding its alterations in sarcopenia, where it may merely act as a bystander. Clinical experiments are needed to confirm this hypothesis. The ability of BAIBA to regulate muscle and adipose function highlights its potential use to mitigate the detrimental effects of cancer cachexia on musculoskeletal health.

##### 12,13‐di HOME

Research has shown that BAT functions as an endocrine organ, not only producing batokines but also lipokines. Lipokines, which are a class of lipids, act as signaling molecules and have significant roles in influencing systemic metabolism.^589^, ^590^, ^591^, ^592^ Stanford University researchers discovered that a single session of exercise elevates plasma 12,13‐diHOME levels in both humans and mice.^591^ Following exercise, 12,13‐diHOME is released from BAT and has been shown to enhance fatty acid uptake and oxidation in skeletal muscle.^591^ Furthermore, cold exposure has been shown to activate BAT, making it reasonable to explore the role of 12,13‐diHOME following cold stimulation. As anticipated, both humans and mice exhibit increased circulating levels of 12,13‐diHOME after cold exposure.^591^ Injection of 12,13‐diHOME promotes BAT thermogenesis by specifically enhancing fatty acid uptake, thereby supplying fuel for BAT. Additionally, exercise‐induced release of 12,13‐diHOME from BAT serves as an endocrine signal, facilitating the transport of fatty acids into active skeletal muscle. This mechanism may contribute to the metabolic effects of exercise on regulating metabolism.^592^ Indeed, exercise‐induced irisin has been shown to promote the browning of WAT, suggesting the possibility of reciprocal signaling from muscle to BAT.^527^ The coordinated interplay between muscle and BAT in thermogenesis facilitates the beneficial outcomes of exercise. Additionally, administering 12,13‐diHOME to diet‐induced obese mice protects against both cold challenges and obesity induced by a HFD.^592^, ^593^ These findings suggest that 12,13‐diHOME, induced by BAT, plays a role in enhancing metabolic adaptations to exercise, potentially benefiting sarcopenia. Further research is needed to fully understand the functional implications of 12,13‐diHOME and its relevance to sarcopenia. Its ability to regulate muscle and adipose function indicates a potential role for this molecule in cancer cachexia.

##### TNF‐α

TNF‐α is secreted by multiple cells, including myocytes, adipocytes, macrophages, and other immune cells.^52^, ^594^ TNF‐α is suggested as a potential biomarker of sarcopenia. Elevated levels of TNF‐α are characteristic of community‐dwelling older adults with frailty and sarcopenia, independent of age.^471^ Li et al.^393^ compared sarcopenia and nonsarcopenia elderly individuals and reported that higher serum levels of TNF‐α (>11.15 pg/mL) were correlated with a 7.6‐fold increase in the risk of sarcopenia based on logistic regression analysis. Bian et al.^595^ reported that elderly individuals with elevated serum levels of TNF‐α were more likely to develop sarcopenia.

Muscle‐derived myeloid might be the increased source of TNF‐α in sarcopenia. In skeletal muscle of aged mice, there was an increase in the number of TNFα‐expressing macrophages with skeletal muscle.^203^ Older TNFα‐null mice demonstrate better muscle quality when compared with control group, with enhanced satellite cell activation and myogenic commitment in response to injury.^596^ TNF‐α, and conditioned medium from Th1 cells or activated macrophages has been shown to reduce myocyte insulin sensitivity.^228^, ^597^ Additionally, TNF‐α could be secreted by myocyte itself. Differentiated myocytes have been shown to express a multitude of proinflammatory molecules, particularly under the influence of inflammatory cytokines and FFAs.^598^ Differentiated cultured myocytes isolated from obese individuals with IR or T2DM have been found to secrete higher levels of cytokines such as TNF‐α and chemokines such as MCP‐1 compared with myocytes from lean controls. This indicates that the inflammatory response in myocytes is dysregulated in obesity‐related IR and T2DM,^224^, ^599^ aggravate vicious cycle. Indeed, there is evidence supporting the notion that overactivation of the NF‐κB pathway, which is an important target of TNFα, in the muscle of older mice can disrupt satellite cell function and impede regenerative success.^324^ The age‐related decline in satellite cell function is likely due to the overactivation of multiple inflammatory pathways working together synergistically.

On the other hand, TNF‐α might be beneficial for muscle regeneration. During the initial phase of muscle regeneration, it is believed that several proinflammatory cytokines, such as TNFα, IL1β, and IL6 are involved in initiating the inflammatory response, recruiting immune cells, and facilitating the activation of satellite cells, all of which contribute to the process of muscle repair and regeneration.^600^ Defects in muscle regeneration have been observed in mice lacking TNF‐α or its receptors, indicating the essential role of TNF‐α signaling in the process of muscle regeneration,^601^, ^602^ MuSC differentiation,^603^ and regulating FAP apoptosis.^145^ Recently, there has been a suggestion that in response to acute muscle damage, TNF‐α released by macrophages plays a crucial role in regulating the apoptosis of FAPs.^145^, ^604^ When TNF‐α‐producing macrophages are absent, FAPs tend to accumulate and undergo abnormal differentiation into a fibrogenic lineage.^145^ Studies have demonstrated that the TNF‐α‐mediated apoptosis of FAPs may be disrupted in a glycerol model of muscle injury. This disruption can result in the development of IMAT.^605^ In addition, macrophages can also produce TGF‐β1, which inhibits the TNF‐α‐mediated apoptosis of FAPs and instead promotes their fibrogenic differentiation. This leads to the deposition of ECM components in the muscle tissue. The above evidence suggested that TNF‐α could influence the regeneration or disruption of muscle, the opposing role might depend on different source, amount and environment.

Apart from muscle cells, TNF‐α could also be secreted by adipose tissues. It has been observed that TNF‐α levels in adipose tissue tend to increase with aging.^606^ Cultured primary preadipocytes have been shown to release TNF‐α.^607^ TNF‐α has been found to interfere with preadipocyte differentiation and can induce lipolysis, resulting in decreased fat cell size and reduced insulin responsiveness.^608^ After high fat feeding in cases of obesity, the expression of TNF‐α in adipose tissue increases. However, only a small amount of TNF‐α from adipose tissue actually enters the general circulation.^609^, ^610^ The localized elevation of TNF‐α in adipose tissue serve to prevent further accumulation of fat. Given the emerging role of adipose tissue in the progression of sarcopenia, increased TNF‐α signaling may associated with coalescence of sarcopenia and obesity. However, in the case of TNF‐α, plasma concentrations may be strongly influenced by circulating immune cells—and thus it is still unknown whether the source of “inflammaging” may be influenced by adipose tissue or skeletal muscle and that they may be the passive recipients of changes in other tissues or compartments. This open question needs further study by using specific knock out mice in muscle or adipose.

Furthermore, TNF‐α plays an important role in all key mechanisms via skeletal muscle loss, adipose tissue loss, alterations in carbohydrate, protein and lipid metabolism, IR, systemic inflammation in cancer cachexia. For instance, TNF‐α significantly contributes to increased gluconeogenesis, proteolysis, and the loss of adipose tissue in cachectic patients, and it is associated with the upregulation of UCPs 2 and 3 in cachectic skeletal muscle.^611^, ^612^, ^613^ Additionally, TNF‐α promotes the accumulation of neutrophils and macrophages in skeletal muscle.^614^ The increase in neutrophils and their infiltration into tumors is linked to poorer outcomes and more severe manifestations of cachexia.^615^ Therefore, future research should focus on modulating the associated pathways and altering the response to TNF‐α to develop therapeutic strategies for cancer cachexia. In the long term, such research may lead to effective management strategies that improve morbidity and mortality.

##### FGF21

FGF21, a member of the FGF superfamily, is expressed in both muscle and adipose tissues. It interacts with specific FGF receptors and a cofactor known as klotho. Various tissues, such as the liver, heart, pancreas, and WAT, release FGF21.^616^, ^617^ The relationship between FGF21 and muscle is intricate. The majority of evidence suggests that elevated FGF21 levels are associated with deteriorating health parameters^618^, ^619^ and are involved in the pathophysiology of sarcopenia by affecting muscle strength more than muscle mass. For instance, Conte et al.^618^ reported a positive and significant correlation between FGF21 and age. Additionally, FGF21 levels were associated with various deteriorated parameters, including handgrip strength, particularly in individuals around 70 years old. In a study conducted by Bag Soytas et al.,^619^ individuals over the age of 65 years exhibited lower handgrip strength and higher FGF21 levels, coupled with reduced capacity to complete a 6‐minute walk. Roh et al.^620^ examined 386 community‐dwelling older adults aged 70–84 years and discovered that circulating FGF21 levels were inversely associated with muscle strength but did not show an independent correlation with muscle mass. Soto et al.^621^ reported that acute exposure to low concentrations of FGF21 enhances glucose uptake in isolated muscle fibers through GLUT4 transporters. The contradiction could stem from the notion that elevated FGF21 serves as an adaptive regulator, countering muscle stress induced by mitochondrial dysfunction. This hypothesis gains support from the observation that FGF21 expression is detectable solely in muscles experiencing conditions like starvation, endoplasmic reticulum stress, mitochondrial dysfunction, or aging, rather than in healthy individuals.^617^ Indeed, further studies are imperative to establish the protective role of FGF21 in diseases linked to mitochondrial dysfunction.

FGF21 has been observed to negatively regulate the adipogenic differentiation of goat intermuscular preadipocytes in vitro by suppressing the expression of PPARγ and modulating the expression of various KLFs.^622^ Additionally, heightened FGF21 expression in skeletal muscle plays a role in shielding against diet‐induced obesity and IR,^623^, ^624^ indicating that muscle‐derived FGF21 could be a regulator of integrated energy and lipid metabolism. FGF21 achieves these beneficial effects by inducing weight loss, reducing body fat, and promoting the browning of WAT.^625^ For instance, HFD‐fed obese mice, subcutaneously injected with recombinant FGF21 (0.5 mg/kg) twice a day at approximately 12‐h intervals for 3 weeks, showed decreased levels of IL‐6 and TNF‐α in adipose tissue. This treatment also reversed HFD‐induced metabolic disorders, including obesity, glucose tolerance impairment, and IR.^626^ Furthermore, FGF21 treatment ameliorated hyperglycemia, dyslipidemia, and obesity in diabetic monkey models.^627^, ^628^, ^629^ FGF21 also exerts endocrine effects that promote increased browning of WAT, characterized by the upregulation of the UCP1 gene and PGC‐1α protein.^625^ However, other studies have reported that serum FGF21 levels in overweight/obese subjects or individuals with T2DM were significantly higher than those in normal individuals. This suggests that FGF21 could serve as an independent predictor of T2DM and obesity.^630^, ^631^, ^632^ Indeed, this contradiction further supports the notion that FGF21 acts as a compensatory factor for pathological diseases, including metabolic diseases and conditions related to mitochondrial dysfunction.

Moreover, FGF21 is produced and released by both BAT and WAT, functioning as an adipokine that enhances insulin sensitivity. Lin et al.^633^ documented the therapeutic advantages of FGF21 in addressing obesity‐induced hyperglycemia, hypertriglyceridemia, and peripheral IR by activating its downstream mediator, adiponectin.

In summary, these findings indicate that FGF21 is released as a protective mechanism against muscle dysfunction and metabolic abnormalities. Elevated serum levels may serve as a biomarker for sarcopenia and could potentially be utilized as a therapeutic intervention. However, FGF21 may play a passive role, being secreted in response to muscle damage or sarcopenia in an effort to restore balance. Additional experiments are necessary to elucidate the precise role of FGF21 and assess its safety profile. Moreover, FGF21 is often overlooked as a myokine in the study of cachexia, despite its demonstrated involvement in myocyte energy metabolism. Future research is warranted to explore its potential in therapeutic strategies, as maintaining normal energy metabolism in muscle tissue is crucial for overall health.

##### Crosstalk between myokines and adipokines

Myokines and adipokines engage in cross‐talk. For instance, when myotubes are exposed to TNF‐α, the expression of FNDC5 protein decreases.^678^ TNF‐α also influences leptin expression. Brief exposure to TNF‐α leads to an increase in leptin accumulation,^679^ while long‐term treatment decreases leptin production.^680^, ^681^ In obese TNF‐α‐deficient mice, circulating leptin levels are lower, while adipose tissue levels are higher compared with normal mice.^679^ Injection of leptin enhances irisin‐induced myogenesis in a manner dependent on PGC‐1α and inducible NO synthase, while concurrently suppressing the reduction of subcutaneous fat browning through FNDC5 transcription in subcutaneous adipocytes.^682^ Leptin increases the level of Prdm16,^682^ a transcription factor controlling the bidirectional cell fate switch between skeletal myoblasts and brown adipocytes.^683^, ^684^, ^685^ Based on the evidence, both Prdm16 and a proliferation step are necessary for the browning of white beige adipocytes. We hypothesize that leptin potentially participates in the proliferation of beige precursor cells and myogenesis. Utilizing mouse embryonic fibroblasts, primary cultures derived from myostatin knockout mice exhibited increased adiponectin protein levels compared with the wide‐type group, leading to enhanced lipid metabolism and energy expenditure.^564^ Furthermore, myostatin deficiency results in significantly elevated skeletal muscle mass and decreased fat mass, potentially due to heightened leptin sensitivity and increased energy expenditure.^686^

In summary, the interplay between myokines and adipokines suggests that improved muscle function can positively influence fat metabolism. However, in conditions such as sarcopenia or cachexia, this communication is disrupted due to muscle dysfunction, which may exacerbate these conditions by affecting the regulation of myokines and adipokines. Therefore, targeting this interplay could be a promising approach for treating sarcopenia and cachexia.

#### 4.3.2 miRNAs act as candidate mediators for adipose–muscle axis

Evidence suggests that miRNA levels in muscle can undergo changes during the ageing process, and these alterations may have negative effects on both the quality and quantity of muscle tissue.^687^ Studies have shown that miRNAs derived from adipose tissue can be transported to different host cells, such as myocytes, hepatocytes, and macrophages, via exosomes.^688^ Similarly, miRNAs that originate from skeletal muscle can be absorbed by adipose tissue.^689^ Functionally, this communication between organs has been associated with IR, adipogenesis, and lipid metabolism, indicating that miRNAs may play a role in the development of sarcopenia or cachexia.^689^, ^690^

##### MiR secreted by adipose tissue (miR‐130b and miR‐33a)

Evidence suggests that adipose tissue can mediate muscle through miRNA. Wang et al.^634^ demonstrated that miR‐130b is released by adipocytes during the process of adipogenesis. The expression of miR‐130b in WAT increases with obesity. Additionally, circulating levels of miR‐130b are higher in both human and murine obesity, and these levels are positively correlated with BMI and predictive of the metabolic syndrome.^634^ Not only adipose tissues, but also muscle tissues of ob/ob mice exhibited an elevated level of miR‐130b precursor. Moreover, miR‐130b can target muscle cells and decrease the expression of PGC‐1α, which is a key regulator of lipid oxidation in muscle tissue.^634^ More experiments are needed to clarify the relationship between miR‐130b in sarcopenia, sarcopenic obesity or cachexia.

Another miRNA that might present candidate mediators of the adipose–muscle crosstalk is miR‐33a, which is expressed in both adipose tissue and muscle tissue and influence themselves. MiR‐33a controls cholesterol and lipid metabolism.^635^ Mice deficient in miR‐33a become obese with increased adipose tissue expansion, increased lipid uptake, and impaired lipolysis and IR.^636^ Primary duck myoblasts treated with a miR‐33a mimetic showed impaired proliferation, whereas a miR‐33a inhibitor elevated proliferation. miR‐33a suppresses myoblast proliferation by inhibiting the PI3K/Akt/mTOR signaling pathway.^637^ This evidence indicates that miR‐33a is involved in adipocyte and lipid regulation; however, overexpression of miR‐33a might impair myogenesis. Decreased levels of systemic miR‐33a with aging combat muscle impairment. More experiments are needed to investigate the role of miR‐33a in sarcopenia or cachexia. Overall, miR‐33a might be involved in the crosstalk between muscle and adipose, which needs further study.

##### MyomiRNA

Recent studies have indeed confirmed the specific expression of certain miRNAs in muscle cells, such as miR‐206 and miR‐133, which play critical roles in muscle growth and development. These miRNAs, known as myogenic miRs (myomiRs), including miR‐133 and miR‐206, target alternative BAF60 subunits, promoting promyogenic differentiation while suppressing the FAP phenotype and inhibiting the deposition of ectopic adipose tissue.^691^The evidence suggests that myomiRs could potentially be therapeutic candidates for addressing sarcopenia or cachexia by modulating the communication between adipose and muscle tissues.

##### MiR‐133b

MiR‐133b, secreted by muscle, is a muscle‐specific miR, which plays important roles in myogenic proliferation and differentiation, but its exact function remains controversial. It has been reported that miR‐133 stimulates myoblast proliferation while hindering myoblast differentiation,^638^ but some studies suggest that miR‐133 promotes myogenic differentiation.^639^, ^640^, ^641^ Different experimental models, such as cardiac or skeletal myocytes, and different situations might account for the discrepancy.

In addition, there is evidence that miR‐133b reflects muscle repair ability. miR‐133b is elevated in skeletal muscle during regeneration following injury, and injection of miR‐133b alongside miR‐1 and miR‐206 accelerates muscle regeneration in a rat skeletal muscle injury model.^642^ Therefore, it is speculated that with the ability of muscle to repair itself becomes worse in elderly individuals, the expression of miR‐133b might decrease accordingly. Iannone et al.^643^ reported that miR‐133b expression is reduced in sarcopenic patients. According to Drummond et al.,^644^ global miR expression microarray data showed that miR‐133a/b expression in the skeletal muscle of elderly men was decreased compared with young men.

Apart from above mentioned functions, this muscle‐derived miR could influence adipose tissues as well. For instance, literature has shown that obesity is associated with downregulation of miR‐133b in WAT.^645^ Yin et al.^646^ found that miR‐133 targets Prdm16 3′UTR and controls brown adipose determination. In addition, miR‐133 antagonism induces active brown adipocytes within regenerating muscle and impedes the development of obesity.^646^ Since WAT and brow adipose tissue are both related to the pathogenesis of sarcopenia and cachexia, the therapeutic effect of the myogenic miR‐133 needs further investigation.

However, many factors affect the aging process and need to be considered, such as ROS, nutrition, and lifestyle.

##### MiR‐206

Muscle‐specific miR‐206 plays an important role in the differentiation and development of skeletal muscle. Gagan and colleagues^647^ found that induced miR‐1 and miR‐206 could directly downregulate Notch3 and allow myoblast differentiation. Inhibition of endogenous miR‐1 and miR‐206 blocks downregulation of Notch 3, one of the major targets in differentiating cells,^647^ suggesting that miR‐206 promotes skeletal muscle differentiation. Furthermore, miR‐206 regulates myogenesis by inhibiting connexin 43 expression, a factor needed for skeletal myoblast fusion,^648^ as well as myostatin, a negative factor for muscle growth.^649^ MiR‐206 also participates in skeletal muscle growth and regeneration by repressing the expression of *Pax7*, a marker of skeletal muscle satellite cells.^650^, ^651^

MiR‐206 is involved in pathological processes in skeletal diseases as well. MiR‐206 is increased in several diseases to promote muscle regeneration and to increase resistance to muscle atrophy.^642^, ^652^ However, there are insufficient studies investigating the relationship between miR‐206 and aging. Kim et al.^653^ used next‐generation sequencing technology to analyze mRNA and miR expression in young (6 months) and aged (24 months) mouse gastrocnemius muscle and found that 20 miRs, including miR‐206, were upregulated in aged muscle, while nineteen were downregulated. Hamrick et al.^654^ compared the miR expression profiles between mice at 12 and 24 months of age and found that miR‐206 was upregulated approximately 2‐fold in aged mice compared with young mice. Moreover, compared with young men, miR‐206 was upregulated in skeletal muscle from old men.^655^ These data suggest that miR‐206 might be related to resistance to muscle atrophy during the aging progression. In addition, miR‐206 might be influenced by obesity which might adversely affect myogenesis, as its expression is 0.5‐fold downregulated in plasma from overweight/obese children compared with normal‐weight children.^656^

Evidence suggests that myogenic miR‐206 was also specifically expressed in both brown pre‐ and mature adipocytes.^657^ Xu and colleagues^658^ found that overexpression of miR‐206 in adipocytes decreased cell proliferation and TG accumulation in primary cultures of adipocytes from pigs, which suggested that miR‐206 acts as a suppressor of adipogenesis. These data demonstrated that miR‐206 could act as a messenger in muscle‐regulated adipogenesis, which might be a potential target in the treatment of sarcopenia and cachexia. However, the relationship between miR‐206 and sarcopenia or cachexia may be complex, as miR function is influenced by various contexts. Further bioinformatics analysis is necessary to validate its potential use in the treatment and diagnosis of sarcopenia and cachexia in future investigations.

##### miR‐424 ‐5p (miR‐322)

MiR‐424 ‐5p, identified in skeletal muscle, plays an important role in muscle wasting. miR‐424 ‐5p has been reported to target IGF‐1 in vitro,^659^ which is one of the most important mediators of muscle growth and repair.^660^ In vitro, miR‐424‐5p reduced expression of rRNA and inhibited protein synthesis in muscle cells, and overexpression of miR‐322 (rodent miR‐424 orthologue) caused fiber atrophy and reduced upstream binding transcription factor expression and rRNA levels in mice.^661^ miR‐424 ‐5p could target SMAD7, a strong inhibitor of the TGF‐β pathway.^661^ TGF‐β pathway involves genes related to muscle atrophy, which verifies the role of miR‐424 ‐5p in muscle wasting.

Apart from the effect on muscle, miR‐424 ‐5p also has a potential link to adipose tissues. IL‐4 induces the proliferation of bipotential adipocyte precursors in white fat tissue and primes these cells for differentiation into beige adipocytes, which are specialized for thermogenesis, and miR‐424‐5p (miR‐322) is upregulated upon IL‐4 stimulation.^662^ Gasparotto et al.^663^ collected sWAT from obese and nonobese women and found that miR‐424‐5p was significantly correlated with waist circumference in nonobese women. Central and total sWAT thicknesses were correlated with miR‐424‐5p levels in the nonobese group as well. In the obese group, miR‐424‐5p expression was correlated with BMI. MiR‐424 were correlated with higher fat depot measurements in nonobese women.^663^ Additionally, studies have reported that miR‐424 ‐5p is upregulated in both obesity and sarcopenia,^661^, ^690^ suggesting it might play a role in the pathogenesis of sarcopenic obesity.

These evidence suggested that miR‐424‐5p might be a involved in the pathogenies of sarcopenia and cachexia, which need further investigation.

#### 4.3.3 lncRNAs: potential roles in sarcopenia and cachexia

Roughly 1% of the RNA transcribed from the human genome consists of protein‐coding gene sequences, whereas noncoding RNA makes up a significantly larger proportion.^692^ LncRNAs are defined as RNA molecules that are longer than 200 nucleotides. While they do not directly encode proteins, they play a crucial role in regulating gene expression at both the transcriptional and posttranscriptional levels, thereby influencing biological functions. Research has shown that lncRNAs are involved in various diseases, including obesity and muscle wasting.^693^, ^694^

##### H19

H19 is a lncRNA that is highly expressed in adult muscle tissues and elevated during myoblast differentiation and regeneration.^664^, ^665^ Overexpression of H19 facilitates muscle regeneration, while deficiency impairs muscle differentiation.^666^, ^667^, ^668^ Mice treated with an H19 gain‐of‐function (GOF) mutant (AGR–H19–Rgof) were resistant to HFD‐ and *leptin* deficiency‐induced obesity. In addition, AGR–H19–Rgof also significantly improved the muscle growth and strength of wide‐type animals.^668^ H19 improves glucose metabolism via miR‐106a‐5p/E2F3^669^ and the PI3K–Akt signaling pathway,^670^ while expression of H19 is decreased in the skeletal muscle of both T2DM patients and HFD‐induced obese mice and impaired glucose metabolism.^670^ Moreover, H19 interacts with hnRNPA1 to increase translation of fatty acid oxidation‐related genes, including CPT1b and PGC‐1α, modulating lipid metabolism.^671^

Taken together, these results indicate that H19 influences muscle regeneration, glucose, and lipids metabolisms, which might be potentially involved in the crosstalk between adipose tissue and muscle and is a potential biomarker for sarcopenia and cachexia. Future studies are needed to identify the exact relationship between sarcopenia and H19.

##### MALAT1

The expression of the MALAT1 gene experiences a rapid and persistent rise during the initiation of both mouse and human skeletal myogenesis, suggesting its involvement in the proliferation and differentiation of skeletal cells.^672^ Silencing MALAT1 not only impairs myoblast proliferation^672^ but also prevents normal myotube maturation and differentiation.^673^ Han et al.^674^ reported that inhibiting MALAT1 leads to the downregulation of Srf expression, a critical factor for myocyte differentiation, at both the RNA and protein levels in C2C12 cells, a process dependent on miR‐133. Additionally, MALAT1 exhibits a strong response to myostatin in skeletal muscle and may be involved in the inhibition of myogenesis. Treating proliferating human primary myoblasts with myostatin results in a 22% reduction in MALAT1 transcript levels.^672^ Recombinant myostatin treatment reduces MALAT1 expression.^672^ Downregulation of MALAT1 occurs with age.^673^ In contrast, Chen et al.^675^ discovered that MALAT1 is downregulated during early myogenesis and influences differentiation by regulating MyoD. These findings emphasize the role of MALAT1 in myogenesis.

Recent studies have suggested that knocking down MALAT1 significantly inhibits the process of adipogenesis in vitro.^676^ Furthermore, studies have demonstrated that adipocytes release MALAT1 within EVs, thereby affecting weight regulation in mice.^677^ Further research is necessary to elucidate this hypothesis and explore its implications in the context of obesity.

Given MALAT1's impact on both myogenesis and adipogenesis, it could potentially regulate the equilibrium between muscle and adipose tissue, offering a promising target for therapeutic interventions in sarcopenia and cachexia.

## 5 DIAGNOSTIC CHALLENGES AND BIOMARKERS

As conditions commonly associated with aging, sarcopenia and cachexia are marked by a significant decline in muscle mass, reduced quality, and diminished function, which heightens the risk of falls, disabilities, and even mortality among older adults. These conditions also place a considerable strain on the healthcare system and society as a whole. Consequently, research focused on the early screening, assessment, and intervention of sarcopenia and cachexia is of utmost importance. Currently, methods for assessment, effective screening tools, and intervention strategies for these conditions have become key areas of study, which can aid nursing staff in the early identification of individuals at risk, provide valuable opportunities for prompt diagnosis and treatment, and facilitate the thorough implementation of secondary prevention measures. Understanding the underlying mechanisms is crucial for developing diagnostic and therapeutic approaches. In light of the previous research discussed, we will also explore the potential roles of adipokines and myokines as diagnostic biomarkers and therapeutic targets in sarcopenia and cachexia.

### 5.1 Diagnostic criteria for sarcopenia and cachexia

Sarcopenia and cachexia can indeed occur concurrently^695^; particularly in older cancer patients, leading to poor outcomes.^696^, ^697^ While both conditions are characterized by muscle wasting, their underlying mechanisms differ.^698^ This makes clinical differentiation challenging, as there is no clear boundary or established screening tools for distinguishing between the two.^699^, ^700^, ^701^, ^702^

Sarcopenia is a syndrome characterized by the progressive and generalized loss of skeletal muscle mass and strength, which increases the risk of adverse outcomes such as physical disability, poor QOL, and mortality.^703^, ^704^ The European Working Group on Sarcopenia in Older People (EWGSOP) recommends that diagnosis requires both low muscle mass and low muscle function (strength or performance). Specifically, this involves documenting criterion 1 (low muscle mass) alongside either criterion 2 (low muscle strength) or criterion 3 (low physical performance).

Proposed items for new diagnostic criteria for cachexia have been categorized into four groups: subjective symptoms, objective measurements, biomarkers, and other parameters. Subjective symptoms include anorexia, fatigue, exhaustion, and weakness. Objective measurements encompass low BMI, weight loss, a weight loss grading system, BMI‐adjusted weight loss, loss of skeletal muscle mass, low fat‐free mass, grip strength, reduced muscle strength, physical performance, and overall weakness. For biomarkers, there is consensus on CRP, but no agreement on other specific markers.^705^

Both sarcopenia and cachexia share the common feature of muscle mass loss. While the EWGSOP and the Asia Working Group for Sarcopenia require low muscle mass and decreased strength for diagnosing sarcopenia, Evans et al.^6^ suggest that decreased muscle strength and/or low fat‐free mass are sufficient for diagnosing cachexia. Fearon et al.^7^ also propose including sarcopenia as a diagnostic criterion for cancer cachexia. Importantly, involuntary weight loss and reduced food intake (anorexia) in the context of chronic systemic inflammation and metabolic changes are key features of cachexia.^7^, ^706^ In contrast, sarcopenia is primarily age‐related, and weight loss is not a diagnostic criterion.^3^

### 5.2 Current diagnostic tools and biomarkers

#### 5.2.1 Validated tests and tools for sarcopenia and cachexia

##### Muscle mass

A wide range of techniques can be employed to assess muscle mass.^3^ Factors such as cost, availability, and ease of use often determine whether these techniques are more suitable for clinical practice or for research purposes. Accurately quantifying skeletal muscle mass remains a challenge, prompting the development of various measurement methods over the past two centuries (for a historical overview, see Ref. 707).

Three primary imaging techniques are commonly used to estimate muscle mass or LBM: computed tomography (CT), magnetic resonance imaging (MRI), and dual‐energy X‐ray absorptiometry (DXA). CT and MRI are regarded as highly precise imaging modalities capable of differentiating fat from other soft tissues, establishing them as gold standards for estimating muscle mass in research contexts.^3^, ^11^, ^708^ However, their high costs, limited accessibility, and concerns regarding radiation exposure restrict their use in routine clinical practice. In contrast, DXA presents an attractive alternative for both research and clinical applications, effectively distinguishing between fat, bone mineral, and lean tissues while exposing patients to minimal radiation.^3^, ^11^, ^708^ Consequently, while CT and MRI are considered gold standards for research, DXA is the preferred method for both research and clinical use. It is important to note that these imaging methods primarily detect tissue wasting but do not assess the risk of developing muscle atrophy.^709^, ^710^

Bioimpedance analysis (BIA) estimates the volume of fat and LBM. This method is cost effective, easy to administer, and reproducible, making it suitable for both ambulatory and bedridden patients. BIA techniques have been studied under standard conditions for over a decade,^711^ with results correlating well with MRI predictions.^712^ Prediction equations have been validated for multiethnic adult populations, and reference values have been established for adult white men and women, including older individuals.^713^, ^714^ However, existing BIA prediction models have limitations in accuracy, as their measurements can be influenced by factors such as body water content, electrolyte balance disorders, and specific disease states like heart and renal failure.

##### Muscle strength

When evaluating muscle strength, handgrip strength and knee flexion/extension tests are commonly used to assess upper and lower limb muscle strength, respectively. Additionally, the peak expiratory flow rate can reflect respiratory muscle strength; however, it is not recommended as an isolated measure of sarcopenia at this time. Among these methods, grip strength is a simple yet effective measure of muscle strength and has been shown to correlate well with leg strength.^2^, ^11^

##### Physical performance

A wide range of tests for assessing physical performance are available, including the Short Physical Performance Battery (SPPB), usual gait speed, the 6‐min walk test, and the stair climb power test.^11^, ^715^ The SPPB, which serves as a composite measure of physical performance, is considered a standard assessment tool for both research and clinical practice.^11^

#### 5.2.2 Circulating cytokines, myokines, and metabolic markers

It is imperative to identify new robust biomarkers that are cost‐effective and readily available for diagnosis and therapy monitoring in clinical settings.^710^ Hormonal factors have been proposed to play a significant role in the development of muscle loss, particularly in cachexia and sarcopenia.^716^, ^717^ Notable examples include leptin,^240^ ghrelin,^718^ and obestatin^719^ all of which are believed to be key players in cancer cachexia. These emerging biomarkers have been investigated in oncologic patients as diagnostic and/or predictive markers, as well as for their impact on patient survival.^720^ Kaplan–Meier analysis demonstrated that patients with low levels of ghrelin and high levels of leptin showed the best survival outcomes, whereas those with high ghrelin levels and low leptin levels had a reduced survival probability.^720^ Additionally, inflammatory cytokines such as IL‐1, IL‐6, and TNF‐α, which are associated with anorexia and weight loss, are used for prognosing cancer cachexia and sarcopenia.^721^, ^722^ Myokines also have the potential to predict muscle wasting. As mentioned earlier, adipokines are another relevant factor, and together, they can serve as predictors for sarcopenia and cachexia. Moreover, serum markers may exhibit intra‐day variations, which could affect research outcomes.^723^ Last, the significance of the differences in these markers between sarcopenic and cachectic patients versus nonsarcopenic and noncachectic patients may depend on the diagnostic criteria employed.^724^ Therefore, further studies with larger and more uniform study groups are warranted in this area.

## 6 THERAPEUTIC INTERVENTIONS FOR SARCOPENIA

**FIGURE 3:**
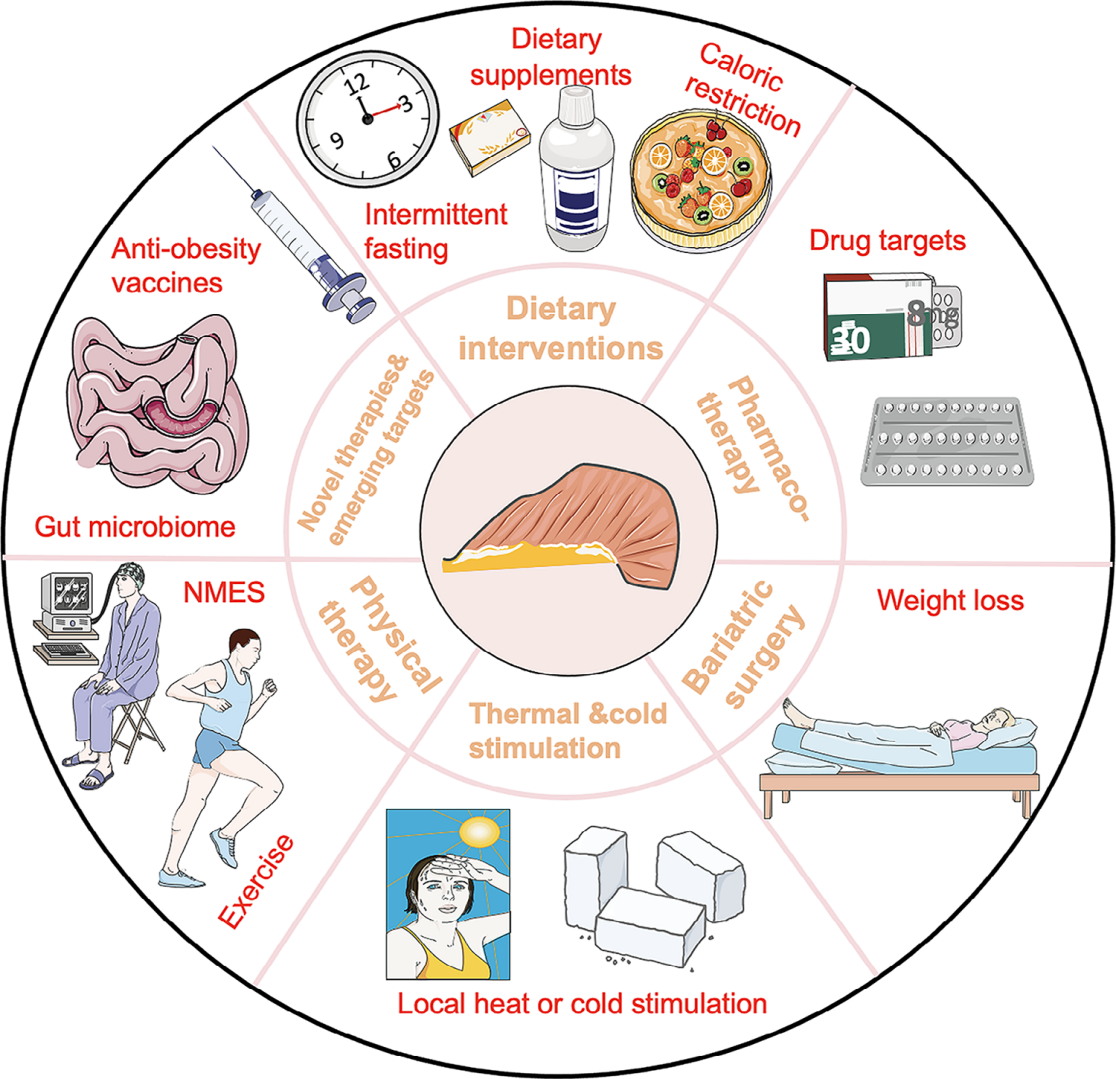
Novel antisarcopenia strategies. The intricate interplay between inflammation and metabolic dysfunctions in adipose tissue and muscle during aging complicates current treatments that target metabolic defects, dysbiosis, inflammation, or senescent cells in isolation, often resulting in limited success in treating sarcopenia. To address these challenges, we propose a comprehensive approach that combines various therapies, including dietary modifications, potential bariatric surgery, pharmacological interventions, and structured physical exercise. However, it may be essential to incorporate localized heat and cold stimulation alongside resistance training to effectively modify gene expression related to proteolysis and myogenesis, as temperature alone might not suffice. While bariatric surgery is an effective intervention for obesity, it can inadvertently increase the risk of sarcopenia due to accompanying muscle loss. Therefore, optimizing exercise protocols and ensuring adequate protein intake immediately postsurgery could be crucial in mitigating muscle decline. Physical training offers promising avenues for treating sarcopenia by addressing senescence and inflammation, promoting the browning of white adipose tissue (WAT), and positively influencing gut microbiota. This integrated strategy, which tackles inflammation, metabolic disturbances, and their repercussions on muscle health, holds significant potential for effectively managing sarcopenia. WAT, white adipose tissue; NMES, neuromuscular electrical stimulation.

The interconnection between aging adipose tissue and declining skeletal muscle is increasingly evident across various pathological conditions, such as sarcopenia, sarcopenic obesity, obesity, and metabolic disorders. This relationship can be illustrated as a detrimental cycle set off by aging, triggering alterations in adipose tissue, which in turn prompts inflammation and muscle impairment. The compromised muscle function further exacerbates inflammation, perpetuating the sarcopenic cycle. To mitigate sarcopenia, preventive strategies encompass dietary adjustments, bariatric surgery, pharmaceutical interventions, and physical exercise, targeting the enhancement of aged adipose tissue and the mitigation of its adverse effects on muscle wellbeing (Figure 3).

### 6.1 Exercise and physical therapy

#### 6.1.1 Resistance training and its benefits on muscle mass and function

In WAT, consistent exercise results in a significant reduction in adipocyte size,^725^ an increase in mitochondrial biogenesis,^726^ control of adipokine release,^727^ resulting in an overall improvement in whole‐body metabolic health.^728^ Additionally, 12‐month treadmill exercise training induces adaptability in WAT. This is evidenced by increased FFA oxidation and a decrease in the inflammatory response, achieved through the regulation of proinflammatory and anti‐inflammatory gene expression, as well as a reduction in macrophage infiltration^729^ In elderly women, the number of p16INK4a‐positive cells in adipose tissue is negatively correlated with exercise capacity and cognitive function. However, combined treatment with physical exercise and calorie restriction can significantly reduce the number of p16INK4a‐positive cells in adipose tissue, promoting overall health.^730^

The majority of studies examine whether exercise primarily generates heat within the body while also reducing body weight. Consistent with these findings, preclinical data have shown that exercise directly influences BAT activity by boosting thermogenic gene expression and promoting WAT browning.^731^ Exercise is also known to diminish age‐related oxidative damage and curb chronic inflammation.^732^ Furthermore, myokines like IL‐6, induced by exercise, play a beneficial role in enhancing BAT activity, promoting browning of WAT, and reducing inflammation.^733^ In particular, mounting evidence has affirmed that irisin may serve as a positive regulator pertinent to this mechanism.^734^ Boström's study revealed that plasma irisin levels increased after mice underwent 3 weeks of free wheel running. Similarly, circulating irisin showed a twofold increase with 10 weeks of endurance exercise training in healthy adults.^527^ Additionally, irisin has demonstrated the ability to induce WAT to adopt a BAT‐like phenotype, leading to heightened energy expenditure, reduced body weight, and enhanced glucose homeostasis, along with improved IR.^527^

Additionally, regular physical exercise also offers benefits for sarcopenia. Resistance training is particularly effective in combating muscle wasting and age‐related sarcopenia^735^ and improving muscle protein anabolism, as well as promoting specific metabolic and morphological muscular adaptations.^736^ Furthermore, multiple studies have affirmed that endurance exercise training leads to enhancements in maximal oxygen consumption, mitochondrial function, and insulin sensitivity, thereby mitigating sarcopenia progression.^737^, ^738^, ^739^, ^740^ Indeed, myokines play a crucial role in exercise's preventive effects against sarcopenia. For instance, irisin has been identified as increasing mitochondrial content and oxygen consumption while safeguarding skeletal muscle against metabolic stresses.^537^, ^538^ According to clinical findings, reduced levels of irisin due to aging could serve as a predictor for sarcopenia,^531^, ^532^ indicating that exercise‐enhanced serum irisin might prevent sarcopenia. Indeed, in support of this notion, studies have demonstrated that resistance training could serve as an effective intervention to increase irisin levels and prevent the age‐related decline in muscle function.^530^

Overall, it is clear that physical exercise is now commonly incorporated as a therapy for sarcopenia, possibly through regulating muscle, BAT and WAT functions as well as decreasing inflammation. How such changes might affect sarcopenia is still poorly understood, but we attempt to offer some insight here, suggesting that myokines are involved in this process. Further studies defining other mechanistic insight into the link among physical exercise, WAT regulation and sarcopenia are needed, especially in humans, and evidence of a direct causal relationship is thus also missing.

#### 6.1.2 Neuromuscular stimulation and rehabilitation programs

Neuromuscular electrical stimulation (NMES) therapy is a physical therapy technique that employs muscle stimulation devices to deliver low‐frequency pulse currents through electrodes, directly targeting nerves or muscle tissue to induce skeletal muscle contraction. The primary aim of this therapy is to promote the recovery of neuromuscular function.^741^ Muscle plasticity in aged subjects undergoing NMES training is well documented, although many underlying mechanisms remain unexplored and warrant further investigation. Chronic application of NMES, when optimized for motor performance, can lead to increases in muscle mass,^742^ strength,^743^ power,^744^ activation,^745^ and endurance.^746^ For example, NMES has been identified as an effective alternative intervention for enhancing lower limb muscle mass and performance.^747^ Currently, NMES is utilized in sports training to boost muscle performance and in rehabilitation medicine to restore muscle properties following injury or surgery.^748^ This suggests that electrotherapy may hold promise for treating sarcopenia with minimal side effects, potentially paving the way for advancements in personalized bioelectronic medicine. For instance, Boutry‐Regard and his team^749^ proposed an innovative approach that combines NMES with nutritional interventions for sarcopenia. This method could help older adults overcome behavioral, physiological, and clinical challenges.

From a behavioral perspective, NMES can compensate for reduced physical activity and motivation to exercise. Clinically, it may help partially restore muscle strength and functional abilities, such as gait and balance, through chronic NMES application^748^ thereby reducing fall risk.^750^ NMES training effectively addresses age‐related mobility issues, frailty, and fall risk while enhancing functional performance and ADL.^751^, ^752^

Overall, physicians, therapists and physical conditioners/trainers could exploit this new knowledge in their professional practice to improve life conditions of frail and/or aged subjects.

### 6.2 Dietary interventions

#### 6.2.1 Intermittent fasting

Fasting, whether it involves partial or total abstention from food or selective avoidance of certain foods, represents a potential nonpharmacological approach. It induces a metabolic shift from relying on glycogen for energy to utilizing fat stores, thereby effectively regulating body weight and reducing body fat levels.^753^, ^754^ Moreover, fasting has been shown to enhance insulin sensitivity and glucose tolerance, diminish inflammatory responses, and mitigate oxidative stress. These effects contribute to the effective prevention and postponement of metabolic disorders like type II diabetes, myopathy, and cancer, ultimately promoting health and slowing down the aging process.^753^, ^754^ Intermittent fasting (IF) refers to the regular maintenance of zero or very low‐calorie intake in a specific time, that is, fasting and free feeding altern IF involves regularly alternating between periods of zero or very low calorie intake and periods of unrestricted eating. Research suggests that IF can extend a healthy lifespan and may help in managing diseases associated with aging.^755^ Currently, research on the mechanisms underlying IF primarily focuses on factors such as IR, oxidative stress, and autophagy.^756^, ^757^, ^758^ Recent studies have demonstrated that fundamental autophagy processes are crucial for preserving muscle mass, as well as maintaining the integrity and adaptability of muscle fibers.^759^ An excessive activation of autophagy or its inhibition can result in muscle atrophy and potentially contribute to conditions like myofibrosis and muscle weakness.^760^ Furthermore, IF, as a dietary intervention, has been utilized in humans to address issues such as obesity and metabolic syndrome.^761^, ^762^ Indeed, there is limited research on how IF influences myocyte renewal. Given its potential to systematically enhance autophagy and the significant role of autophagy in tissue and cell renewal, future investigations should delve into whether IF affects muscle cell renewal and whether it impacts sarcopenia through autophagy mechanisms.

#### 6.2.2 Caloric restriction

Caloric restriction (CR) is indeed a dietary intervention known to delay aging and extend the healthy lifespan across various species.^763^ As aging progresses in mice, several changes occur including reduced potential for WAT browning, enlargement of adipocytes, decreased β‐oxidational function, and diminished thermogenic activity in BAT. Continuous CR through low calorie intake has been shown to decrease total fat mass and body weight, potentially through increased WAT browning. Additionally, small adipocytes following CR tend to secrete fewer proinflammatory cytokines like leptin and more adiponectin, resulting in reduced adiposity and improved metabolic outcomes.^764^ Indeed, during CR, there is an increase in M2 macrophage infiltration and eosinophils, along with enhanced type 2 cytokine signaling in fat tissues. These factors contribute to the development of functional beige fat induced by CR.^765^ Caloric excess, lack of exercise, and obesity can indeed disrupt long‐term energy metabolism balance, potentially resulting in increased production of ROS and reduced mitochondrial density in skeletal muscle.^766^

Initially, CR demonstrates a protective effect against muscular atrophy in rodents and primate mammals. After short‐term CR of 35 or 50% in 17‐month‐old rats, an increase in the number of satellite cells and the content of collagen VI was observed.^767^ Absolutely, muscle function depends not only on the structural integrity of the muscle tissue but also on the consumption of ATP to provide energy.^768^ Mitochondria play a central role in oxidative phosphorylation and ATP synthesis, serving as primary sites for energy production within cells. Furthermore, CR is employed to safeguard mitochondria and enhance proteolysis pathways, crucial for preserving the integrity of protein turnover mechanisms and maintaining skeletal muscle function.^769^ Previous studies have shown that CR reduces proton leakage and ROS generation in mitochondria within skeletal muscle, while also enhancing the expression of genes associated with ROS scavenging. Moreover, CR may alter the fatty acid composition of the mitochondrial membrane, thereby reducing lipid oxidation and proton leakage.^770^ Growing evidence suggests that apoptosis could play a fundamental role in both the onset and progression of sarcopenia.^771^ It is been proposed that CR may reduce the expression of various proapoptotic regulatory proteins, thereby diminishing the age‐related apoptotic potential.^772^ Specifically, in CR mice compared with control mice, there was a significant decrease observed in both the gene expression and cleavage of precaspase‐3 in the gastrocnemius muscle.^773^ CR has been demonstrated to be effective in preventing sarcopenic muscle loss in animals, which leads to a reduction in oxidative stress levels.^774^ CR has the potential to delay the onset of sarcopenia; however, it is essential to note its adverse effects in young mice (3 weeks old), including impaired skeletal growth, negative impacts on bone architecture and mass, and an increase in marrow adiposity.^774^ It is important to highlight that factor such as nutrition levels, dietary macronutrient composition, timing of interventions, and the use of different animal strains can significantly influence the observed effects of CR. These variations may contribute to the ongoing debate surrounding the efficacy of CR in delaying sarcopenia.

In the future, it will be essential to explore strategies for reconciling the apparent increase in brown adipocyte thermogenesis with the reduced energy expenditure (metabolic adaptation) that occurs during CR. Targeting WAT browning could emerge as a promising approach for promoting metabolic health during aging.

#### 6.2.3 Dietary supplements

##### Dietary fats

Research indicates that dietary lipids play a dual role by not only providing energy but also regulating various cellular functions. Among dietary fatty acids, essential, polyunsaturated, saturated, and monounsaturated fats are key classifications. Moreover, recent findings suggest that consuming high levels of plant‐based essential fatty acids (ePUFAs) such as n‐6 linoleic acid (LA) and n‐3 α‐linolenic acid (ALA) may exert a more profound influence on body composition (including fat mass and lean mass) and glucose homeostasis compared with marine‐derived long‐chain n‐3 polyunsaturated fatty acids (PUFAs) like eicosapentaenoic acid (EPA) and docosahexaenoic acid (DHA).^775^ Furthermore, a high intake of both ePUFAs (LA and ALA) might exhibit anti‐inflammatory effects in humans. For example, a systemic review conducted by Bender et al.,^776^ which suggested that while supplementation of long‐chain n‐3 PUFA could modestly influence weight loss in the short term (<2 months), there was no evidence of associations with longer‐term intake. Further reinforcement for this argument can be found in a recent placebo‐controlled prospective RCT involving adults with overweight or obesity. The study indicated that 6 months of high‐dose marine n‐3 PUFA supplementation (3.9 g/day of EPA and DHA; 4.2 g total n‐3 per day) compared with placebo (4.2 g/day oleate) had no beneficial effects on adipose tissue lipolysis or inflammation.^777^ Indeed, a pooled analysis involving 20 prospective cohort studies conducted globally revealed that high levels of the biomarker for LA, measured in various forms such as adipose tissue, cholesterol ester, erythrocytes, or plasma phospholipids, and/or total plasma, were associated with dose‐dependent decreases in the incidence of T2DM.^778^ These findings are consistent with earlier cross‐sectional and intervention studies, which have shown that biomarkers of LA and ALA intake, rather than marine‐derived n‐3 PUFA, are linked to better glycemic control and/or insulin sensitivity, as well as a reduced risk of T2DM.^779^ Belury et al.^780^ found that erythrocyte LA (a biomarker of LA status) was positively associated with appendicular lean mass adjusted for BMI. Conversely, it was inversely related to trunk adipose mass, as well as with the homeostatic model assessment of IR and inflammation markers such as IL‐6.^780^ Research has demonstrated that compared with food‐restricted rats refed a high‐fat (lard) diet low in PUFA, those refed isoenergetically on diets enriched in LA or ALA, irrespective of the n‐6:n‐3 ratio, exhibit improved insulin sensitivity. Additionally, they tend to have lower fat mass and higher lean mass.^781^

Indeed, the benefits of ePUFA, particularly LA and ALA, as outlined, suggest that increasing ePUFA intake could have significant implications for public health and clinical medicine. These implications extend beyond the prevention of obesity to include the management of musculoskeletal and metabolic health throughout the lifespan. Given the observed positive associations with lean mass, improved insulin sensitivity, and reduced adiposity, promoting adequate ePUFA intake may be a valuable strategy for enhancing sarcopenia and overall health.

##### Resveratrol

Resveratrol, a prominent natural compound, is present in several plants, especially in the skin of red grapes and red wine. Its potential health benefits have gained significant attention, and it has traditionally been used in Chinese and Japanese medicine for its anti‐inflammatory and antiplatelet effects.^782^ Its anti‐inflammatory and antioxidant properties suggest that resveratrol may help prevent the development of diabetes and obesity.^783^ Aguirre et al.^784^ demonstrated that resveratrol can inhibit the early regulatory factor of adipogenesis, C/EBPβ, and effectively suppress adipogenesis in porcine, murine, and human preadipocytes at concentrations between 20 and 100 µM. Additionally, resveratrol exhibits lipolytic effects only at higher concentrations (50‐100 µM), but it can enhance the lipolytic effect of β‐adrenergic stimulation at any concentration.^784^ Research has shown that resveratrol supplementation can elevate levels of long‐chain and polyunsaturated fatty acids while reducing steroid levels. It may also decrease the size of abdominal subcutaneous adipocytes.^785^

Huang et al.^786^ discovered that resveratrol supplementation can prevent the buildup of intramuscular fat and increased body fat mass associated with a HFD, thereby helping to mitigate muscle loss. Resveratrol may enhance oxidative stress and mitochondrial function through the PKA/LKB1/AMPK pathway, which helps prevent HFD‐induced muscle atrophy in older rats. As a result, resveratrol holds potential for preventing sarcopenic obesity.^786^, ^787^ Resveratrol may increase mitochondriogenesis and elevate UCP3 protein levels in skeletal muscle. Additionally, the upregulation of genes involved in mitochondrial oxidative phosphorylation suggests that resveratrol could improve the oxidative capacity of muscle fat.^788^ Reports indicate that resveratrol may boost exercise‐related nuclear and satellite cell numbers and enhance the fiber cross‐sectional area of exercise‐responsive muscle, potentially leading to improvements in muscle function.^90^ Human studies on resveratrol's effects on sarcopenia, including RCTs, have been conducted. One trial with 30 healthy adults administered either a placebo or 500 mg/day of resveratrol for 12 weeks before and after exercise. Results showed that those receiving resveratrol with exercise experienced significant improvements compared with the placebo group, including better mitochondrial density, reduced muscle fatigue, increased mean muscle fiber area, and more total myonuclei. Additionally, the resveratrol group demonstrated enhanced knee extensor muscle peak energy, average peak energy, and muscle strength postexercise, while these parameters did not show significant improvement in the placebo group.^90^ A recent study by Toniolo et al.^789^ found that a 6‐month dietary supplementation of 0.04% resveratrol significantly improved muscle fatigue resistance. The study indicated that resveratrol enhanced capillarization in the skeletal muscle of aging mice, suggesting its potential to prevent capillary rarefaction in muscle tissue.^789^

While resveratrol appears promising in preventing sarcopenia by boosting muscle function and resistance to fatigue, some long‐term studies have found only modest or no significant improvements in muscle mass or inflammation.^90^, ^790^ These findings highlight the complexity of studying dietary supplements like resveratrol and their effects on muscle health. They underscore the need for further research to better understand how resveratrol functions and its potential benefits, especially in relation to sarcopenia and muscle inflammation.

### 6.3 Pharmacotherapy

#### 6.3.1 Hormone replacement therapy

As people age, circulating levels of various anabolic hormones decline, potentially leading to changes in muscle mass and function. Consequently, hormone modulation has been explored as a foundational strategy for treating sarcopenia, with testosterone supplementation being the most commonly studied option for enhancing muscle mass and promoting muscle‐protein anabolism.^791^, ^792^ However, the findings from studies on testosterone replacement therapy in men vary based on factors such as age, pretreatment testosterone levels, and administration methods. These variables complicate the assessment of therapy's impact on physical performance and disability. Additionally, potential side effects—such as peripheral edema, gynecomastia, polycythemia, and sleep apnea—must be weighed against the therapeutic benefits. A significant concern is that elevated testosterone levels may increase the risk of prostate cancer, necessitating careful planning and monitoring during treatment.^792^

Recently, selective androgen receptor modulators (SARMs) have emerged as a safer and more attractive alternative to long‐term testosterone therapy for combating muscle wasting. SARMs are nonsteroidal compounds that selectively bind to androgen receptors (AR), providing tissue‐selective anabolic effects similar to testosterone but with fewer side effects. These compounds also show promise for women due to their specific activity and improved pharmacokinetics.^793^, ^794^ Other hormone modulation therapies also hold potential benefits. Dehydroepiandrosterone may enhance muscle mass and strength in both men and women, while tibolone can promote muscle anabolism by binding to ARs in muscle fibers and increasing levels of serum‐free testosterone, growth hormone, and IGF‐1. Estrogen may help suppress inflammatory cytokines and improve muscle strength in women, while growth hormone can stimulate the proliferation of muscle satellite cells and enhance muscle function. Ghrelin has also been shown to increase lean mass and physical performance.^795^ Despite the emerging evidence linking age‐related hormonal changes to sarcopenia, the clinical efficacy of hormone supplementation for treating this condition requires further investigation and validation in future studies.^791^

#### 6.3.2 Metformin

Metformin's widespread use as an oral antidiabetic drug is well established. Research indicates that metformin enhances proteins associated with mitochondrial biogenesis and VLDL clearance in BAT in mice, suggesting a direct effect on BAT. In human studies, metformin has been shown to reduce cellular respiration in brown adipocytes and effectively counteract obesity‐induced suppression of brown adipogenesis and thermogenesis. These findings suggest that BAT may be a target organ for metformin's actions.^796^ Metformin's influence on fatty acid metabolism offers a promising approach for lifespan extension. Additionally, its ability to reduce collagen gene expression suggests it might help mitigate ECM deposition related to aging, potentially improving adipose tissue regulation often disrupted by aging.^797^ Collagen buildup is linked to age‐related muscle loss, as excessive ECM deposition in aging skeletal muscles can stiffen myofibers, affecting myogenic progenitor cells and contributing to muscle aging. Metformin's potential to reduce collagen gene expression and ECM deposition suggests it may help treat sarcopenia by addressing these age‐related changes in muscle tissue.^798^

Overall, metformin has been shown to guard against sarcopenia by influencing BAT and ECM dynamics, promoting the differentiation of skeletal muscle cells, and aiding in the maturation of myotubes, which enhances overall skeletal muscle function.^799^ Clinical trial data in elderly individuals without T2DM suggest that metformin may positively impact sarcopenia. While epidemiological studies indicate potential benefits, clinical trial results have shown varying consistency. In one study, metformin use led to a notable improvement in mean walking time by 0.39 s and a corresponding increase in gait speed of 0.13 m/s, highlighting its potential positive effects on physical performance in the context of sarcopenia.^800^ These findings indicate that metformin might enhance physical function and mobility in elderly individuals, even without T2DM. However, in a prospective open‐label observational study of overweight patients with both T2DM and chronic obstructive pulmonary disease, administration of metformin (850 mg twice daily) for 24 weeks resulted in a significant decrease in handgrip strength by 3.2%.^801^ Further clinical research is essential to determine the effectiveness of metformin in treating sarcopenia.

#### 6.3.3 TRPM8 channel

Activating transient receptor potential (TRP) channels, like TRPM8, is an alternative method to indirectly stimulate BAT thermogenesis. TRPM8, a calcium‐gated channel responsive to heat and cold, functions as a key cold‐sensing molecular transducer in the peripheral nervous system of mice.^802^ Administering menthol activates TRPM8, leading to increased UCP‐1 expression and thermogenesis, which raises body temperature and provides protection against obesity in both mice and humans. Additionally, menthol intake enhances exercise endurance and energy metabolism by upregulating PGC‐1α in skeletal muscles through TRPM8 activation,^803^ improving muscle function. Therefore, targeting the TRPM8 channel could potentially be a viable intervention for improving both obesity management and skeletal muscle function.

#### 6.3.4 βARs

In rodents, BAT thermogenesis is activated by sympathetic nervous system (SNS) input, with substantial evidence highlighting the critical role of β3‐AR in this process.^804^, ^805^, ^806^ Specifically, β3‐AR stimulation increased the gene expression of UCP1, linked to thermogenesis, as well as Cpt1b and Acox1, which are involved in β‐oxidation.^807^ Notably, β3‐AR mRNA levels in human BAT are lower than those in rodents, which makes the role of β3‐AR in humans less certain than in rodents.^808^ Blondin et al.^809^ discovered that β2‐AR mediates lipolysis and thermogenesis in BAT in response to sympathetic nervous stimulation in humans. As a result, the primary adrenergic receptor subtype responsible for regulating BAT thermogenesis in humans remains debated.

Currently, β2‐AR agonists show promise for stimulating skeletal muscle hypertrophy by boosting protein synthesis.^810^ β2‐AR agonists induce a significant shift in skeletal muscle fiber types from slow‐oxidative to fast‐glycolytic.^811^, ^812^ This shift may partly explain their beneficial effects in sarcopenia,^813^ as age‐related muscle loss predominantly affects fast‐twitch fibers.^814^, ^815^, ^816^ A small clinical trial with patients who have brachial plexus injuries supports the effectiveness of β2‐AR agonists in reducing muscle mass loss due to denervation.^817^ Moreover, β2‐AR stimulation can accelerate skeletal muscle recovery following myotoxic injury, prompting the use of synthetic β2‐AR agonists to counteract or even reverse muscle weakness and wasting associated with aging and disease.^814^, ^818^, ^819^ Besides, activation of the β2‐AR–cAMP–PKA–mTORC2 pathway has been demonstrated to promote GLUT4 translocation to the plasma membrane and enhance glucose uptake independently of insulin.^820^ Additionally, β3‐AR agonists can also benefit muscle health. For example, Kern et al.^821^ treated obese individuals with IR using the β3‐AR agonist mirabegron (50 mg/day for 12 weeks), which resulted in an increase in type I muscle fibers and favorable changes in skeletal muscle gene expression, likely due to enhanced PGC‐1α expression. Previous research has demonstrated that PGC‐1α regulates genes related to mitochondrial function in various tissues, including the brain, heart, and skeletal muscle.^822^

Given that βARs influence adipose and muscle signaling differently across various pathological conditions, a deeper understanding of how βAR activation improves adipose and muscle function could help develop drugs that selectively target βAR signaling, potentially offering new treatment options for age‐related conditions like sarcopenia and metabolic diseases.

#### 6.3.5 DPP4/GLP‐1R

It is worth noting that other treatments, such as glucagon‐like peptide 1 receptor (GLP‐1R) agonists and dipeptidyl peptidase‐4 inhibitors (DPP4i), have also been suggested as potentially beneficial for enhancing BAT activity.^823^, ^824^ The introduction of GLP‐1R agonists and DPP4i has markedly changed the perspective on how T2DM impacts the regulation of musculoskeletal diseases.^825^, ^826^, ^827^ Exendin‐4, a GLP‐1R analog, may increase BAT activity, leading to faster plasma clearance of triacylglycerol and glucose, reduced food intake, and decreased body fat content, which can ultimately result in weight loss.^828^ Sitagliptin, a DPP4i, has been shown to increase energy expenditure by enhancing BAT activity involved in energy combustion.

Additionally, Nahon et al.^829^ demonstrated that sitagliptin can boost mitochondrial gene expression in skeletal muscle, enhancing muscle metabolism by upregulating PGC‐1β. Ogawa's research^830^ found that elderly diabetic patients treated with a DPP4i had a significantly reduced risk of losing appendicular skeletal muscle mass, indicating a protective effect on muscle mass, particularly in the lower limbs. Epidemiological evidence suggests that elderly diabetic patients on DPP4i exhibit better sarcopenia parameters compared with those treated with other glucose‐lowering medications.^831^ Indeed, inconsistencies exist in the results. For example, a study found no significant changes in skeletal muscle mass and skeletal muscle index after 24 weeks of treatment with the DPP4i teneligliptin (20 mg daily) in overweight or obese patients with T2DM.

In contrast, treatment with the GLP‐1R agonist dulaglutide led to reductions in both markers of sarcopenia.^832^ Liraglutide treatment (GLP‐1R agonists, 3.0 mg daily) with over twenty‐four weeks is well tolerated and leads to fat mass reduction, particularly in android fat, while also improving the skeletal muscle index in patients with T2DM.^833^ Although the data are limited, they indicate that DPP4i, in addition to their established efficacy and safety for glucose control in elderly patients with T2DM, might be a favorable choice for managing sarcopenia risk due to their neutral effect on muscle mass.^834^ Based on these data, DPP‐4/GLP‐1R‐targeting medications may hold promise as potential treatments for sarcopenia. However, additional research is required to fully understand their efficacy and safety in addressing the condition.

#### 6.3.6 Clearance of senescent cells

Senescent cells are more prevalent in older individuals than in younger ones, indicating that these cells accumulate in adipose tissues and muscle with age. This is supported by elevated levels of markers such as p16, p53, and SAβ‐gal found in the adipose tissue of elderly individuals with obesity.^835^, ^836^ In people with obesity, senescence affects not only adipocytes but also extends to endothelial cells and preadipocytes within adipose tissue.^837^, ^838^ In adipose tissue, cellular senescence leads to immune cell infiltration, which creates a proinflammatory environment for preadipocytes. Senescent preadipocytes produce a SASP, and activated macrophages secrete chemokines and cytokines, exacerbating the inflammation associated with aging in adipose tissue.^130^, ^839^ The increased release of proinflammatory cytokines and chemokines by senescent cells in aging individuals is associated with age‐related metabolic dysfunctions.^130^, ^840^, ^841^

Studies have shown that senescent cells and the SASP can contribute to the progression of age‐related diseases such as osteoporosis, OA, and sarcopenia.^50^, ^826^, ^842^, ^843^ Consequently, removing senescent cells has been shown to delay the aging phenotype in both human and mouse cells.^842^, ^844^ Inducible depletion of p16INK4a cells in the BubR1 preaging mouse model led to delayed tissue dysfunction in adipose tissue, skeletal muscle, and the eye.^844^ In sarcopenia, there is a complex interplay between cellular senescence, SASP, and muscle dysfunction. Research by Jejurikar et al.,^87^ for example, shows that aged MuSCs are more prone to senescence or apoptosis than younger ones. Indeed, SASP factors have been linked to the upregulation of molecules like sarcolipin, which impairs myogenic differentiation and promotes skeletal muscle fibrosis. This contributes significantly to the development of sarcopenia.^845^ Transplanting a small number of senescent cells into young and aged mice caused decreased muscle strength and physical impairment. Existing evidence shows that eliminating senescent cells significantly mitigates sarcopenia and enhances muscle regeneration activity in aging and senescence mouse models.^835^, ^844^ For example, treatment with Dasatinib and quercetin (D+Q) has been linked to lower levels of senescent cells and reduced production of proinflammatory cytokines, which may help alleviate physical dysfunction.^143^ Silencing p16INK4a cells may help preserve the regenerative capacity of satellite cells. Conversely, abnormal p16INK4a expression has been shown to obstruct the proliferation of satellite cells in injured young muscle within aged mice.^846^ In the future, clinical trials should investigate the potential of drugs that target and clear senescent cells as a means to alleviate sarcopenia.

### 6.4 Bariatric surgery

Bariatric surgery is currently recognized as the most effective approach for treating obesity. It not only leads to significant weight loss but also enhances insulin sensitivity and overall metabolic function in the body.^847^ Common surgical options include vertical sleeve gastrectomy (VSG), Roux‐en‐Y gastric bypass (RYGB), laparoscopic adjustable gastric banding, and biliopancreatic diversion with duodenal switch, which is less frequently performed.^848^ Following bariatric surgery, it is common to observe a significant reduction in adipose tissue mass, with up to half of the total adipose tissue being lost as the most notable effect.^847^ Furthermore, bariatric surgery has been shown to be effective in managing T2DM and hypertension, leading to reduced incidence rates and mortality associated with cardiovascular events.^849^ Obesity typically leads to decreased levels of adiponectin, which can be increased through metabolic surgery. Additionally, RYGB has been shown to decrease leptin levels, which is associated with both CR and weight loss.^848^ Research indicates that bariatric surgery can lead to significant weight loss in women due to continued mobilization of adipose tissue from intermuscular tissue and vWAT for up to 2 years postsurgery. However, between the 12th and 24th months after surgery, males tend to experience a rebound in weight gain across all adipose tissue depots.^850^

Studies have shown that bariatric surgery can result in a reduction of up to 32% of preoperative weight within two years after the procedure. This weight loss encompasses both fat mass and muscle mass, potentially leading to a decrease in basal metabolism, functional impairment, and a decline in overall QOL.^851^ Voican et al.^852^ reported that the prevalence of sarcopenia could increase significantly after bariatric surgery, rising from 8% at the time of surgery to one‐third of patients one year after undergoing VSG. Indeed, bariatric surgery can potentially elevate the risk of sarcopenia due to the loss of muscle mass. Optimizing exercise guidelines and ensuring adequate protein intake during the initial months following surgery could prove effective in mitigating muscle mass loss after undergoing bariatric surgery.^851^

### 6.5 Thermal and cold stimulation

BAT is primarily sensitive to thermal stimulation via the SNS.^853^ Acute heat stress can directly enhance the lipolytic response of bovine primary adipocytes by increasing protein kinase A phosphorylation of hormone‐sensitive lipase and perilipin. However, the reduced lipolytic response observed during heat stress in vivo might result from increased β‐adrenergic receptor downregulation or A1 receptor activation, attributable to the heightened sensitivity to β‐adrenergic receptor agonists.^854^ The application of localized heat can reduce FOXO3 mRNA levels, resulting in a decrease in proteolytic activity. Additionally, skeletal muscle heating may enhance the myogenic response to resistance exercise.^855^ Indeed, local muscle cooling combined with resistance training could induce proteolysis, thereby potentially resulting in a decrease in muscle growth.^855^

Cold stimulation can increase SNS activity, promoting BAT glucose uptake and lipolysis, consequently generating heat in BAT. Furthermore, SNS activation may decrease pancreatic insulin secretion while increasing lipolysis and glycolysis, thus elevating circulating fatty acids and glucose for uptake by brown adipocytes.^853^ Hence, cold exposure or activation of adrenergic signaling could serve as a potential complementary or adjunct therapeutic approach to prevent obesity development or the progression of T2DM.^856^ Zak et al.’s^857^ report indicated that environmental temperature alone could not modify myogenetic or proteolytic expression. Indeed, Ivanova and Blondin^856^ reported that cold exposure could enhance insulin sensitivity, glucose homeostasis, and the oxidation rate of fatty acids by stimulating thermogenic processes, particularly the recruitment of shivering skeletal muscles, indicating the beneficial effects of cold stimulation. Initiating the senescence pathway in young beige progenitors can trigger premature cellular senescence, hindering their capacity to produce cold‐induced beige adipocytes. Conversely, reversing cellular aging genetically or pharmacologically, notably through the p38/MAPK‐ p16INK4a pathway, in aged mouse or human beige progenitor cells rejuvenates cold‐induced beiging.^858^ Additionally, these treatments may have limited applications due to associated cardiovascular risks.^859^, ^860^

It appears that temperature alone might not be adequate to induce changes in the gene expression of proteolysis and myogenesis. Therefore, combining local heat and cold stimulation with resistance exercise could be necessary to elicit significant gene expression changes.^857^ In the future, researchers should investigate how hot and cold stimulation impacts skeletal muscle function.

### 6.6 Combined therapy

As highlighted in the review, complex mechanisms are significant factors in sarcopenia's development. To address these issues, a comprehensive approach is proposed, including dietary changes, bariatric surgery, pharmaceutical treatments, and physical exercise. Integrating these strategies could potentially slow the progression of sarcopenia by targeting the underlying mechanisms.^861^ This combined therapy approach appears promising for managing sarcopenia, as depicted in Figure 3. Moreover, while various drugs are being developed to target dysfunction muscle in aging individuals, additional research is necessary to discover new drug targets and therapies for addressing sarcopenia and related metabolic disorders.

## 7 THERAPEUTIC INTERVENTIONS FOR CACHEXIA

### 7.1 Anti‐inflammatory therapies

In recent years, as summarized above, clinical studies have implicated proinflammatory cytokines in the pathogenesis of cachexia, particularly cancer‐associated cachexia. Consequently, targeting these cytokines may prove effective in managing cachexia. For instance, in various cachectic mouse models, IL‐6/gp130‐dependent activation of STAT3 has been observed in atrophic skeletal muscle tissue. Pharmacological blockade of the JAK/STAT3 pathway has been shown to reduce muscle atrophy in C26‐driven cachexia, suggesting a significant role for this pathway in IL‐6/gp130‐mediated cachexia.^332^, ^333^, ^862^ Normalization of STAT3 activation levels through either homozygous or heterozygous genetic ablation of IL6 or STAT3 in mice has demonstrated protection against severe muscle wasting and weight loss, thereby improving survival.^863^, ^864^ Additionally, a limited number of advanced lung cancer patients treated with neutralizing antibodies against IL‐6 or its receptor (IL‐6R) experienced amelioration of cachexia‐related symptoms.^865^, ^866^ In cachectic models, pharmacological blockade of the JAK2/STAT3 pathway protected mice from weight loss, independent of tumor growth suppression.^867^Consistent with clinical findings, treatment using a humanized anti‐IL‐6 monoclonal antibody to globally block IL‐6 has shown protection against loss of LBM—a key measure of cachexia—without affecting tumor progression. Similarly, a case study involving the treatment of a cachectic advanced lung cancer patient with the anti‐IL‐6R monoclonal antibody tocilizumab resulted in significant weight gain, yet tumor growth continued unabated, leading to subsequent metastases.^865^The limited efficacy of global IL‐6 blockade in alleviating cancer cachexia symptoms without halting tumor progression is further supported by observations from the LLC cachexia model. Treatment with the monoclonal antibody MR16‐1, a rodent analogue of tocilizumab, preferentially protected against weight loss without affecting tumor growth.^868^ Moreover, a Phase II trial evaluating the safety, efficacy, and pharmacokinetics of the chimeric anti‐IL‐6 monoclonal antibody siltuximab in patients with advanced solid tumors, including non‐small cell lung cancer and Kras mutant colon cancer, revealed that global targeting of IL‐6 was well tolerated but did not impact tumor progression.^869^

TNF‐α is a crucial mediator of cachexia. In animal models, therapies aimed at neutralizing TNF‐α activity have shown significant anticachectic effects.^870^, ^871^, ^872^ For instance, gene knockout mice lacking the TNF‐α receptor type I exhibited less muscle wasting compared with wild‐type mice in a fast‐growing mouse tumor‐induced cachexia model. This underscores the role of TNF‐α in skeletal muscle depletion associated with cancer cachexia.^873^ Additionally, direct inhibition of NF‐κB has been shown to block cachexia in animal studies, suggesting a connection between NF‐κB and TNF‐α in this condition. However, evidence supporting the efficacy of anti‐TNF‐α therapy for treating cachexia in humans remains limited. Factors such as low patient accrual, small sample sizes, and the use of drugs that target only one of the multiple cytokines involved in cancer cachexia development may explain the lack of positive outcomes in these studies.

The strategy of using agents that target cytokines implicated in the pathogenesis of cancer cachexia has gained attention for managing cachexia. Unfortunately, most clinical trials assessing anticytokine therapies for cancer cachexia have not yielded favorable results. To enhance outcomes and address methodological challenges, future cancer cachexia clinical trials should incorporate standardized inclusion criteria, meaningful outcome measures, and low‐toxicity therapies that target multiple procachectic molecules and pathways, especially in the earlier phases of the cancer cachexia continuum. This approach may allow anticytokine treatments to be integrated into a multimodal strategy for managing the complex syndrome of cancer cachexia.

### 7.2 Nutritional interventions

#### 7.2.1 Specialized nutritional formulations to counteract hypercatabolism

Multiple interventions can be considered for patients diagnosed with malnutrition, including dietary counseling, oral nutrition supplements, and enteral or parenteral feeding. Recommendations should be tailored to the patient's specific disease and performance status. Early assessment of nutritional needs can have a lasting impact throughout the treatment duration. Immunonutrition—dietary supplementation with specific nutrients such as fat‐soluble vitamins, omega‐3 fatty acids, arginine, and glutamine—represents a novel strategy to modulate negative immune‐mediated inflammatory responses in advanced cancer and alleviate the physiological stress of surgery.^874^ Omega‐3 fatty acid intake may lead to significant increases in body weight and LBM, reductions in REE, and potentially prolonged survival.^875^ Nutritional formulations containing branched‐chain amino acids, arginine, and glutamine may help reduce cancer cachexia.^876^ However, no specific diet has been definitively proven beneficial in managing cancer cachexia or sarcopenia, partly due to the challenges associated with studying dietary interventions.^877^, ^878^ A recent publication by the American Society of Clinical Oncology reported insufficient evidence to suggest that specific diets—such as low‐fat, high‐fiber, or ketogenic—impact oncologic outcomes, treatment toxicity, or QOL.^879^

#### 7.2.2 Appetite stimulants

Many studies have also been conducted on the use of various appetite‐modulating drugs in cancer‐associated muscle cachexia. One appetite‐modulating approach is medroxyprogesterone acetate, a progesterone derivative similar to megestrol acetate (MA) that has been shown to improve anorexia, body weight, and QOL in phase II clinical trials.^880^ Endocannabinoids such as delta‐9‐tetrahydrocannabinol and nabilone have also been tested as appetite stimulants in phase II clinical trials, and results have generally shown increased appetite and QOL with no major side effects.^881^, ^882^ Other appetite stimulants include corticosteroids such as dexamethasone and prednisolone, which are among the earliest drugs evaluated for cancer cachexia. In a randomized clinical trial, dexamethasone increased appetite but did not affect body weight due to its catabolic effects on muscle and bone.^883^ One preliminary study showed dexamethasone treatment significantly improved patient‐reported cluster scores for fatigue/anorexia–cachexia/depression symptoms after two weeks in advanced cancer patients.^884^ Another novel immunomodulatory, OHR118, affects the synthesis of chemokines and cytokines, including TNF‐α. In a phase 2 study, OHR118 significantly improved appetite, dyspepsia, and depression in cancer patients.^885^

### 7.3 Pharmacological treatments

#### 7.3.1 Ghrelin agonists and anabolic steroids

Ghrelin is an endogenous hormone secreted by the stomach that stimulates appetite and plays a role in regulating energy expenditure by inhibiting sympathetic stimulation of BAT. It is believed to alleviate some symptoms of cancer cachexia by inhibiting MuRF‐1/MAFbx‐induced protein degradation in muscle tissue, promoting muscle protein synthesis via IGF‐1, and increasing fat accumulation.^886^ Due to its significant effects on appetite and metabolism, ghrelin has been the focus of several recent clinical trials. Anamorelin HCl, a ghrelin analogue with an extended half‐life, has undergone extensive testing in phase I, II, and III trials, demonstrating good tolerability and effectiveness in improving weight gain, lean muscle mass, and anorexic/cachectic symptoms in cachectic cancer patients.^887^, ^888^, ^889^, ^890^ However, it did not show significant improvement in handgrip strength during phase III trials.^889^, ^890^ Additionally, the potential side effects of anamorelin must be considered, as agents that elevate IGF‐1 or growth hormone levels can lead to diabetes or IR.^891^ Anabolic steroids have also been used effectively to treat muscle wasting,^892^, ^893^ particularly in conditions like chronic heart failure, where nearly 20% of patients experience this issue.^894^ In heart failure patients, low levels of circulating anabolic hormones are linked to poor outcomes.^895^, ^896^ However, the risks associated with anabolic steroid administration often outweigh their potential benefits.

#### 7.3.2 Beta‐blockers to modulate catabolic signals

β‐Blockers can reduce body energy expenditure and enhance substrate utilization efficiency, with some exhibiting multiple pharmacological effects. Espindolol (MT‐102; PsiOxus Therapeutics) is a nonspecific β1/β2‐adrenergic receptor antagonist that also acts on β and central 5‐HT1α receptors, demonstrating proanabolic, anticatabolic, and appetite‐stimulating properties.^21^ The ACT‐ONE trial (NCT01238107) found that espindolol, administered at 10 mg twice daily, effectively reversed weight loss, improved fat‐free mass, maintained fat mass, and enhanced handgrip strength in cachectic patients with non‐small cell lung cancer or colorectal cancer.^897^, ^898^

### 7.4 Multimodal interventions

#### 7.4.1 Combination therapy

Due to the complexity of cancer cachexia syndrome, multidrug combination therapy targeting multiple underlying pathophysiological processes has been proposed. In a phase III RCT conducted in 2010, 332 patients with cachexia associated with cancer were randomized into five treatment arms: progesterone analogs, EPA, L‐carnitine, thalidomide, or a combination of all four interventions. The combination arm showed significant improvements in LBM, REE, fatigue, appetite, IL‐6 levels, Glasgow prognostic scores, and Eastern Cooperative Oncology Group performance scores.^899^

Conversely, a 2012 report indicated that a combination of l‐carnitine, celecoxib, and MA did not provide greater benefits than the two‐drug combination of l‐carnitine and celecoxib alone, as both groups demonstrated significant increases in LBM and physical performance compared with baseline.^899^ These findings suggest that additional treatments do not always enhance efficacy. Furthermore, the combination of olanzapine and MA was assessed in a 2010 RCT involving patients with advanced lung or gastrointestinal cancer, revealing significantly greater weight gain in the combination group compared with the MA‐only group.^900^

#### 7.4.2 Multimodal intervention

**FIGURE 4:**
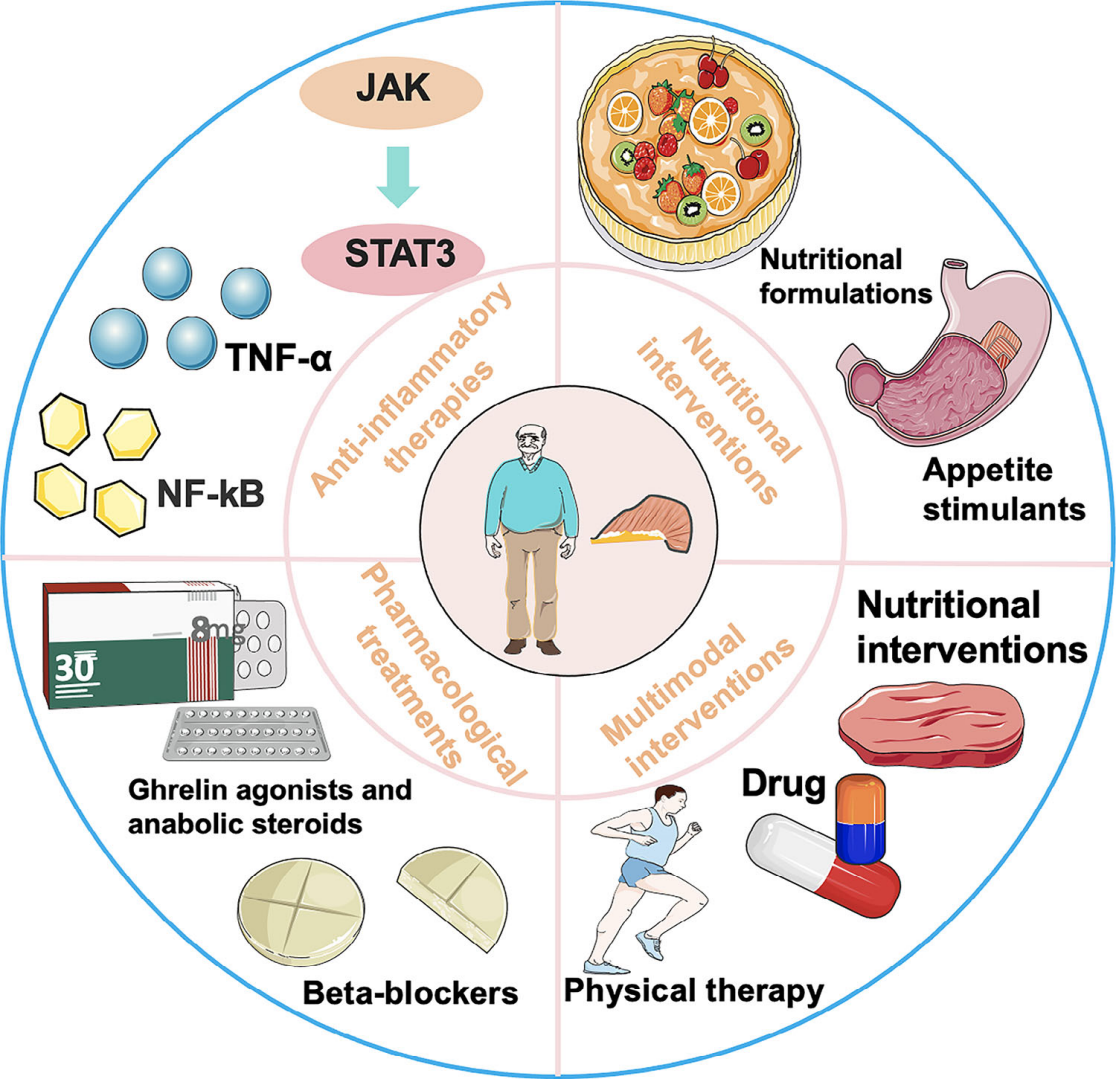
Novel anticachexia strategies. Therapeutic approaches for cachexia encompass anti‐inflammatory treatments, nutritional strategies, pharmacological options, and multimodal interventions.

As discussed, cancer cachexia is a complex metabolic disorder that is challenging to address with conventional nutritional therapy; thus, nutritional support tailored to its pathophysiology is crucial. The European Society for Clinical Nutrition and Metabolism and European Society for Medical Oncology guidelines recommend a multifaceted approach for the treatment of cancer cachexia, emphasizing the integration of nutrition, exercise, and pharmacotherapy.^901^, ^902^, ^903^ The evidence supporting nutritional counseling is also robust.^901^, ^904^, ^905^ In a randomized phase III trial involving 104 patients with advanced gynecologic cancer, a combination treatment of MA, l‐carnitine, celecoxib, and antioxidants was found to be more effective than MA alone in enhancing LBM, REE, fatigue, and QOL.^906^ Additionally, a multidisciplinary intervention that included omega‐3 polyunsaturated fatty acid nutraceuticals, exercise, and the anti‐inflammatory drug celecoxib was administered to 46 patients with lung and pancreatic cancer undergoing chemotherapy, effectively mitigating weight loss.^907^ Alongside anti‐inflammatory, metabolism‐enhancing, and appetite‐stimulating medications, high‐quality nutritional therapy and appropriate exercise tailored to each patient's physical capabilities can improve physical function and increase skeletal muscle mass.^908^, ^909^ Multimodal interventions are deemed effective and are currently under assessment in clinical studies, highlighting their increasing necessity in future treatments (Figure 4).

## 8 EMERGING THERAPIES AND FUTURE DIRECTIONS

Recent insights into the pathophysiology of sarcopenia and cachexia have revealed several promising drug targets and innovative treatments. These advancements offer potential solutions for addressing the global epidemic of sarcopenia and cachexia and their associated health challenges. In addition to pharmacological therapies, alternative approaches are being explored, including modulation of the gut microbiome, stem cell therapy, and gene therapy.

### 8.1 Gene therapy and molecular targets

Gene therapy represents a promising avenue for treating muscle wasting by specifically targeting and modifying the key molecular pathways involved in disease progression. This approach involves two methods: the first, known as the indirect method, enhances the secretion of therapeutic products derived from exogenous gene expression. The second method directly introduces target genes into cells using viral or nonviral vectors to express the desired functional proteins in vivo.

Viral vectors, such as adeno‐associated virus (AAV), are commonly used due to their low risk of genotoxicity and consistent long‐term expression.^910^, ^911^ AAV has been employed to deliver therapeutic genes in both preclinical and clinical trials.^912^ Among various AAV types, AAV9 is particularly notable for its high delivery efficiency to skeletal muscle. Among multiple types of AAVs, AAV9 is one of the most widely used vectors for treating muscle diseases for its high delivery efficiency in muscle.^913^, ^914^, ^915^ Studies have demonstrated that AAV9‐mediated gene delivery, including miR‐23a/27a, significantly protects against muscle force loss and reverses dystrophic features in rodent models.^916^ Additionally, AAV9‐mediated gene therapies have shown promise in improving muscle histology in young adult Duchenne muscular dystrophy dogs.^917^

Furthermore, muscle‐specific AAV9‐mediated overexpression of NLS–PGC‐1α4 has alleviated aging‐associated sarcopenia and metabolic dysfunction in mouse models. However, these therapies have yet to be tested in humans, and the main drawback is the lack of extensive clinical trials. Current findings primarily focus on muscle atrophy impacts without thorough safety evaluations, highlighting the need for further research.

### 8.2 Stem cell therapy for muscle regeneration

Stem cell therapy, also known as cellular therapy or cytotherapy, utilizes stem cells to prevent and treat diseases. The efficacy of this approach has been explored across various conditions.^918^, ^919^ In muscle tissue, satellite cells serve as the primary stem cells, typically located in the basal lamina or between muscle fibers, where they remain in a quiescent state. However, their self‐renewal ability is significantly diminished in patients with muscle atrophy.^920^ Therefore, increasing the number of satellite cells or enhancing their functionality could offer a promising theoretical solution for treating muscle atrophy.

In animal models, the transplantation of satellite cells into damaged muscle has demonstrated successful self‐renewal and regeneration.^921^, ^922^ Early human studies have also indicated a potential role for mesenchymal stem cells in managing frailty.^923^ Nonetheless, challenges such as cost, regulatory constraints, and ethical considerations must be carefully addressed to facilitate the clinical application of stem cell therapy.

### 8.3 Targeting gut microbiota and metabolism

Modulation of microbiome could impact muscle metabolism and inflammation. For example, sarcopenia has been associated with alterations in gut microbiota composition, including higher levels of Lactobacillus, lower levels of Lachnospira, reduced microbial diversity, and decreased richness of microbial genes.^924^, ^925^ Further, gut microbiota affects muscle properties that are fundamentally altered in cachexia by impacting muscle metabolism and contributing to inflammation. The gut microbiome is emerging as a new target for addressing sarcopenia and cachexia. Modulating the gut microbiota therapeutically could help prevent and treat metabolic disorders associated with aging.^926^, ^927^, ^928^ Selective modulation of the human gut microbiome offers a novel approach to treating sarcopenia. This can be done through dietary supplements like prebiotics and probiotics that impact bacterial growth, as well as through fecal microbiota transplantation.^929^, ^930^, ^931^, ^932^

Moreover, in the context of cancer cachexia, a synbiotic containing inulin‐type fructans and Lactobacillus reuteri has been shown to counteract disturbances in gut microbiota composition in cachectic mice.^933^ This intervention also positively affected cancer progression, morbidity scores, and survival rates. Several features associated with cachexia, such as loss of muscle mass, skeletal muscle expression of Cathepsin L and LC3, as well as markers of intestinal permeability and immune function, showed slight improvements following synbiotic administration.

Current preclinical studies have primarily focused on the impact of gut microbiota on body weight, muscle properties, and proinflammatory cytokines. However, there is limited information on the relationships between gut microbiota and food intake, adipose tissue metabolism, and IR—key factors in cancer cachexia and sarcopenia. It is crucial to gather more data on these drivers of cachexia and sarcopenia in future research, as novel treatment approaches are urgently needed.

### 8.4 Precision medicine approaches

Customized drug combinations tailored to the specific genetic and molecular profile of a patient's tumor or muscle can enhance efficacy while reducing toxicity. However, our current understanding of the genetic and epigenetic factors involved in sarcopenia and cachexia is limited. For instance, while muscle phenotypes exhibit strong heritability, the specific genetic factors underlying these traits remain unclear. Genetic factors alone do not solely determine the risk of muscle dysfunction; they interact with environmental influences, such as diet and physical activity, to shape individual risk.^934^

Understanding the potential connections between genes related to cachexia and sarcopenia could provide deeper insights into the pathogenesis of these two conditions. Ultimately, a patient‐centric approach is essential for managing sarcopenia and cachexia, highlighting the importance of personalized care strategies and patient education. Increasing awareness of these syndromes among patients, healthcare providers, and the general public is a crucial step toward developing more effective solutions to this growing clinical concern. Personalized therapeutic strategies based on genetic and molecular profiling are vital for improved outcomes.

## 9 CONCLUSION AND PERSPECTIVE

Cachexia is a complex systemic condition involving multiple metabolic pathways across various tissues and organs. It is characterized by systemic inflammation, progressive weight loss, and depletion of adipose tissue and skeletal muscle, which cannot be fully reversed through conventional nutritional support. Cachexia shares some similarities yet exhibits clear differences from other syndromes, such as age‐related muscle loss (sarcopenia), anorexia, malabsorption, hyperthyroidism, and starvation. Sarcopenia, a progressive and multifaceted disease, is associated with an increased burden of morbidity and mortality. While cachexia and sarcopenia can be defined as distinct clinical entities, they exhibit significant overlap, particularly in terms of inflammation and weakness. Since weakness/fatigue and inflammation are common features across various conditions, clinicians must look beyond these symptoms to identify unique characteristics of each condition, evaluating them within the context of a patient's clinical history and personal profile (e.g., previous diseases, mental health status, age). Early and accurate diagnosis is critical for the effective management of both sarcopenia and cachexia. Future research should prioritize the discovery and clinical application of new biomarkers for early detection. Advanced imaging techniques and molecular diagnostics can be employed to monitor disease progression and assess treatment responses. Reliable biomarkers will facilitate personalized treatment strategies, allowing for timely and targeted interventions to slow or halt disease progression.

**FIGURE 5:**
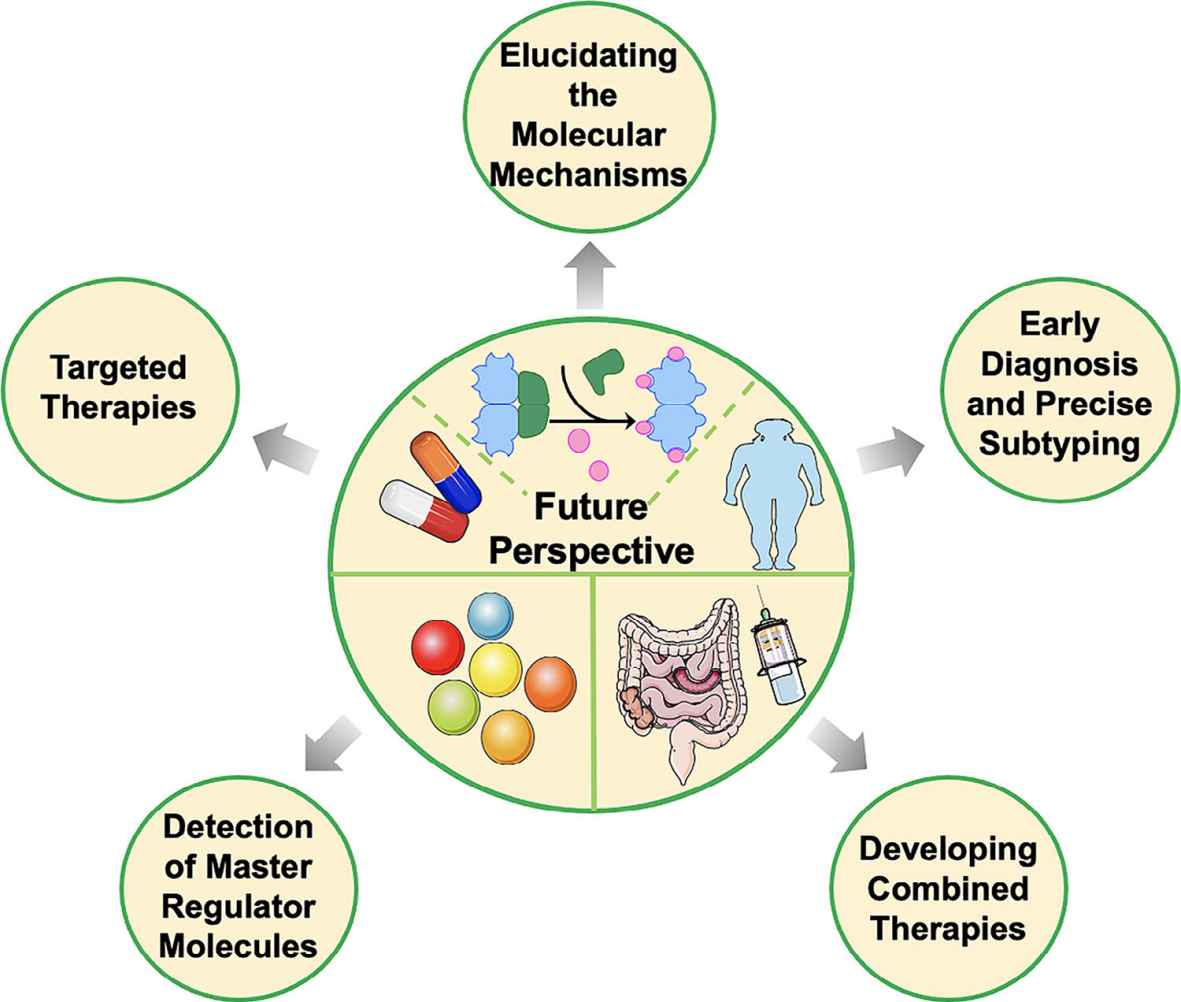
Future perspective. A comprehensive understanding of the mechanisms underlying sarcopenia and cachexia is essential. Recent advancements in science and technology allow us to evaluate these conditions at the tissue, cellular, and molecular levels. Early and accurate diagnosis is critical for the effective management of both sarcopenia and cachexia. Future research should prioritize the identification and clinical application of new biomarkers for early detection. Advanced imaging techniques and molecular diagnostics can be utilized to monitor disease progression and evaluate treatment responses. Given the complex factors contributing to sarcopenia and cachexia, reliance on a single treatment method is often inadequate. Therefore, a multifaceted treatment strategy that addresses various pathways and cellular interactions is necessary. Combining medications with cell and gene therapies, as well as employing nanoparticles and gene‐editing technologies, can enhance both the precision and effectiveness of treatments. Ultimately, identifying effective targeted therapies, key regulatory factors, and early diagnostic methods will be crucial for developing future treatments for sarcopenia and cachexia.

Understanding the pathophysiological foundations of cancer cachexia and sarcopenia is essential for distinguishing between the two conditions. This knowledge is crucial for establishing an early and accurate diagnosis and implementing timely therapeutic measures. Given the potential role of adipose tissue in the development of sarcopenia and cachexia, it is vital to investigate the interactions among different adipose depots (including intermuscular and intramuscular fat) and skeletal muscle, along with their associated molecular signaling pathways. This complex interplay involves various molecules, such as myokines, adipokines, lncRNAs, and miRs, that mediate the interactions between skeletal muscle and adipose tissue. The effects of these molecules on the homeostasis of both tissues may be closely linked to the molecular mechanisms underpinning sarcopenia and cachexia. Key questions remain regarding whether the regulation is direct or indirect—operating through an adipose–brain–muscle or an adipose–immune–muscle axis. Is there a master cytokine orchestrating these interactions? Are there “bystander” molecules that inadvertently function as “mediators”? Future studies should focus on defining the molecules produced by adipose and muscle cells and elucidating the intricate regulatory mechanisms involved. These considerations are vital for a comprehensive understanding of the therapeutic potential of these molecules in addressing sarcopenia and cachexia (Figure 5).

Due to the complex factors contributing to sarcopenia and cachexia, relying on a single treatment method is frequently insufficient. Consequently, a multifaceted treatment strategy that addresses various pathways and cellular interactions is essential. Combining medications with cell and gene therapies, as well as utilizing nanoparticles and gene‐editing technologies, can improve both the accuracy and effectiveness of treatments. Exploring the synergistic effects of different therapies could result in better outcomes and fewer side effects. Tailoring therapeutic approaches based on genetic and molecular profiles is crucial for achieving enhanced results.

## AUTHOR CONTRIBUTIONS

Tiantian Wang was a major contributor to writing the manuscript, creating all figures and tables, and conducting the literature search. Tiantian Wang, Dong Zhou, and Zhen Hong made significant contributions to the manuscript's design and critically revised it for important intellectual content. All authors have read and approved the final version of the manuscript.

## CONFLICT OF INTEREST STATEMENT

The authors declare that they have no known competing financial interests or personal relationships that could have appeared to influence the work reported in this paper.

## ETHICS STATEMENT

Not applicable.

## Data Availability

Not applicable.
